# Oxytoxaceae are prorocentralean rather than peridinialean dinophytes and taxonomic clarification of heterotrophic *Oxytoxum lohmannii* (≡ “*Amphidinium*”* crassum*) by epitypification

**DOI:** 10.1038/s41598-024-56848-y

**Published:** 2024-03-20

**Authors:** Marc Gottschling, Stephan Wietkamp, Alexis Bantle, Urban Tillmann

**Affiliations:** 1 Department Biologie―Systematik, Biodiversität und Evolution der Pflanzen, GeoBio-Center, Ludwig-Maximilians-Universität München, Menzinger Str. 67, 80 638 München, Germany; 2 Alfred-Wegener-Institute, Helmholtz Centre for Polar and Marine Research, Am Handelshafen 12, 27 570 Bremerhaven, Germany

**Keywords:** Marine biology, Molecular evolution, Taxonomy

## Abstract

During evolution of Dinophyceae, size reduction of the episome has occurred in several lineages (including unarmoured Amphidiniales and armoured Prorocentrales). One such species is *Amphidinium crassum*, whose taxonomic identity is elusive though showing morphological similarities with Oxytoxaceae (currently placed in armoured Peridiniales). Plankton samples were taken at the type locality of *A. crassum* in Kiel Bight (Baltic Sea) in order to establish monoclonal strains. The protist material was examined in detail using light and electron microscopy, and a long (2984 bp) ribosomal RNA sequence gained was part of a taxon sample comprising 206 specimen vouchers and representing the known molecular diversity of Dinophyceae. Cells of *A. crassum* were ovoid and exhibited a plate pattern po, 4′, 1a, 6′′, 5c, 4s, 5′′′, 1′′′′. In the molecular phylogeny, the species seemed to belong neither to Amphidiniales nor to Peridiniales but to Prorocentrales and clustered with other representatives of Oxytoxaceae. The morphological diversity of Prorocentrales appears thus expanded, and the group may include a number of previously unrecognised representatives unusually having five postcingular and only a single antapical plate. The taxonomic identity of *A. crassum* is clarified by epitypification, and the species notably exhibits both an apical pore and an additional epithecal pore.

## Introduction

Dinophytes are a diverse group of unicellular protists occurring in marine and freshwater habitats worldwide. Their diversity assessment started in the mid eighteenth century and currently includes some 2500 extant species^1^. In the last decades, refined techniques (such as scanning and transmission electron microscopy: SEM and TEM) in combination with molecular phylogenetics have greatly improved the knowledge of dinophyte diversity and evolution and have also led to major taxonomic rearrangements. This is particularly true for the large and heterogeneous group of unarmoured (athecate or “naked”) dinophytes, for which traditional morphological traits, such as the displacement and position of the cingular groove, became more and more ineffective to identify species or monophyletic assemblages. Consequently, new ultrastructural traits, primarily the apical groove system (or apical furrow apparatus or acrobase^2,3^), have been identified to better reflect phylogenetic relationships of unarmoured dinophytes.

As in other groups of the microscopy realm, knowledge of the phylogenetic relationships in dinophytes is largely based on the comparison of sequences gained from the rRNA operon. Analyses of next-generation sequencing (NGS) data have shown important evolutionary transformations within the group (such as the unique origin of a cell wall constituting of cellulose plates^4,5^), but the available taxon sample of such studies is still extremely limited. Only the data pool of ribosomal RNA (rRNA) sequences has grown to such an extent that meaningful taxon samples for phylogenetic analyses can be compiled. However, this advantage does not prevent the problems that arise in the inference of phylogeny due to the enormous rate heterogeneity of rRNA sequences^6–9^. There are cases of close relationship between taxa with very high and very low substitution rates, which may sometimes lead to artificial phenomena such as long-branch attraction^10^ and disturb phylogenetic inference.

In the past, separate analyses of SSU, ITS or LSU sequences were mostly carried out, not only but also in dinophytes and often with correspondingly disappointing results. However, the individual segments of the rRNA operon do not evolve independently of each other but concertedly^11^, so that the concatenation of existing and sometimes independently generated sequences is appropriate (albeit a complex and laborious procedure). Sequences should normally only be concatenated strain by strain, as taxonomic identifications in GenBank are often only provisional if not even incorrect. The effort is worthwhile, however, because it is now possible to reflect morphologically recognised and established major groups of dinophytes in rRNA phylogenies as well^12–15^. These include the Dinophysales, Gonyaulacales, Gymnodiniales, Peridiniales, Prorocentrales and †Suessiales.

Among the unarmoured dinophytes such as *Gymnodinium* F.Stein and *Gyrodinium* Kof. & Swezy, species assigned to *Amphidinium* Clap. & J.Lachm. have seen a larger number of altering interpretations and taxonomic combinations. Historically, the name was broadly applied for unarmoured dinophytes with an episome smaller than the hyposome, but it was assumed early in history that this concept is largely artificial^16–18^. The DNA sequence comparison of the past years has then demonstrated the confusing heterogeneity of dinophytes filed under *Amphidinium*. The clade including the type species, *Amphidinium operculatum* Clap. & J.Lachm., and exhibiting a minute, crescent-shaped or triangular episome^19^, comprises only a small subset of ca 20 species^20–22^. Remaining species have been assigned to some 15 alternate, mostly unarmoured taxa such as *Apicoporus* Sparmann, B.S.Leander & Hoppenrath, *Bindiferia* Borchhardt, Chomérat, Sh.Murray & Hoppenrath, *Kapelodinium* Boutrup, Moestrup & Daugbjerg, *Nusuttodinium* Y.Takano & T.Horig., *Prosoaulax* Calado & Moestrup, Togula M.F.Jørg., Sh.Murray & Daugbjerg or *Testudodinium* T.Horig., Maiko Tamura, Katsumata & A.Yamaguchi. The presence of thin thecal plates in some other, re-investigated organisms indicate the distant relationship to *Amphidinium*, and those species have been transferred into armoured taxa such as *Adenoides* Balech, *Pseudadenoides* F.Gómez, R.Onura, Artigas & T.Horig. or *Thecadinium* Kof. & Skogsb.^23–25^. However, the taxonomic identity is still unclarified for a large number of species currently assigned to *Amphidinium*.

The three species *Amphidinium crassum* Lohmann, *Amphidinium longum* Lohmann and *Amphidinium rotundatum* Lohmann have been described from Kiel Bight in the German Baltic Sea^26^. The latter is one of those species having thin thecal plates, why it is identified today as species of *Heterocapsa* F.Stein^27^. However, the morphology of neither *A. crassum* nor *A. longum* has been studied in detail since over a century. Based on the protologues the cells of both species have a small episome and a conical through spherical, larger hyposome and no chloroplasts but shiny or coloured inclusions. The species are of similar size (27 and 25 µm in length for *A. crassum* and *A. longum*, respectively) and considered morphologically very similar, but the more frequent *A. crassum* is much broader and has a round antapex, whereas rarer *A. longum* is slender with an acute antapex.

Detailed morphological knowledge of *A. crassum* and *A. longum* currently is absent, but their general shape is distinct. Therefore, both species were repeatedly reported in field samples, and were used in various laboratory studies (and more specific literature is found in the Supplementary information). Doubts on the correct systematic placement of *A. crassum* and *A. longum* in *Amphidinium* were raised early in history^28,29^, and a relationship to *Oxytoxum* F.Stein based on the general shape of the cells was rather assumed^16^. Likewise, the reminiscence of small *Amphidinium* species, having a vertical cingulum and a circular outline, with thecate *Oxytoxum* was emphasised (explicitly noting *A. crassum* and *A. longum*^17^).

In their current circumscription, the Oxytoxaceae comprise some 50 species of thecate dinophytes assigned to *Corythodinium* Loebl. & A.R.Loebl. and *Oxytoxum* with a relatively small epitheca, which are widespread mainly in warm open oceanic waters^30–32^. A rare trait of Oxytoxaceae, shared with only a few other dinophytes, is the configuration of the hypotheca exhibiting a single antapical plate only. Several other planktonic as well as sand-dwelling taxa (e.g., *Amphidiniopsis* Wołosz., *Centrodinium* Kof., *Planodinium* R.D.Saunders & J.D.Dodge, *Pseudadenoides*, *Roscoffia* Balech, *Sabulodinium* R.D.Saunders & J.D.Dodge, *Schuettiella* Balech) previously assigned to Oxytoxaceae^33,34^ are excluded or are at least only under debate to be included in Oxytoxaceae either based on morphological^35^ or molecular evidence^31,32^. The only available (SSU) sequence data of four oxytoxacean species support the taxonomic split between *Corythodinium* and *Oxytoxum*, but their placement within dinophytes remains elusive^31^.

In this study, we present the morphology of a species that is consistent with the protologue of *A. crassum* based on material collected at the type locality at Kiel Fjord in Germany. The plate pattern and gained rRNA sequences indicate a relationship to Oxytoxaceae, which appear as part of the Prorocentrales in a comprehensive molecular phylogeny rather than the Peridiniales, to which they are currently assigned. Species formerly assigned to *Amphidinium* are scattered over the dinophyte tree that a broad and representative taxon sample is necessary for the phylogenetic placement of *A. crassum* and its relatives. The present single antapical plate and contact of the first and last postcingular plates, both very unusual characters within dinophytes, is shared with a number of taxa of previously uncertain phylogenetic position, which are now identified as members of the Prorocentrales as well. We also discuss the possible synonymy of *A. crassum* and *A. longum* likely identifying the same species of *Oxytoxum*.

## Results

### General morphology and behaviour

**Figure 1:**
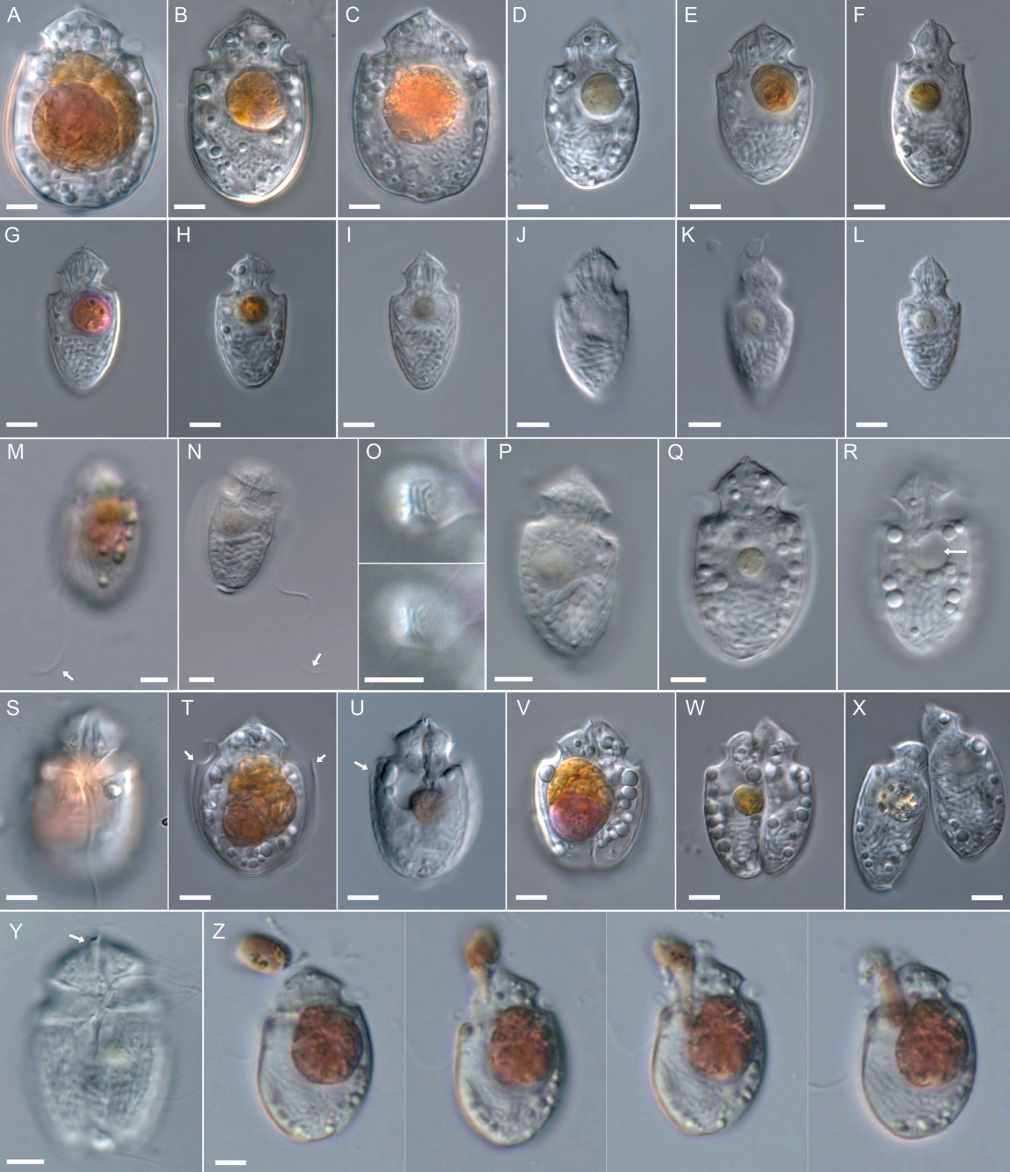
*Oxytoxum lohmannii* (strain K-AC-E10), light microscopy of living cells. (**A**–**L**) Size and shape of different cells in ventral/dorsal view. (**M**–**N**) Two different cells indicating length of the longitudinal flagellum (arrow). (**O**) Two focal planes of an apical view. (**P**) Cell in lateral view, note the antapical position of the nucleus and the transverse striation of chromosomes. (**Q**–**R**) Two focal planes of the same cell, note the presence of a pusule just below the cingulum (arrow in **R**). (**S**) Cell in ventral view, note the insertion of the longitudinal flagellum and the peduncle canal in the central sulcal area and the episome. (**T**–**U**) Slightly squeezed cells revealed the presence of thecal plates (arrows). (**V**–**X**) Different stages of vegetative cell division. (**Y**) Cell in ventral view, note the extruded peduncle (arrow). (**Z**) Single frames of a cell ingesting a cell of *Rhodomonas salina*. Scales bars = 5 µm.

Cells of *Oxytoxum lohmannii*, *nom. nov. pro* “*Amphidinium*” *crassum*, moved in a characteristic way. They swam relatively slowly on a straight path without or with rather slow rotation around their own axis. This movement was regularly interrupted by phases with few, or a couple of short and abrupt, backward movements (Video S1). Cells had two flagella, and the wavy transverse flagellum in the cingulum completely surrounded the cell. The longitudinal flagellum emerged below the cingulum (Figs. 1S, 5I) and was directed posteriorly. Its length was about 1.5-fold of the body length (Fig. 1M, N).

**Figure 2:**
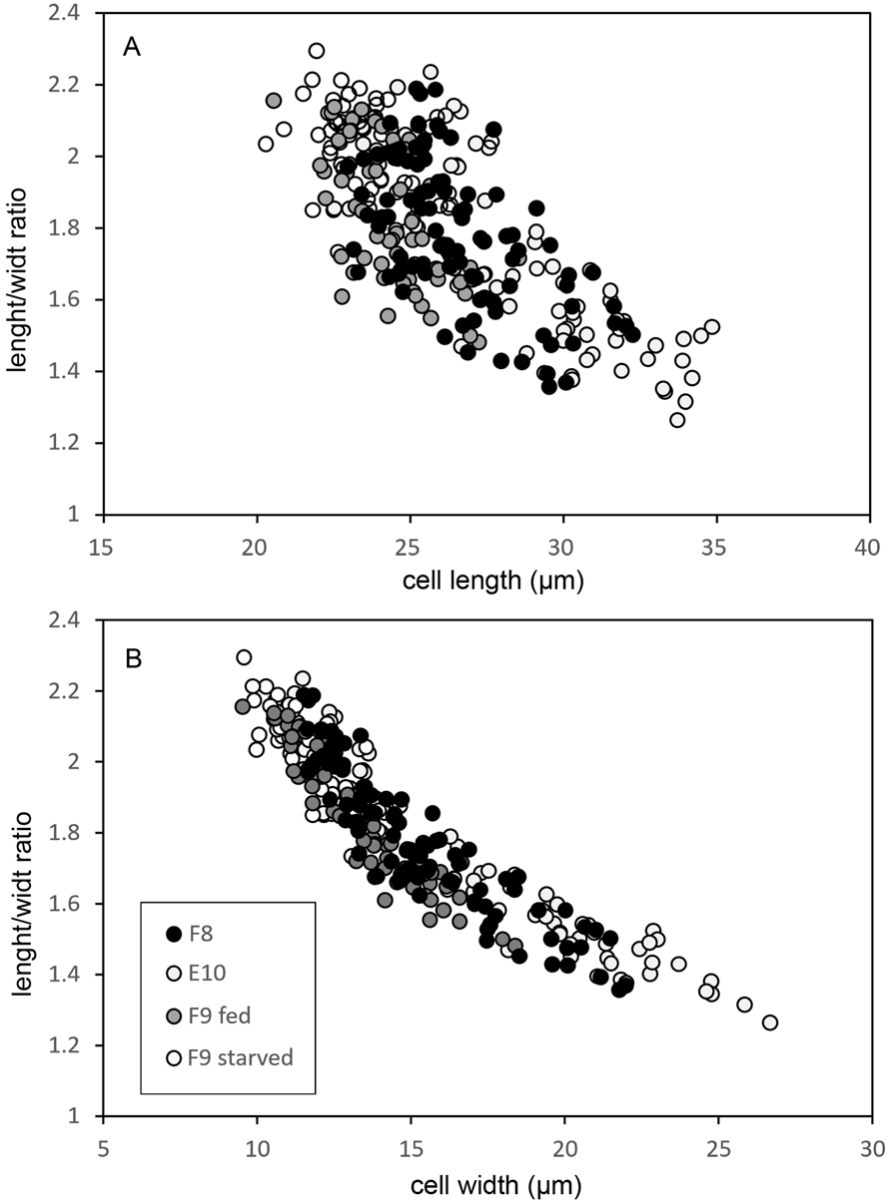
Scatterplot of morphometric size measurements of different strains (F8, K-AC-E10, F9 fed and starved) of *Oxytoxum lohmannii*. (**A**) Cell length (µm) and (**B**) cell width (µm) versus length/width ratio.

The cell shape and size (Fig. 1A–L) were very variable, most likely depending on the nutritional state. Generally, cells were (widely) ovate to (narrowly) elliptic in ventral view, with no or very little dorso-ventral compression. The episome was small and contributed 10–20% to total cell length. It had the shape of a symmetric cone, whose width at the base was 50–78% of total cell width. Cells had an acuminate apex, which terminated in a pair of short crests (Fig. 1C, J, O, U). The cingulum was wide (9–18% of total cell length), circular and deeply excavated. The hyposome was large (65–88% of total body length). For a number of cells, the hyposome had almost parallel sides in the anterior half (Fig. 1B, D, F). Posteriorly, the antapex was widely rounded in broad cells (Fig. 1A), but obtuse or slightly acute in more slender cells (Fig. 1J‒K). The sulcus significantly extended into the hyposome, where it was visible as a semi-tubular inset structure (Fig. 1R–S). The course of the sulcus into the episome was difficult to identify in LM, but a small indentation running upwards for a short distance was visible, from the area where the longitudinal flagellum emerged (Fig. 1S). The size of cells covered a wide range. Cell length and width ranged from 20.3 to 34.9 µm and 9.5 to 26.7, respectively (Fig. 2), with small and slender cells dominating in starved populations. The ratio of length to width likewise covered a wide range from 1.26 to 2.29 (Fig. 2).

The large nucleus with thick chromosomes was located posteriorly (Fig. 1B–L). Occasionally, chromosomes revealed a pronounced transverse striation (Fig. 1P). There were no obvious chloroplasts, but a large food inclusion of varying size and colour (depending on the nutritional state) was present and consistently located above the nucleus (Fig. 1). Occasionally, the food body was exceptionally bright and shining (Fig. 1C, S). A rounded hyaline area, presumably a pusule, was occasionally seen in the hyposome directly below the cingulum (Fig. 1Q‒R). A varying number of other small, round and strongly refractive bodies were randomly distributed in the cell (Fig. 1A, B, D, Q, R). Bundles of rod-like structures (presumably trichocysts) were visible in the upper area of the cell (Fig. 1D, E, G, I) and in the periphery of the hyposome (Fig. 1G, I). When cells were slightly squeezed under the coverslip, thin thecal plates (Fig. 1T) and their surface structure (Fig. 1U) became visible.

Cells divided in the motile stage by desmochisis (Fig. 1V–X). The plane of division on the hyposome run almost perpendicularly to the cell’s longitudinal axis (Fig. 1V) but on the episome, the fission line was oblique (Fig. 1W). In the final stage of division, both daughter cells were loosely connected in the anterior region (Fig. 1X). Cells were phagotrophic by means of a feeding tube (peduncle). The peduncle was located in the episome and cingular area of the cell and was extruded through the narrow, tube-like sulcal canal in the episome (Fig. 1Y, Video S1). For feeding, cells with an extruded peduncle attached to a single cell of the food alga, which subsequently was drawn through the peduncle inside the grazer cell (Fig. 1Z, Video S1). The whole feeding process lasted for about 25–55 s (n = 7 observations). Cells of the food algae were taken up completely, as no visible remains were left behind (Video S1).

### Thecal plate pattern

**Figure 3:**
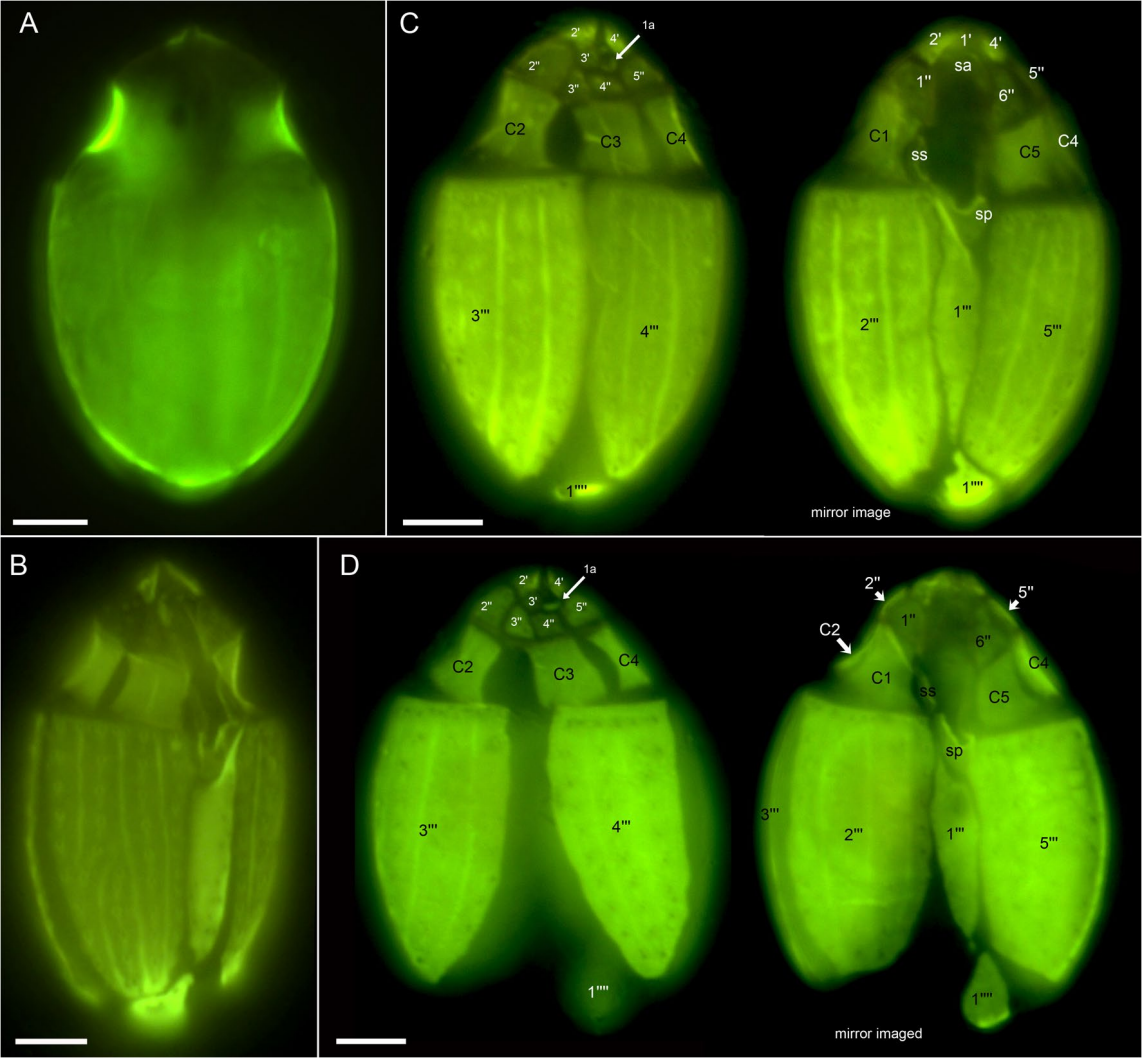
*Oxytoxum lohmannii* (strain K-AC-E10), light microscopy of formaldehyde fixed cells stained with Solophenyl Flavine and viewed with epifluorescence and green light excitation. (**A**–**B**) Cells in ventral view. (**C**–**D**) Two focal planes each of squeezed cells in dorsal view; note that the corresponding ventral view (right part) is mirror imaged. Plate labels according to the Kofoidean system. Sulcal plate labels: sp, posterior sulcal plate; ss, left sulcal plate. Scale bars = 5 µm.

**Figure 4:**
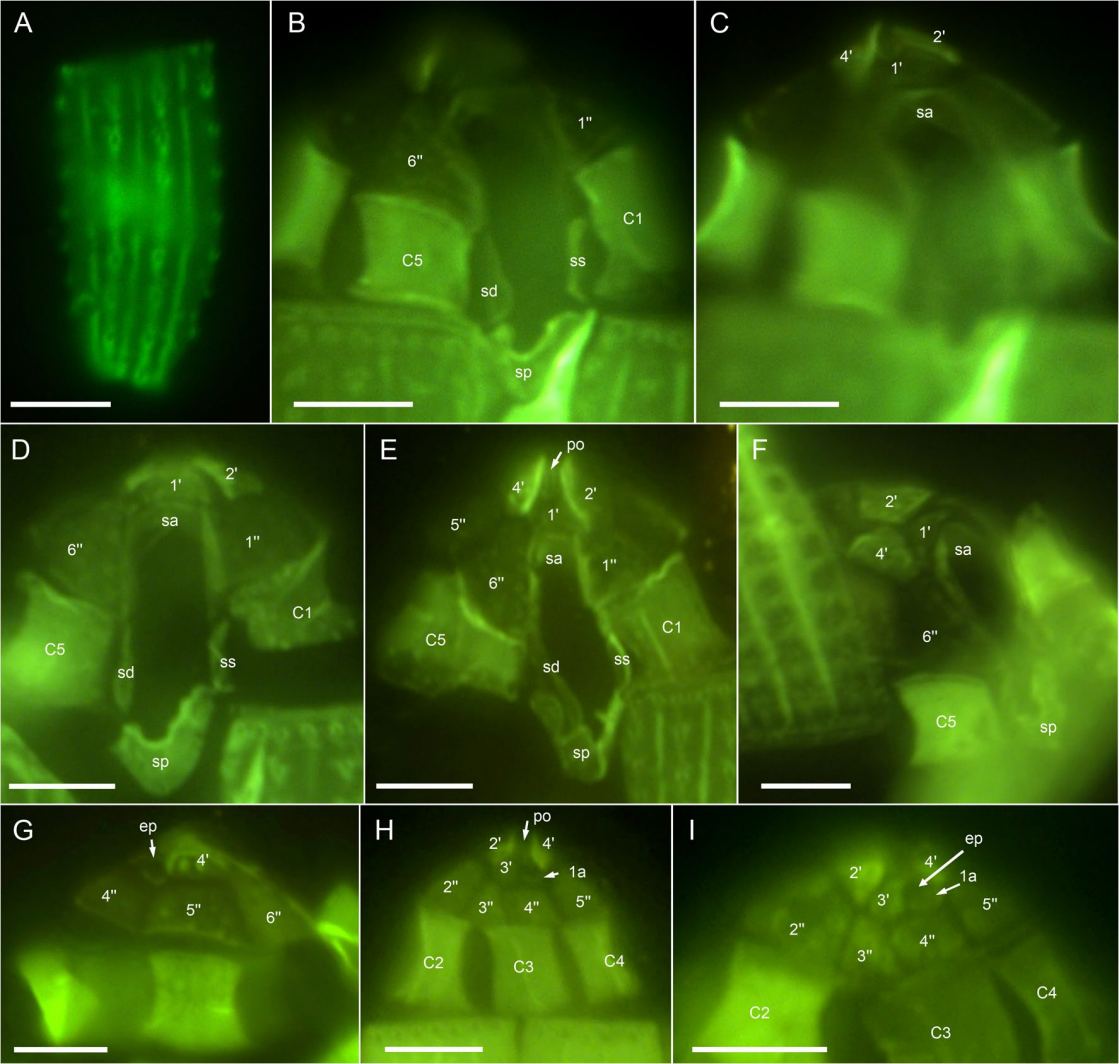
*Oxytoxum lohmannii* (strain K-AC-E10), detailed light microscopy view of formaldehyde fixed cells stained with Solophenyl Flavine and viewed with epifluorescence and green light excitation. (**A**) One of the large postcingular plates, note the large thecal pores and the plate surface ornamentation. (**B**–**F**) Arrangement of epithecal and sulcal plates in ventral (**B**–**E**) and lateral (**F**) view. (**G**–**I**) Arrangement of epithecal plates in dorsal view. Plate labels according to the Kofoidean system. ep, epithecal pore; Sulcal plate labels: sa, anterior sulcal plate; sd, right sulcal plate; ss, left sulcal plate; sp, posterior sulcal plate. Scale bars = 5 µm.

**Figure 5:**
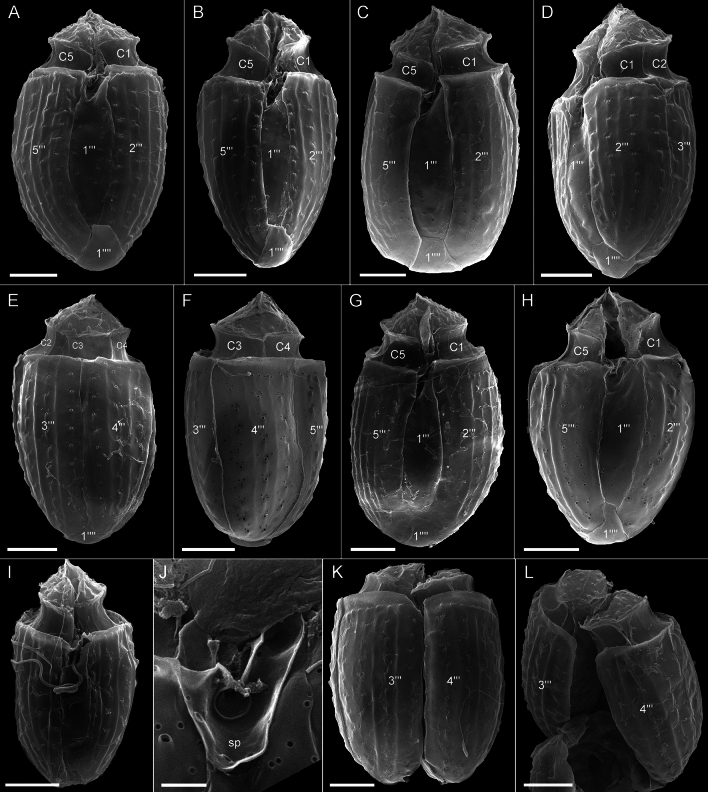
*Oxytoxum lohmannii* (strain K-AC-E10), SEM of different cells. (**A**–**I**) Cells in ventral (**A**–**C**, **G**–**I**), in left lateral (**D**) or in dorsal view (**E**–**F**); note the peduncle canal in G and H, and the insertion of the longitudinal flagellum in I. (**J**) Detailed view of the posterior sulcal plate. (**K**–**L**) Two different cells in dorsal view in division; note that in the epitheca all apical plates are allocated to the left daughter cell. Plate labels according to the Kofoidean system. Sulcal plate labels: sp, posterior sulcal plate. Scale bars = 5 µm (**A**–**I**, **K**–**L**) or 1 µm (**J**).

**Figure 6:**
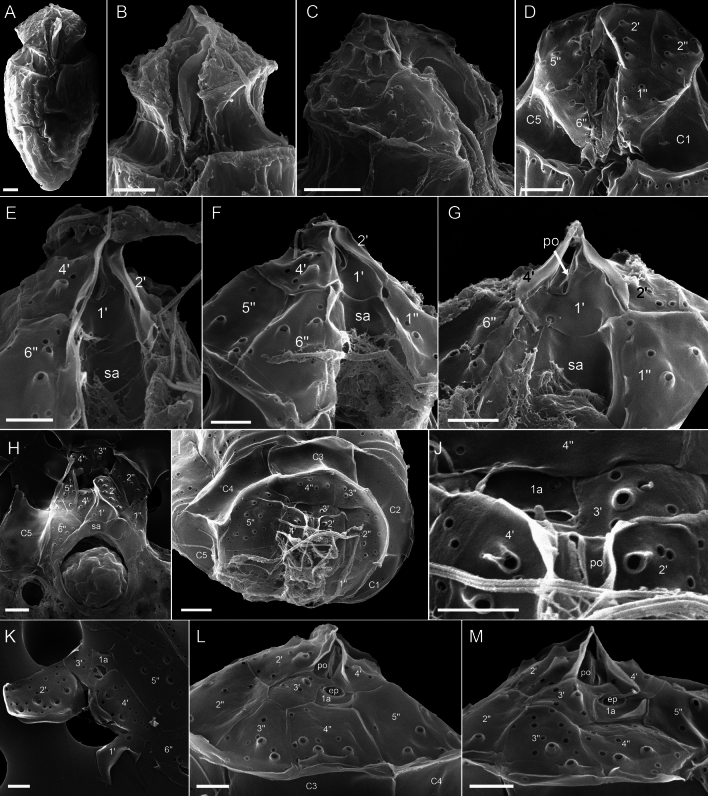
*Oxytoxum lohmannii* (strain K-AC-E10), SEM of different cells. (**A**) Cell with extruded peduncle inside the peduncle canal. (**B**–**C**) Detailed view of the peduncle canal area in ventral (**B**) and lateral view (**C**); note that the peduncle is visible in B. (**D**–**G**) Detailed view of epitheca and sulcal area in ventral-apical (**D**) and ventral view (**E**–**G**). (**H**–**J**) Epithecal plates in apical view; note that J is an enlarged view of the cell shown in I. (**K**) Internal view of apical plates; note that the pore plate probably is disrupted. (**L**–**M**) Epithecal plates in dorsal view. Plate labels according to the Kofoidean system. ep, epithecal pore; sa, anterior sulcal plate. Scale bars = 2 µm (**A**–**D**, **H**–**I**) or 1 µm (**F**–**H**, **J**–**M**).

**Figure 7:**
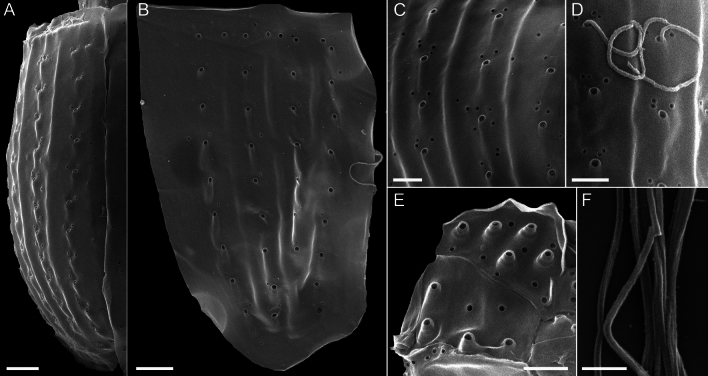
*Oxytoxum lohmannii* (strain K-AC-E10), SEM. (**A**–**D**) Thecal pores and surface ornamentation of precingular plates; note that mucocyst-like material is extruded from two of the large pores. (**E**) Thecal pores on epithecal plates. (**F**) Bundles of ejected material. Scale bars = 2 µm (**A**–**B**) or 1 µm (**C**–**E**) or 0.5 µm (**F**).

**Figure 8:**
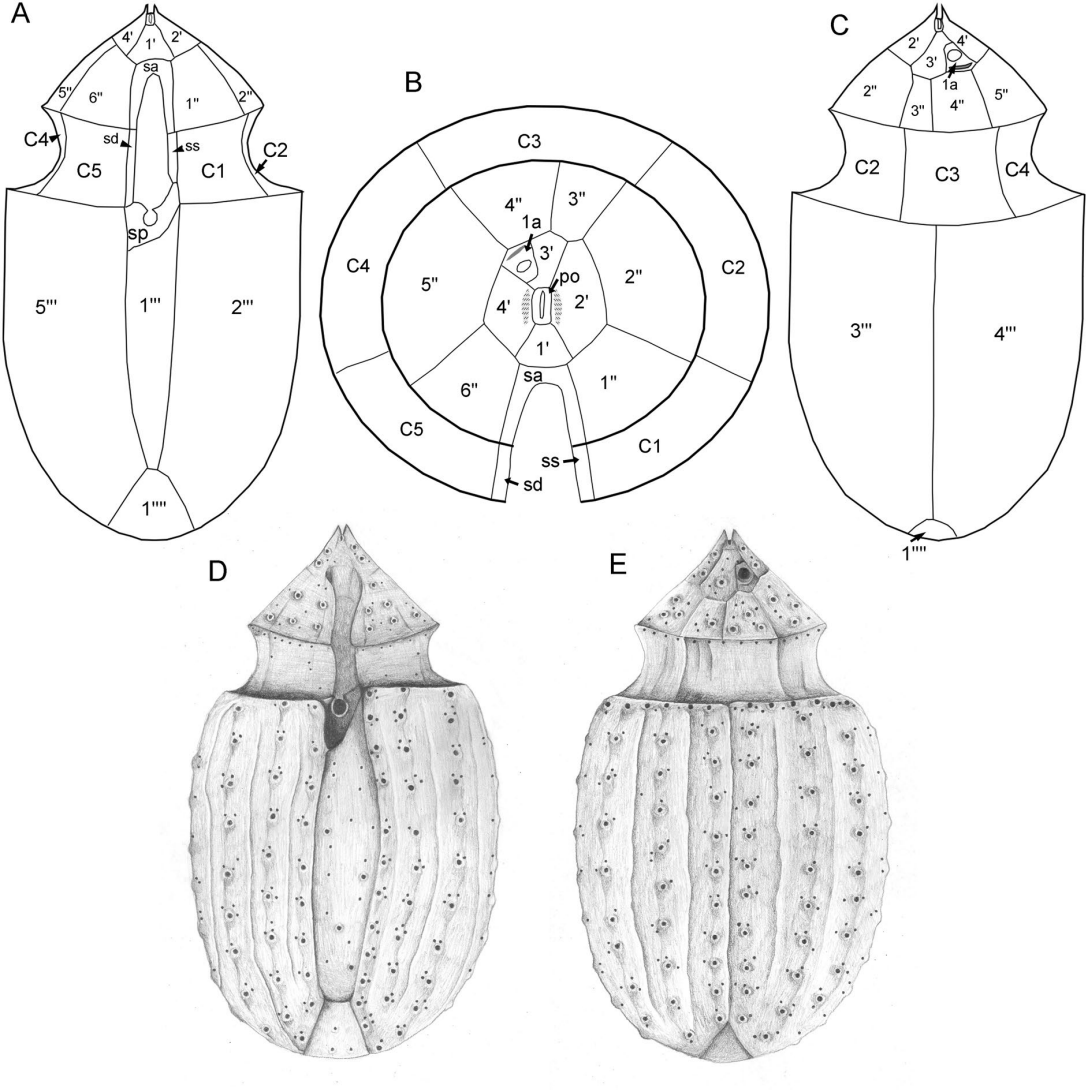
Drawings of *Oxytoxum lohmannii* plate pattern. (**A**–**C**) Schematic line drawings of the plate pattern in ventral view (**A**). (**B**) Epithecal plates in apical view. (**C**) Dorsal view. (**D**–**E**) Schematic drawing of a whole cell in SEM in ventral (**D**) and dorsal (**E**) view.

The presence of thecal plates could already be adumbrated by regular LM, but detailed studies of the plates to resolve the number and arrangement of thecal plates required fluorescence microscopy (Figs. 3 and 4) and SEM (Figs. 5, 6 and 7). Combining both microscopy techniques, the tabulation was determined as po, 4′, 1a, 6′′, 5c, 4(?)s, 5′′′, 1′′′′ (Fig. 8A–E). There were five large postcingular plates (Figs. 3 and 5A–H). The ventral plate 1′′′ was narrower than the other postcingular plates and was asymmetric and sharply elongated on the cell’s left side towards the cingulum (Figs. 3B–D and 5A‒B, G‒H). A single plate formed the antapex, which was broadly truncated or slightly acute ventrally towards the first postcingular plate (Figs. 3B–D and 5A–H) and triangular or truncated on the cell’s dorsal side (Figs. 3C and 5E‒F).

The broad cingulum was composed of five plates, which were similar in size (Figs. 3C‒D, 5A–H and 6I). In the ventral area of the epitheca, the sulcal plates formed a caved and funnel-shaped emergence side of the peduncle (Fig. 6A–D). Shape and location of sulcal plates were difficult to observe and to identify. The posterior sulcal plate (sp) was characteristic in shape, having a rounded posterior part and an asymmetric anterior part with an elongated left side. On the plate sp, there was a semi-circular notch in the middle (Figs. 3B‒C, 4B, D, 5I, J), from which the flagella emerged (Fig. 5I). The anterior sulcal plate (sa) was smooth, tubularily curved and located below the first apical plate (Figs. 4C–F and 6E–G). In SEM, other sulcal plates in the tubular sulcal area were impossible to reveal but in fluorescent microscopy, a left and a right sulcal plate connecting the posterior and anterior sulcal plates were visible (Fig. 4B, D, E). Both left and right sulcal plates were narrow and as long as the adjacent cingular plates. Structure of the central sulcal area remained elusive.

The epitheca was composed of twelve small plates (Figs. 3C, D, 4B–I and 6D–M). There were four apical plates. The two lateral apical plates 2′ and 4′ were larger compared to the ventral plate 1′ and dorsal plate 3′. Both lateral apical plates terminated towards the apex with a dorso-ventrally orientated and upwards-bound ridge, and both ridges formed the double-pointed termination of the epitheca (Fig. 6G, M). The first apical plate was triangular and located between plates 2′ and 4′ (Fig. 6E–G). In the centre of the apex, there was a narrow pore plate (Fig. 6G, J, L, M). This plate was hidden in most cases by the upward bound ridges of plates 2′ and 4′. The exact shape of the apical pore opening was thus difficult to document. At least, Fig. 6G and J indicates that the pore plate had a central, elongated slit. The dorsally located plate 3′ was very small and was next to the single anterior intercalary plate 1a (Figs. 4G–I and 6K–M). High magnification SEM revealed that there was a narrow anterior part of plate 3′ contacting plate 4′ and separating plate 1a from the pore plate (Fig. 6L–M). There was a conspicuous, round or elliptic and large pore (diameter: 0.44–0.72 µm; mean 0.61 ± 0.07 µm; n = 20) between plates 1a, 3′ and 4′, surrounded by a plate-like and ring-shaped structure (Fig. 6J–M). This pore is here denominated as epithecal pore (ep). Plate 1a usually had a small rim towards the postcingular plates 4′′ and 5′′ (Fig. 6L–M). Among the six plates of the precingular series, plate 3′′ war narrower compared to the others, and the lateral plates 2′′ and 5′′ were the widest (Figs. 3C–D, 4D–I and 6D–M).

Most thecal plates had pores of different size. Postcingular plates 2′′′–5′′′ had three or four longitudinal rows of eight to twelve large pores (diameter 0.18–0.28 µm; mean 0.21 ± 0.02 µm; n = 20) (Figs. 4A, 5A–I and 7A‒B). These pores were slightly tubular and inclined upwards leading to the impression of a longitudinal structure of the plates (Fig. 7A–D). Moreover, there was a small, irregularly wavy elevation on the plates between each row of pores, which contributed to the ornamentation of postcingular plates. A number of small pores (diameter 0.08–0.13 µm; mean 0.11 ± 0.02 µm; n = 20) was irregularly scatted around the large tubular pores (Fig. 7A, C, D) although exceptionally, only few of these small pores were present (Fig. 7B). On plate 1′′′, there were only few large pores, and they were not arranged in rows (Fig. 5A–C, B, G–I). Occasionally, large pores were still attached by extruded, round fibres in SEM (Fig. 7D) with a diameter of ca 110 nm. Large bundles of fibrous trichocyts were very commonly observed in SEM, which had a slightly lower diameter of 80–90 nm and a ripped and densely striated structure (Fig. 7F). On plates 2′ to 4′ and all precingular plates, there were one to eight tubular pores, which were slightly smaller and more variable in elongation and diameter (0.12–0.21 µm; mean 0.16 ± 0.02 µm; n = 20) than large pores on the postcingular plates. A number of small pores (diameter 0.08–0.13 µm; mean 0.11 ± 0.02 µm; n = 20) were scattered on most epithecal plates as well (Figs. 6D–M and 7E). Exceptionally for epithecal plates, there was no pore (other than the ep) on the anterior intercalary plate (Fig. 6L‒M) and on the first apical plate, there was only a single small pore (Fig. 6G). Cingular plates only had a limited number of small pores, which usually formed a small row below the anterior rim of each plate (Fig. 5C, D). Most sulcal plates could not be resolved in SEM, but there were a few pores on the posterior sulcal plate (Fig. 5J).

### Field material of *Oxytoxum*

**Figure 9:**
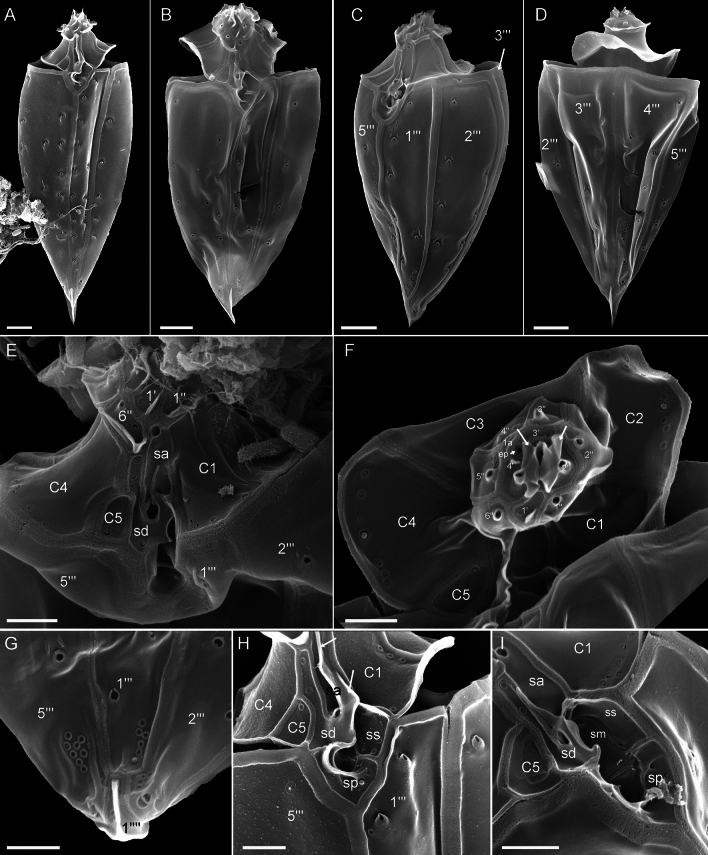
*Oxytoxum gladiolus*, SEM of field sample specimens. (**A**–**D**) Cells in ventral (**A**–**B**), left lateral (**C**) and dorsal view (**D**). (**E**, **F**) Detailed view of cingulum and epithecal plates in ventral (**E**) and apical view (**F**). (**G**) Antapex in ventral view. (**H**–**I**) Detailed view of the sulcal area in ventral view. Plate labels according to the Kofoidean system. ep, epithecal pore. Sulcal plate labels: sa, anterior sulcal plate; sd, right sulcal plate; ss, left sulcal plate; sm, median sulcal plate; sp, posterior sulcal plate. Scale bars = 2 µm (**A**–**D**) or 1 µm (**E**–**I**).

**Figure 10:**
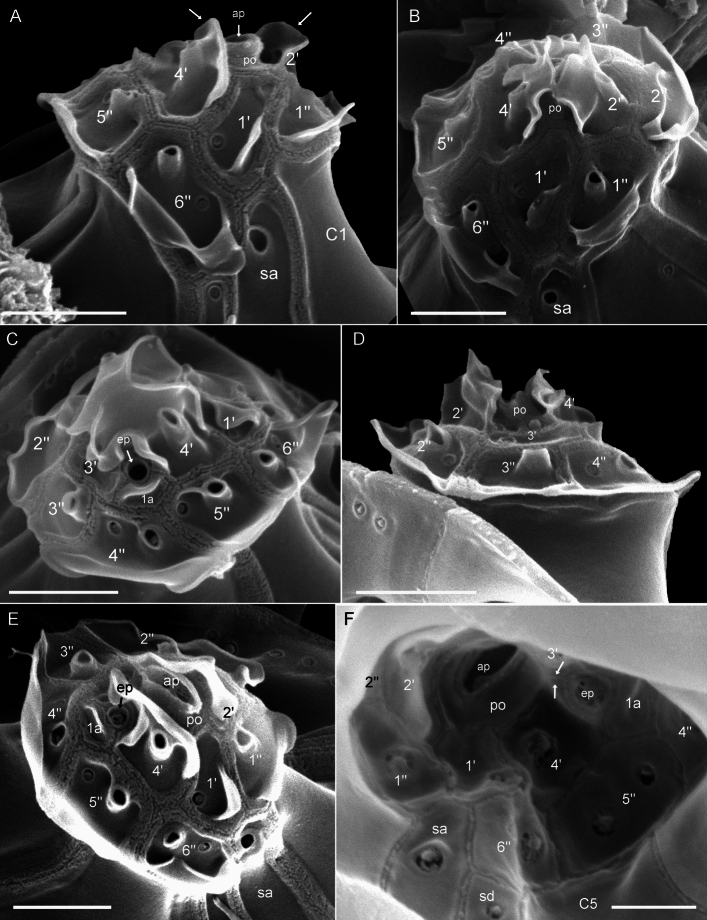
*Oxytoxum gladiolus*, SEM of field sample specimen. (**A**–**F**) Detailed views of epithecal plates in ventral (**A**), ventral apical (**B**), right lateral dorsal (**C**), dorsal (**D**), right lateral apical (**E**) and internal view (**F**). Plate labels according to the Kofoidean system. ep, epithecal pore. Sulcal plate labels: sa, anterior sulcal plate; sd, right sulcal plate. Scale bars = 1 µm (**A**–**E**) or 0.5 µm (**F**).

**Figure 11:**
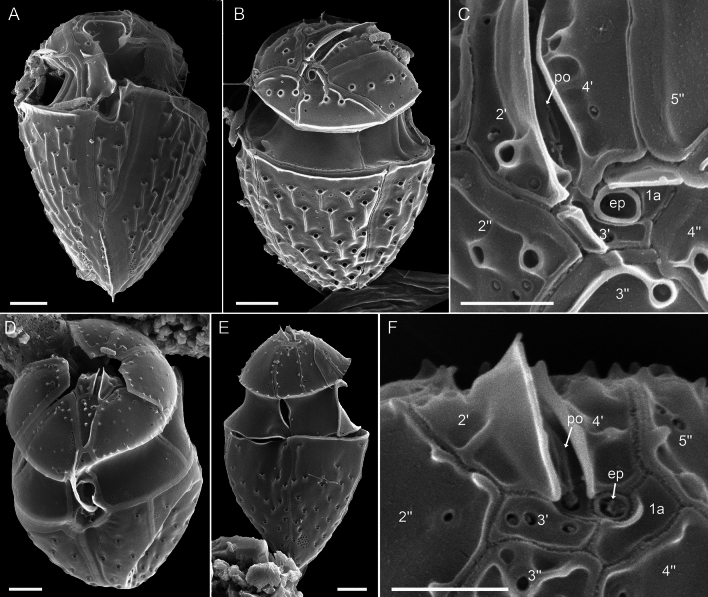
Species of *Oxytoxum*, SEM of field specimens. (**A**–**C**) *Oxytoxum laticeps*; cell in ventral (**A**) and in dorsal apical view (**B**–**C**); note that C is a higher magnification view of the cell shown in B. (**D**–**F**) *Oxytoxum* sp. 1; cell in ventral apical (**D**) and dorsal view (**E**–**F**); note that F is a higher magnification view of the cell shown in E. Plate labels according to the Kofoidean system. ep, epithecal pore. Scale bars = 2 µm (**A**–**B**, **D**–**E**) or 1 µm (**C**, **F**).

Specimens from the Pacific Ocean near Tahiti identified as *Oxytoxum gladiolus* F.Stein had the plate formula po, 4′, 1a, 6′′, 5c, 5s, 5′′′, 1′′′′ (Figs. 9A–I and 10A–F). On the epitheca, a central, apical pore plate was located between the upward bended lateral plates 2′ and 4′ (Figs. 9E‒F and 10A–F). The pore plate surrounded an elliptic apical pore, which was most obvious in internal view (Fig. 10F). On the right-lateral side and in ventral position, there was a roundish epithecal pore located between plates 4′ and 1a, which was in turn surrounded by a plate-like and ring-shaped structure (Fig. 10C, E, F). For *Oxytoxum laticeps* J.Schiller (Fig. 11A–C) and *Oxytoxum* sp. (Fig. 11D–F), an identical plate pattern was determined including the presence of a centrally located apical pore plate and a conspicuous and similar epithecal pore.

### Molecular phylogenetics

**Figure 12:**
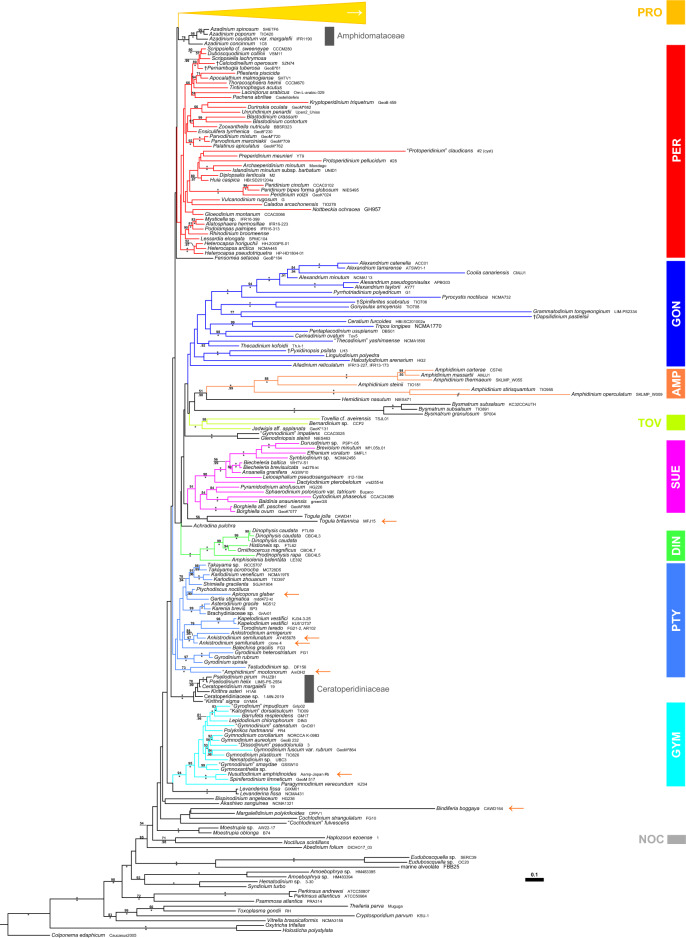
A molecular reference phylogeny recognising major groups of dinophytes. Maximum Likelihood (ML) tree of 207 systematically representative dinophyte sequences (with strain number information) inferred from a rRNA nucleotide alignment (4467 parsimony-informative positions). The numbers on the branches are ML non-parametric bootstrap (above the branch line) and Bayesian probabilities (below the branch line) for the clusters (asterisks indicate maximal support values; values under 50 for the ML bootstrap and 0.90 for Bayesian probability are not shown). Branch lengths are to scale. Orange arrows indicate taxa formerly assigned to *Amphidinium* but later identified to belong to other lineages. Abbreviations: AMP, Amphidiniales; DIN, Dinophysales; GON, Gonyaulacales; GYM, Gymnodiniales; NOC, Nocticulales; PER, Peridiniales; PRO, Prorocentrales; PTY, Ptychodiscales; SUE, †Suessiales; TOV, Tovelliales.

**Figure 13:**
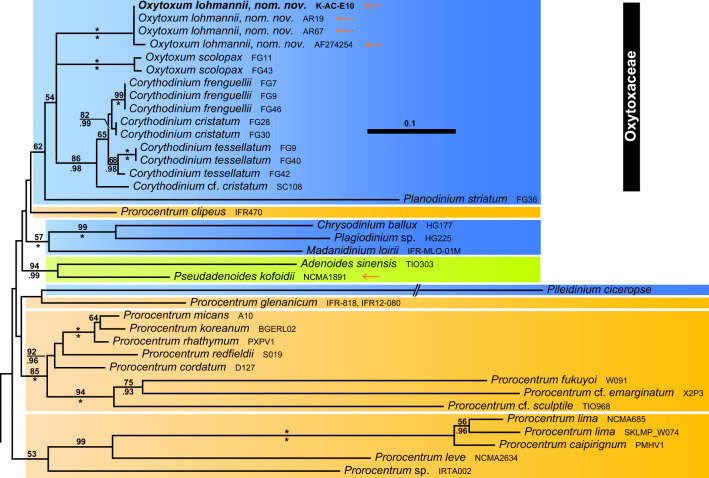
Phylogenetic sub-tree of prorocentralean dinophytes including Oxytoxaceae. Colours indicate groups with two thecal plates and an epitheca barely identifiable (brownish), a single antapical plate and five postcingular plates (bluish) and a single antapical plate and the postcingular plates split leading to the presence of posterior intercalary plates (greenish).

The rRNA (i.e., SSU + ITS + LSU) reference alignment was 1898 + 1809 + 4230 bp long and comprised 986 + 1238 + 2243 parsimony informative sites (56.3%, mean of 20.0 per terminal taxon) and 6339 distinct RAxML alignment patterns. Substitution rate heterogeneity was considerable, but tree topologies were largely congruent, irrespective of whether the Bayesian or ML algorithm was applied. Figures 12 and 13 shows the best-scoring ML tree (− ln = 246,278.86), with the internal topology not fully resolved. However, Dinophyceae were monophyletic (65LBS) and many nodes were statistically well, if not maximally, supported. A number of lineages at high taxonomic level, such as Amphidiniales (100LBS, 0.99BPP), Dinophysales (100LBS, 1.00BPP), Gonyaulacales, Gymnodiniales (94LBS, 1.00BPP), Noctilucales (71LBS, 0.98BPP), Peridiniales, Prorocentrales, Ptychodiscales, †Suessiales (91LBS) and Tovelliales (1.00BPP), as well as Amphidomataceae (78LBS, 1.00BPP) and Ceratoperidiniaceae (100LBS, 1.00BPP), were recognised. Only 21 of 206 dinophyte accessions (10.2%), scattered over the tree, were not assigned to any of those lineages. Thecate dinophytes including Amphidomataceae, Dinophysales, Gonyaulacales, Peridiniales, Prorocentrales and †Suessiales constituted a monophyletic group albeit with low support.

The monophyly of Prorocentrales (Fig. 13) was only weakly supported, and also this group internally showed strong rate heterogeneity. Prorocentrales included *Adenoides*, *Chrysodinium* F.Gómez, Y.Nakam. & Artigas, *Madanidinium* Chomérat, Oxytoxaceae, *Pileidinium* Tam. & T.Horig., *Plagiodinium* M.A.Faust & Balech, *Planodinium*, two main clusters of *Prorocentrum* Ehrenb. (including *Exuviaella* Cienk.), *Pseudadenoides* and some more species of *Prorocentrum* (i.e., *Prorocentrum clipeus* Hoppenrath and *Prorocentrum glenanicum* Nézan & Chomérat) scattered between the other lineages. Oxytoxaceae were monophyletic albeit with low statistical support (54LBS), and segregated into three highly supported lineages, namely *Corythodinium* [with the type species *Corythodinium tesselatum* (F.Stein) Loebl. & A.R.Loebl.: 86LBS, 0.98BPP], *Oxytoxum* 1 (with the type species *Oxytoxum scolopax* F.Stein: 100LBS, 1.00BPP) and *Oxytoxum* 2 (100LBS, 1.00BPP). The latter was comprised of sequences gained from dinophytes identified as *Oxytoxum lohmannii* (≡ “*Amphidinium*” *crassum*).

## Discussion

### Improved molecular phylogenetics of dinophytes

The analysis presented here attempts a balancing act between the use of rRNA sequences as long as possible and a taxon sample that is as representative as possible for the known molecular diversity of dinophytes. The result confirms the existence of the main groups already recognised^12–15^, although the statistical support, notably for deeper nodes, occasionally remains still improvable. Nevertheless, the topology of the phylogenetic tree allows new insights into the relationships of dinophytes not shown so far. For example, the recently studied Podolampaceae^36^ appear to be an integral part of the Peridiniales, and *Ailadinium* Saburova & Chomérat is probably more closely related to the Gonyaulacales, as already supposed on the basis of morphological data^37^. Such examples underline the superiority of data matrices from concatenated rRNA sequences compared to single segment analyses.

Recognising the phylogenetic relationships of unarmoured dinophytes has always been a challenge, as they are frequently poor of diagnostic traits^3^. In pre-DNA times, two major groups were distinguished^38^, namely the Gymnodiniales (with the amphiesma containing relatively numerous vesicles arranged non-serially) and the Ptychodiscales (with the pellicle strongly developed and principal structural element in the amphiesma), both of which are reflected in the present DNA-tree. The two groups may also differ in the course of the acrobase^2,3^, running in an anticlockwise direction in Gymnodiniales and being straight in Ptychodiscales.

The monophyly of the Gymnodiniales was already indicated in early molecular phylogenies^3^, and the well-supported group comprises a truly impressive diversity of utterly different life forms according to present knowledge^39,40^. However, a further main group of unarmoured dinophytes, with *Ptychodiscus* F.Stein as type of the Ptychodiscales, in the possible assemblage recognised here (including, e.g., *Asterodinium* Sournia and *Balechina* Loebl. & A.R.Loebl.^38^) has not yet been identified in DNA trees and is a decisive step towards a better understanding of dinophyte evolution. The Ptychodiscales, but not the Gymnodiniales, include also the Brachydiniaceae, confirming once more deliberate morphological concepts of the pre-DNA era^38^. The fact that these relationships have not been recognised so far^41^ is mainly due to the gain of only short sequences (e.g., *Ptychodiscus* with SSU information only), the inferior single gene analyses already mentioned and the use of taxon samples that are not representative for the known molecular diversity of dinophytes.

A third distinct lineage of unarmoured dinophytes are the Ceratoperidiniaceae with a completely circular acrobase^42,43^. The monophyly of this group was already indicated early in molecular phylogenies^44^, but they appear as independent lineage and not associated with the Ptychodiscales as previously suggested^38^. The distinctiveness from other unarmoured dinophytes may argue to recognise this group also at the rank of an order in a future classification of dinophytes (which is also true for thecate Amphidomataceae likewise not clearly assigned to any of the established taxa at order rank).

### Revised compilation of prorocentralean dinophytes

Molecular phylogenetics of Prorocentrales have always been challenging, last not least due to the strong rate heterogeneity across the constituent taxa. However, prorocentralean dinophytes are monophyletic in DNA-trees using concatenated rRNA sequences^45–48^, though rarely statistically supported. The complete set of taxa belonging to the Prorocentrales as identified in the present study has not been shown before, but there is confidence that molecular trees will improve once longer rRNA sequences than the segments such as SSU are available, particularly for the deeply diverged branches also of prorocentralean dinophytes. However, all lineages gathered in the present DNA-tree with *Prorocentrum* as type of Prorocentrales morphologically share a small through diminutive or even not recognisable epitheca that their single origin does not appear unlikely supporting the monophyly of the Prorocentrales.

Within prorocentralean dinophytes as here retrieved, three morphological types can be distinguished: (a) the cell is covered by two thecal plates, and an epitheca is barely identifiable (*Prorocentrum* incl. *Exuviaella*^47^); (b) the hypotheca exhibits (additionally to five postcingular plates) a single antapical plate only (*Chrysodinium*^49^, *Madanidinium*^50^, Oxytoxaceae^31,32^, *Pileidinium*^51^, *Plagiodinium*^52^, *Planodinium*^53–55^); (c) the hypotheca has a single antapical plate like in the type before, and the postcingular plates are split leading to the presence of posterior intercalary plates (*Adenoides*^25,56,57^, *Pseudadenoides*^25,58^). The accumulation of unusual morphological traits (i.e., only two thecal plates, posterior intercalary plates, singular antapical plate) in a single clade (i.e., prorocentralean dinophytes) is notable but not unlikely from evolutionary perspectives.

The hypotheca composed of five postcingular plates and a singular antapical plate is reminiscent of Podolampaceae^59^, Thecadiniaceae^60^ and some Protoperidiniaceae^61^, all showing only distant relationships to the prorocentralean dinophytes as identified here in the DNA-tree. Moreover, prorocentralean members with five postcingular plates share a long suture between the proximate and the distal such plates, which is morphologically unique, possibly apomorphic here but different in all other dinophytes with this hypothecal plate pattern. A posterior intercalary plate is considered abundant in Gonyaulacales, but it can be also interpreted as first of the two widespread antapical plates^48,62^. Under this assumption, gonyaulacoid dinophytes have no posterior intercalary plate(s), which are otherwise rarely if at all found in dinophytes additionally to the prorocentralean members identified here.

### Plate patterns in Oxytoxaceae

Due to the lack of cultured strains or bloom samples, detail studies of the thecal plate pattern are scarce in Oxytoxaceae^35,63–65^. There is general agreement in the number of hypothecal plates (i.e., five postcingular and a single antapical plate), five cingular plates and five plates on the epitheca. However, presence and position of an apical pore or the number of sulcal plates remains uncertain (see also a paragraph in the Supplementary information). The reference for the Kofoidean plate labelling system in fact is the position and arrangement of an apical pore plate. However, analysis of the small epithecal plates is challenging in Oxytoxaceae, especially since the apex is typically covered by helmet-like, parallel outgrowths of the lateral apical plates. Previously, epithecal plates of Oxytoxaceae are differently interpreted and are assigned either to the series of apical plates only (i.e., 5′ 0a^35^) or additionally to the series of anterior intercalary plates (i.e., 3′ 2a^63^). In *O. lohmannii*, an apical pore plate is centrally located resulting in an epithecal plate pattern of po 4′ 1a, but the exact shape of the apical pore remains elusive.

Presence, shape and arrangement of an apical pore plate have not been ultimately understood in Oxytoxaceae. In early studies^63^, an apical pore plate is not mentioned at all but in *C. tesselatum*, a very small and round “pore plate” is described and depicted^65^. It has a dorsal position very close to plate 4′′ and is separated by a very small plate 2′ and thus corresponds to the epitehcal pore observed in the present study. In contrast, a small and elongated pore plate in a central position anteriorly abutting a central hexagonal first apical plate is drawn of an undetermined species of *Oxytoxum*^64^. This position is in agreement with the apical pore plate of *O. lohmannii* and of the three *Oxytoxum* species from the Pacific Ocean, and the apical pore plate appears rather narrow and elongated (Fig. 11). It is most distinct in *O. gladiolus*, in which the raised apical pore has an oval opening and thus very much resembles the constitution typical for peridinialean dinophytes.

Despite his meticulous dissection of thecal plates, it is worthy to note that Enrique Balech (1912–2007) never observed two types of pores at the same time. Nevertheless, Oxytoxaceae are now one of those lineages, which exhibits an epithecal pore additionally to the abundant apical pore (complex) of many thecate dinophytes. The epithecal pore might even be more apparent than the apical pore plate in Oxytoxaceae that the first has been previously confused with the unrecognised latter^35^. The epithecal pore is surrounded by a plate-like structure and is thus similar to the ventral pore of the Amphidomataceae^46^, although its dorsal position is different in Oxytoxaceae. Among Prorocentrales as retrieved in the present study, *Adenoides* and *Pseudadenoides* have a plate-like pore as well in addition to the apical pore complex^57,58^, whereas others have only single, rather simple pores (*Chrysodinium*^55^, *Pileidinium*^51^, *Plagiodinium*^52^) or no pores (*Madanidinium*^50^, *Planodinium*^53^). The highly reduced epitheca of *Prorocentrum* has an accessory pore (occasionally identified as apical pore^66^), but precise functions and possible homologies must be worked out in future research.

### Nutrition in Oxytoxaceae: gorging and starving and taxonomy

With the identification of heterotrophic *O. lohmannii* as a species of Oxytoxaceae, new information of nutrition in the lineage can be provided. Oxytoxaceae are collectively considered phototrophic^38,61^, despite only limited evidence for such a general assumption. The majority of species have been described and observed based on few specimens in fixed samples, which rarely allows for the recognition of plastids. In the last comprehensive treatment of Oxytoxaceae^30^, only ten species are explicitly noted as phototrophic or having plastids. In any case, the type species of both *Corythodinium* (i.e., *C. tesselatum*) and *Oxytoxum* (i.e., *O. scolopax*) as main elements of Oxytoxaceae exhibit plastids unambiguously shown by epifluorescence microscopy^31^.

*Oxytoxum lohmannii* feeds by using a peduncle^67–69^, but such organelle has not yet been observed for any other species of Oxytoxaceae. The sparse knowledge may be due to the lack of ultrastructural studies and the small number of living organisms observed in their natural environment. In any case, there are other species of *Corythodinium* and *Oxytoxum* (e.g., *C. tesselatum*, *O. laticeps*, *Oxytoxum ovale* J.Schiller^35^), which have a round, funnel-shaped sulcal region. This shape appears very similar to the tubular area of *O. lohmannii*, from which the peduncle extrudes (Fig. 6A, B). Future research will show whether peduncle feeding (and conceivably mixotrophy for species with plastids) might be more abundant among Oxytoxaceae.

**Figure 14:**
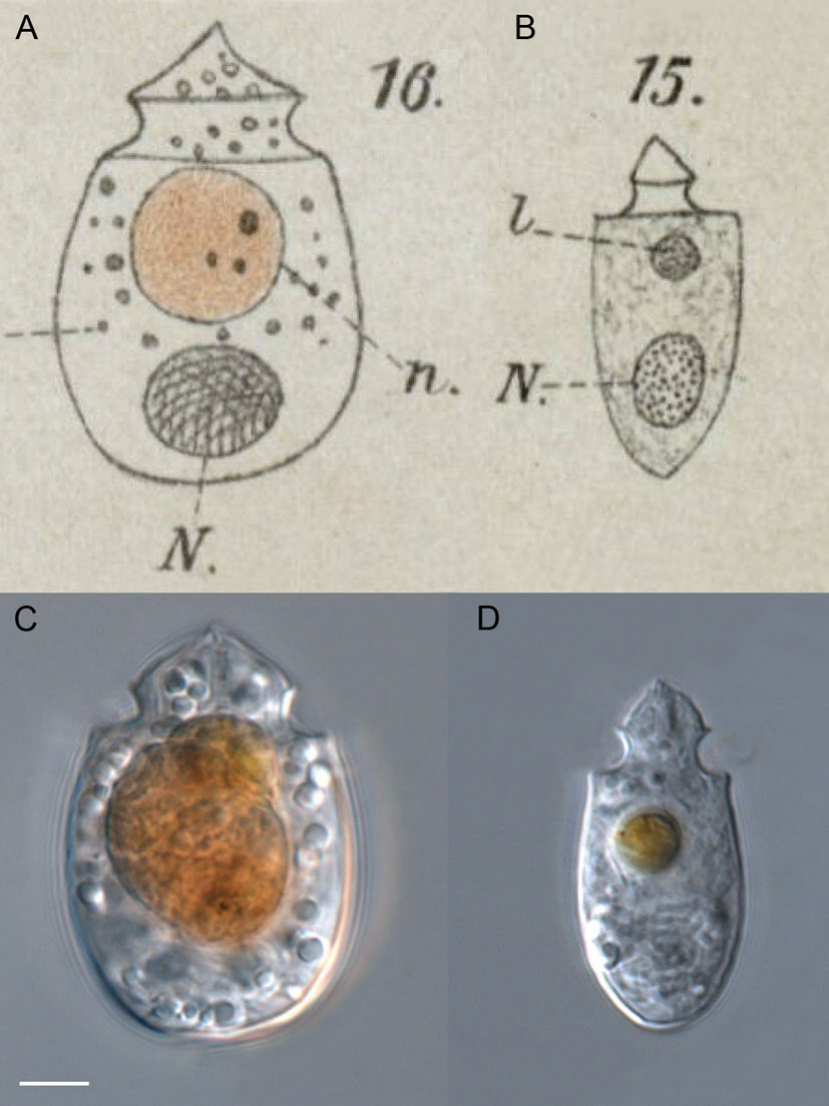
Comparing the original drawings^26^ of *Amphidinium crassum* (**A**) and *Amphidinium longum* (**B**) and cells of the clonal strain K-AC-E10 of *Oxytoxum lohmannii* (**C**–**D**). Scale bar = 5 µm (**C**–**D**). Abbreviations^26^: n, shiny yellow–brown body; N, nucleus; l, small, light-refracting body.

*Oxytoxum lohmannii* is the first species of Oxytoxaceae with demonstrated heterotrophic nutrition. In future research, the primary sources of energy must be determined for more species of the group in order to assess whether heterotrophic and phototrophic Oxytoxaceae may form phylogenetically distinct clusters, as it might be indicated already in the present DNA-tree. Notably, the cell size, shape and length/width ratio are very variable depending on the nutritional conditions in cultured clonal material of *O. lohmannii* (Figs. 1 and 2), and starving cells (corresponding to the morphology of “*A.*” *longum* with a typical length/width ratio > 2^16^) occur as well as gorged cells (corresponding to the morphology of “*A.*” *crassum* with a typical length/width ratio of 1.6^16^) (Fig. 14A–D). Both shapes have been originally described from the same locality^26^ that we think it is not too venturous to consider both names synonymous. We have unfortunately been able to establish a single and not two strains of the species from the type locality, but therefore clarify the taxonomy at least of the well-fed morph by epitypification, namely “*A.*” *crassum*.

### Epitypification of “*Amphidinium*”* crassum*

***Oxytoxum lohmannii Tillmann & Gottschling***, ***nom. nov. pro Amphidinium crassum*** **Lohmann**, Wissenschaftliche Meeresuntersuchungen. Abteilung Kiel 10: 252, 261–262, pl. XVII 16. 1908, *non Oxytoxum crassum* J.Schiller.—**Lectotype** [illustration]**, designated here**: Baltic Sea, off Germany. Schleswig–Holstein, Kiel Fjord, between Apr 1905 and Aug 1906 [non-fossil]: H. Lohmann, Wissenschaftliche Meeresuntersuchungen. Abteilung Kiel 10: pl. XVII 16!—**Epitype** [SEM stub, Figs. 5, 6 and 7]**, designated here**: Baltic Sea, off Germany, Schleswig–Holstein, Kiel Fjord (54°19.87'N, 10°9.04'E), 19 Sep 2019 [non-fossil]: U. Tillmann, M. Gottschling & H. Gu [U. Tillmann K-AC-E10] s.n. (CEDiT2023E173!). Formol-fixed material is also available (CEDiT2023RM174!). [http://phycobank.org/104223, http://phycobank.org/104225].

 = *Amphidinium longum* Lohmann, Wissenschaftliche Meeresuntersuchungen. Abteilung Kiel 10: 252, 261, pl. XVII 15. 1908, **syn. nov.**

Notes: The species has been synonymised with *Amphidinium phaeocysticola* M.Lebour^70^, whose name could be used as basionym for a new combination (ICN Art. 11.4). However, *A. phaeocysticola* is slightly larger (40 µm in cell length) than *O. lohmannii* and has a striate surface^29^. The striae are clearly visible in LM on both the epi- and hyposome, and intracellular inclusions are arranged along these striae. Such features have never been observed for *O. lohmannii* that we think the two are not conspecific. Moreover, *A. phaeocysticola* produce thin-walled coccoid cells during division, whereas *O. lohmannii* divides as motile cell by desmoschisis [also reported for *Corythodinium constrictum* (F.Stein) F.J.R.Taylor, *C. tesselatum* and *Oxytoxum sceptrum* (F.Stein) Schröd.^31^]. The general shape of more species currently affiliated with *Amphidinium* might also indicate an oxytoxacean affinity including *Amphidinium acutissimum* J.Schiller, *Amphidinium acutum* Lohmann, *Amphidinium fusiforme* G.W.Martin, *Amphidinium lanceolatum* Schröd. and *Amphidinium stigmaticum* J.Schiller, and they all deserve further study.

## Materials and methods

### Sampling, cell isolation, cultivation

A surface water sample (temperature: 15.5 °C, salinity: 17.5) and a plankton net tow sample (20 µm mesh size) was taken at Kiel Fjord (Germany) from a pier at 54°19.87′ N and 10°9.04′ E on 19th September 2019. Cells corresponding to *A. crassum*^26^ were isolated by micro-capillary pipets into 96-well plates filled with 0.2 mL filtered water from the sample site. Small amounts of the cryptophyte *Rhodomona salina* G.Karst. (strain KAC30 from the Kalmar culture collection) was added as food.

Plates were incubated at 15 °C under a photon flux density of 40 µmol m^–2^ s^–1^ on a 16:8 h light:dark photocycle in a controlled environment growth chamber (Sanyo Biomedica MIR 252; Wood Dale, USA–IL). The original strain was started from a well, in which several cells corresponding to *A. crassum* had been combined. Later, clonal substrains based on single cells from this original strain were used for all subsequent size measurement, and one clonal substrain K-AC-E10 was used for LM and SEM preparations and for DNA sequencing. All material was grown at the culture conditions described above in an natural seawater medium consisting of sterile filtered (0.2 µm VacuCap filters; Pall Life Sciences; Dreieich, Germany) and diluted North Sea water with a salinity of about 15, containing nutrients corresponding to 50% of K-medium^71^, which was slightly modified by replacing the organic phosphorous source by 3.62 µM Na_2_HPO_4_. Salinity was estimated based on electrical conductivity measurements and the practical salinity scale.

For DNA harvest, densely grown and starved material of K-AC-E10 was used, of which almost all cells of the food alga had been removed by grazing. Cells were collected by centrifugation (Eppendorf 5810R; Hamburg, Germany) in 15 mL centrifugation tubes at 3220 × g for 10 min. Cell pellets were transferred with 0.5 mL lysis buffer (SL1, provided by the NucleoSpin Soil DNA extraction Kit; Macherey–Nagel; Düren, Germany) to 1 mL microtubes and stored frozen (− 20 °C) for subsequent DNA extraction.

### Microscopy

Observation of living or fixed cells (formaldehyde: 1% final concentration, or neutral Lugol-fixed: 1% final concentration) was carried out using an inverted microscope (Axiovert 200 M; Zeiss; Munich, Germany) and a compound microscope (Axiovert 2; Zeiss), both equipped with epifluorescence and differential interference contrast optics. Light microscopic (LM) examination of thecal plates was performed on fixed cells (neutral Lugol) stained with Solophenyl Flavine 7GFE500, a fluorescent dye specific to cellulose^72^. Images were taken either with a digital camera (Axiocam MRc5; Zeiss), or videos were recorded using a digital camera (Gryphax Jenoptik; Jena, Germany) at full-HD resolution. Single frame micrographs were then extracted using Corel Video Studio software (Version X8; Coral; Ottawa, Canada). Cell length and width were measured using Axiovision software (Zeiss) and pictures taken at 1000X microscopic magnification of living cells from well-fed material and from starved cultures to cover the whole size range.

For scanning electron microscopy (SEM), cells of K-AC-E10 were collected by centrifugation (Eppendorf 5810R; 3220 × *g* for 10 min) from 15 mL of the strain. The supernatant was removed, and the cell pellet re-suspended in 60% ethanol prepared in seawater (final salinity ca 13) in a 2 mL microtube at 4 °C for 1 h in order to strip off the outer cell membrane. After centrifugation and removal of the diluted seawater supernatant, cells were fixed with formaldehyde (2% final concentration in a 60:40 mixture of deionised water and seawater) and stored at 4 °C for 3 h. Alternatively, cells were treated with TritonX (Sigma-Aldrich; St. Louis, USA–MO) at 0.2–0.5% final concentration for 1–3 h. Cells from both pre-treatment methods were collected and processed for SEM (FEI Quanta FEG 200; Eindhoven, the Netherlands) as previously described^73^.

For thecal plate pattern comparison, cells of three different species assigned to *Oxytoxum* were studied from a formalin fixed (2% final concentration) plankton sample collected at Nuku Hiva, Marquesas archipelago (Pacific Ocean) in summer 2019. For SEM observation, the sample was collected on a 3 µm polycarbonate filter and prepared as previously described^73^.

### DNA extraction, sequencing and molecular phylogenetics

Genomic DNA was extracted following the manufacturers’ instructions of the NucleoSpin Soil DNA extraction Kit (Macherey–Nagel) with an additional cell disruption step within the beat tubes; the samples were shaken for 45 s and another 30 s at a speed of 4.0 m s^−1^ in a FastPrep FP120 cell disrupter (Thermo Savant; Illkirch, France). For the elution step, 50 μL of the provided elution buffer were spun through the spin column, and elution was subsequently repeated with another 50 μL to increase the DNA yield, leading to a total elution volume of 100 μL. Various regions of rRNA segments, including SSU, ITS and LSU, were amplified using the following primer sets: 1F (5′ −AAC CTG GTT GAT CCT GCC AGT − 3′) and 1528R (5′−TGA TCC TTC TGC AGG TTC ACC TAC−3′) for the SSU; ITS1 (5´− TCC GTA GGT GAA CCT GCG G − 3´) and ITS4 (5´− TCC TCC GCT TAT TGA TAT GC− 3´) for ITS; DirF (5´−ACC CGC TGA ATT TAA GCA TA− 3´) and D2CR (5´−CCT TGG TCC GTG TTT CAA GA−3´) for LSU. Conditions of the PCR for the respective region, amplicon check and purification, as well as the sequencing process, followed the protocols previously described^74,75^.

To compute a dinophyte reference tree inferred from a concatenated rRNA alignment^76^, a systematically representative set comprising 206 dinophytes (plus 17 outgroup accessions; Table S1) was compiled. For alignment constitution, separate matrices of the rRNA operon were constructed, aligned using ‘MAFFT’ v6.502a^77^ and concatenated afterwards. Phylogenetic analyses were carried out using Maximum Likelihood (ML) and Bayesian approaches, as described previously^76^. Briefly, the Bayesian analysis was performed using ‘MrBayes’ v3.2.7a^78^ (freely available at http://mrbayes.sourceforge.net/download.php) under the GTR + Γ substitution model and the random-addition-sequence method with 10 replicates. Two independent analyses of four chains (one cold and three heated) with 20,000,000 generations were run, sampled every 1000th cycle, with an appropriate burn-in (10%) inferred from evaluation of the trace files using Tracer v1.7.166. For the ML calculations, the MPI version of ‘RAxML’ v8.2.4^79^ (freely available at http://www.exelixislab.org/) was applied using the GTR + Γ substitution model under the CAT approximation. The best-scoring ML tree was determined, and 1000 non-parametric bootstrap replicates (rapid analysis) were performed in a single step. Statistical support values (LBS: ML bootstrap support; BPP: Bayesian posterior probabilities) were drawn on the resulting, best-scoring tree.

### Supplementary Information

Supplementary Video S1.

Supplementary Information 1.

## Data Availability

The sequence data generated during the current study are available in the GenBank repository (https:// www.ncbi.nlm.nih.gov/nuccore). For corresponding accessions numbers, one may refer to the extensive voucher list (Table S1) in the Supplementary Information.
