# Supplementary material for: Oxytoxaceae are prorocentralean rather than peridinialean dinophytes and taxonomic clarification of heterotrophic Oxytoxum lohmannii (≡ “Amphidinium” crassum) by epitypification
Source: Sci Rep. 2024 Mar 20;14:6689. doi: 10.1038/s41598-024-56848-y (PMC10954643; doi:10.1038/s41598-024-56848-y)
Supplement: Supplementary file 2 — Supplementary Information 1. [file 41598_2024_56848_MOESM2_ESM.pdf]

# Oxytoxaceae are prorocentralean rather than peridinialean dinophytes and taxonomic clarification of heterotrophic *Oxytoxum lohmannii* (≡ “*Amphidinium*” *crassum*) by epitypification

Marc Gottschling, Stephan Wietkamp, Alexis Bantle & Urban Tillmann

**Supplementary information**

**Supplementary Notes**

Both *A. crassum* and *A. longum* show a wide distribution in the Baltic Sea[1,2], the Irish Sea[3], the Black Sea[4], the Arctic Ocean off Svalbard[5] and the Mediterranean Sea[6-8]. Additionally, *A. crassum* is present in the North Sea and the English Channel[9,10] and the western Norther Atlantic[11-13] and A. longum in the Arctic Ocean off Canada[14], the Barents Sea[15] and the Norwegian Sea[16]. *Amphidinium crassum* has been studied regarding food uptake[17,18] and was used as target species for studying lytic activity of allelochemicals[19,20]. A strain determined as *A. longum* was established by Suzanne Strom, which was used several times in protist grazing studies[21-23] and to work out lipid composition in heterotrophic species [24].

The variously reduced epitheca of Prorocentrales is reminiscent of the morphology of Amphidiniales, which explains why some of their constituent species have been initially considered members of *Amphidinium*. Amphidiniales are clearly monophyletic, but are nesting within thecate dinophytes. This is a blemish and implausible part of the presented DNA-tree, as Amphidiniales are a deeply diverged branch of the dinophyte Tree of Life[25-27]. However, the long branches likewise present in Gonyaulacales and *Bysmatrum* M.A.Faust & Steid. may account for perturbation of the phylogenetic signal for the taxon sample used here. In any case, the monophyly of Amphidiniales including the type species *A. operculatum* allows to allocate those species described under but are not closely related to *Amphidinium*, including *"A." crassum* studied here in detail.

With the cell body composed of two large cellulosic plates, the close relationship between dinophysoid and prorocentroid dinophytes has been suggested[28,29]. The support for this assumption is weaker than for many other nodes in NGS phylogenies (with a very limited taxon sample[27,30]), but a corresponding topology has been never shown in rRNA trees as the present one with a much broader taxon sample. Therefore, the similar morphology found in Dinophysales and Prorocentrales might not be result of a shared but independent evolution.

Four or five sulcal plates are reported for Oxytoxaceae[31-34]. For *O. lohmannii*, the sulcal plates forming the caved and difficult-to-see funnel-shaped emergence site of the peduncle are difficult to observe in SEM or fluorescence microscopy. The posterior sulcal plate is very prominent and usually has a round indentation (also very characteristically present in other species of *Corythodinum* and *Oxytoxum*[34]), from which the flagella arise. The arrangement and shape of other sulcal plates around the peduncle tube with two very slender lateral plates sd and ss, and a more triangular sa extending into the epitheca, have to be considered as preliminary until further observations. For *O. gladiolus*, a fifth median sulcal plate in central position is clearly discernible (Fig. 9I) and might thus also be present in other Oxytoxaceae.

## Tables

**Table S1: Voucher list**. All names are given under the rules of the ICN, with the author standard forms[35]. Abbreviation: n.inf., no information. If 'holotype' or 'epitype' is noted for a species name, then it refers to material, from which the type was prepared.

| Species name with author | Strain No. | Locality | Date | Collector(s) [isolator] | GenBankNo(s) | Reference |
|---|---|---|---|---|---|---|
| <u>outgroup</u> | | | | | | |
| *Amoebophrya* sp. [isolated from *Levanderina fissa* (Levander) Moestrup, Hakanen, Gert Hansen, Daugbjerg & M.Ellegaard] | n.inf. | western North Atlantic, Chesapeake Bay, off USA–MD | n.inf. | n.inf. | HM483394 (SSU+ITS+LSU) | 36 |
| *Amoebophrya* sp. [isolated from *Akashiwo sanguinea* (K.Hirasaka) Gert Hansen & Moestrup] | n.inf. | western North Atlantic, Chesapeake Bay, off USA–MD | n.inf. | n.inf. | HM483395 (SSU+ITS+LSU) | 36 |
| *Colponema edaphicum* Mylnikov & Tikhonenkov, 2005 (holotype) | Caucasus2005 | Russia: Krasnodar, Vorontsovskaya (43°37'N, 39°56'E) | Jan 29, 2005 | A. Mylnikov 536 | KF651064 (rRNA) | 37 |
| *Cryptosporidium parvum* Tyzzer, 1907 (isolated from *Bos taurus* Linnaeus, 1758) | KSU-1 | US–KS | Mar, 1987 | n.inf. | AF040725 (rRNA) | 38 |
| *Euduboscquella* sp. [isolated from *Favella arcuata* (K.Brandt, 1906)] | OC20 | westerm North Atantic, off USA–MD, Assawoman Bay: Ocean City (38°20'N, 75°06W) | Aug 11, 2010 | n.inf. | JN934989 (SSU+ITS+LSU) | 39 |

| | | | | | | |
|---|---|---|---|---|---|---|
| *Euduboscquella* sp. (isolated from *Tintinnopsis* cf. *subacuta* Jørg., 1899) | SERC39 | USA–MD: Rhode River (38°53'N, 76°33W) | Oct 8, 2009 | n.inf. | JN934992 (SSU+ITS+LSU) | 39 |
| *Hematodinium* sp. (isolated from *Callinectes sapidus* Rathbun, 1896) | 3-30 | western North Atlantic, Gulf of Mexico, off USA–MS | n.inf. | N. Zimmerman & J. Lotz s.n. | JQ815886 (SSU+ITS+LSU) | 40 |
| *Holosticha polystylata* Borror & B.J.Wicklow, 1983 | n.inf. | USA–CO: Boulder, University of Colorado campus | n.inf. | n.inf. | AF508760 (SSU+ITS+LSU) | 41 |
| marine alveolate | FBB25 | USA–MA: Boston, Blanes Bay Microbial Observatory | n.inf. | n.inf. | EU304548 (rRNA) | 42 |
| *Oxytricha trifallax* Greslin, D.M.Prescott, Y.Oka, Loukin & Chappell, 1989 | n.inf. | n.inf. | n.inf. | n.inf. | FJ545743 (rRNA) | 43 |
| *Perkinsus andrewsi* Coss, J.Robledo, G.Ruiz & Vasta, 2001 [isolated from *Macoma balthica* (Linnaeus, 1758)] | ATCC50807 (≡ PAND-A8–4a) | USA–MD: Edgewater, Rhode River, Fox Point (38°51'N, 76°32'W) | Aug, 1996 | C. Coss s.n. | AY305326 (rRNA) | 44 |
| *Perkinsus atlanticus* C.Azevedo, 1989 [isolated from *Ruditapes decussatus* (Linnaeus, 1758)] | ATCC50984 (≡ ALG1) | eastern North Atlantic, off Portugal: Algarve, Ria Formosa | Aug 9, 2002 | n.inf. | AF509333 (rRNA) | 45 |
| *Psammosa atlantica* N.Okamoto, A.Horák & P.J.Keeling, 2012 | PRA314 | western North Atlantic, off Canada, Bay of Fundy: Nova Scotia, | Jul 30, 2008 | N. Okamoto s.n. | JN873310 (SSU) | 46 |

| | | | | | | |
|---|---|---|---|---|---|---|
| | | Blomidon Beach (45°15'N, 64°21'W) | | | | |
| *Syndinium turbo* Chatton, 1910 [isolated from *Paracalanus parvus* (C.Claus, 1863)] | n.inf. | westren Mediterranean Sea, off Spain: Barcelona, Port Olímpic | Aug 13, 2003 | n.inf. | DQ146405 (SSU+ITS) | 47 |
| *Theileria parva* (Theiler, 1904) (isolated from *Bos taurus* Linnaeus, 1758) | Muguga | Kenya | n.inf. | n.inf. | NC_007344 (AAGK01000001) | 48 |
| *Toxoplasma gondii* (Nicolle & Manceaux, 1908) (isolated from *Homo sapiens* Linnaeus, 1758) | RH | n.inf. | n.inf. | n.inf. | X75429 (rRNA) | Ding et al. (unpubl. 1993) |
| *Vitrella brassicaformis* Oborník, D.Modrý, M.Lukeš, Cernotíková-Stříbrná, Cihlář, Tesařová, Kotabová, Vancová, Prášil & J.Lukeš (isolated from an anthozoan species) | NCMA3155 (≡ RM11) | western South Pacific, off Australia, Great Barrier Reef (23°30'S, 152°00'E) | Jan 12, 2001 | K. Miller & C. Mundy [R.A. Andersen & R. Moore] s.n. | HM245049 (rRNA) | 49 |
| <u>unplaced dinophytes</u> | | | | | | |
| *Abedinium folium* E.C.Cooney, N.Okamoto & P.J.Keeling (holotype) | DICHO17_03 | eastern North Pacific, off USA–CA, Monterey Bay (–600m) | Sep 7, 2017 | N. Okamoto [Rachel Carson] s.n. | MT191358 (SSU+ITS+LSU) | 50 |
| *Achradina pulchra* Lohmann | n.inf. | western South Atlantic, off Brazil, São Sebastião | Apr 22, 2013 | F. Gómez s.n. | MF543363 (SSU) | 51 |

| | | | | | | |
|---|---|---|---|---|---|---|
| | | Channel (23°50'S, 45°24'W, –40m) | | | | |
| *Akashiwo sanguinea* (K.Hirasaka) Gert Hansen & Moestrup | NCMA1321 (≡ CCCM840, GSBL, UTEX2263) | off USA–NY, Great South Bay (40°23'N, 73°09'W) | Jul, 1958 | I. Pintner s.n. | AY831412 (rRNA) | 52,53 |
| *Azadinium caudatum* var. *margalefii* (Rampi) Nézan & Chomérat | IFR1190 [IFR1140, IFR10-388] | eastern North Atlantic, Celtic Sea, off France: Brittany, Finistère, Concarneau (47°50'N, 3°57'W) | Dec 15, 2009 | [E. Nézan] s.n. | JQ247707 (SSU), JQ247704 (ITS), JQ247708 (LSU) | 54 |
| *Azadinium concinnum* Tillmann & Nézan (holotype) | 1C6 (single and multiple cells, LSU clone 4) | North Atlantic, Irminger Sea, off Greenland (62°14'N, 37°27'W) | Aug, 2012 | U. Tillmann [Maria S. Merian] [U. Tillmann] s.n. | KJ481826 (SSU), KJ481827 (ITS), KJ481831 (LSU) | 55 |
| *Azadinium poporum* Tillmann & Elbr. | TIO420 | Mediterranean Sea, Ionian Sea, Ambracian Gulf, off Greece: Aetolia-Acarnania (38°55'N, 21°06'E) | Sep 27, 2014 | n.inf. | MH685461 (SSU), MH685498 (ITS), MH685480 (LSU) | 56 |
| *Azadinium spinosum* Elbr. & Tillmann | SHETF6 | North Sea, off Shetland Islands (60°13'N, 1°00'W) | May, 2011 | U. Tillmann [Heincke] [U. Tillmann] s.n. | JX559885 (SSU+ITS+LSU) | 57 |
| *Bindiferia boggaya* (Sh.Murray & D.J.Patt.) Sh.Murray & Hoppenrath | CAWD164 | western South Pacific, Polynesia, off New Zealand: Northland, Aupōuri, Rangaunu Harbour (34°58'S, 173°17'E) | Mar, 2009 | [L. Rhodes] s.n. | MW720768 (SSU), MW722976 (ITS), MW722979 (LSU), MW720712 (LSUd8d10) | Stuart et al. (unpubl. 2020), 58 |

| | | | | | | |
|---|---|---|---|---|---|---|
| *Bysmatrum granulosum* Ten-Hage, Quod, J.Turquet & Couté | SP004 | western North Pacific, Gulf of Thailand, off Malaysia: Perhentian (5°53'N, 102°45'E) | May 17, 2017 | n.inf. | MG826113 (SSU), MG826365 (ITS), MG826105 (LSU) | 59 |
| *Bysmatrum subsalsum* (Ostenf.) M.A.Faust & Steid. | KC32CCAUTH | off Greece, North Aegean Sea: Thessaloniki, Porto-Lagos (40°58'N, 25°07'E) | n.nf. | N. Nikolaidis s.n. | HQ845326 (SSU+ITS+LSU) | 60 |
| *Bysmatrum subsalsum* (Ostenf.) M.A.Faust & Steid. | TIO891 | China: Hainan | n.inf. | n.inf. | MK012074 (SSU), MK012084 (ITS), MK012079 (LSU) | 61 |
| Ceratoperidiniaceae sp. | 1-MN-2019 | western North Pacific, off Japan, Funka Bay: Hokkaidō, Oshima (42°09'N, 140°41'E) | Aug 31, 2018 | M. Natsuike s.n. | LC537274 (LSU) | Natsuike et al. (unpubl. 2020) |
| *Ceratoperidinium margalefii* A.R.Loebl. | 19 | western Mediterranean Sea, off Spain: Catalonia, Girona, Alt Empordà, Empuriabrava (42°14'N, 3°08'E) | Jul, 2011 | n.inf. | KF245455 (LSU) | 62 |
| “*Cochlodinium*” *fulvescens* M.Iwataki, H.Kawami & Matsuoka | n.inf. | eastern North Pacific, off USA–CA, Santa Monica Bay: Los Angeles (33°54'N, 118°28'W) | Dec 4, 2006 | n.inf. | HQ896315 (SSU), AB295051 (LSU) | 63,64 |
| *Cochlodinium strangulatum* F.Schütt | FG10 | western South Atlantic, off Brazil, São Sebastião Channel (23°50'S, 45°24'W) | Aug 23, 2013 | n.inf. | KY468923 (LSU) | 65 |

| | | | | | | |
|---|---|---|---|---|---|---|
| *Fensomea setacea* Tillmann & Gottschling (holotype) | GeoB*184 | western South Atlantic (31°25'S, 37°31'W, –5m) | Mar 7, 2000 | K.J.S. Meier & M. Streng [Meteor 46/4] [M. Kirsch] WP 3/7/a | MW267274 (SSU+ITS+LSU), MW267282 (LSUd8d10) | 66 |
| *Glenodiniopsis steinii* Wołosz. (reference material) | NIES463 (≡ TM3D6) | Japan: Iwate, Shizukuishi | Sep 10, 1984 | [T. Sawaguchi] s.n. | AF274257 (SSU), EF058255 (LSU) | 67,68 |
| “*Gymnodinium*” *impatiens* Skuja | CCAC0025 (≡ M0925) | Germany: Brandenburg, Neuglobsow | 1992 | [B. Marin] s.n. | EF058239 (SSU), EF058259 (LSU) | 68 |
| *Haplozoon ezoense* Wakeman, A.Yamag. & T.Horig. (isolated from *Praxillella affinis* subsp. *pacifica* Berkeley, 1929) (reference material) | 1 | western North Pacific, Sea of Japan, off Japan: Hokkaidō, Shiribeshi, Otaru, Oshoro (43°13'N, 140°53'E) | 2016 | n.inf. | MH118544 (SSU), MH118546 (LSU) | 69 |
| *Hemidinium nasutum* F.Stein (reference material) | NIES471 (≡ 87SPD1) | Japan: Honshū, Ibaraki, Tsuchiura | Aug 27, 1987 | [T. Sawaguchi s.n.] | AY443016 (SSU), EF058260 (LSU) | 68,70 |
| *Kirithra asteri* Boutrup, Tillmann, Daugbjerg & Moestrup (holotype) | H1A6 | western South Atlantic, off Argentina (41°11'S, 57°52'W, –5m) | Sep 9, 2015 | U. Tillmann [Houssay] [U. Tillmann] s.n. | MW267281 (SSU), MW267275 (ITS), MF666674 (LSU) | 66,71 |
| “*Kirithra*” *sigma* Zhangxi Hu, Zhun Li, H.H.Shin & Y.Z.Tang | GYM04 | western North Pacific, Yellow Sea, off China, Jiaozhou Bay: Shandong, Qingdao (36°01'N, 120°22'E, –25m) | Aug 17, 2017 | Z. Hu s.n. | MW874836 (SSU), MW874968 (LSU) | 72 |
| *Levanderina fissa* (Levander) Moestrup, Hakanen, Gert | GIXM01 | western North Pacific, East China Sea, off China: Fujian, Xiamen | 2004 | n.inf. | DQ084522 (SSU), DQ084523 (ITS), DQ084521 (LSU) | Hou et al. (unpubl. 2005) |

| | | | | | | |
|---|---|---|---|---|---|---|
| Hansen, Daugbjerg & M.Ellegaard | | | | | | |
| *Levanderina fissa* (Levander) Moestrup, Hakanen, Gert Hansen, Daugbjerg & M.Ellegaard | NCMA431 (≡ CCCM796, LISBOA176) | Portugal: near Santiago do Cacem, Santo Andre lagoon (38°03'N, 8°48'W) | Nov 1, 1980 | E. Silva s.n. | AY443015 (SSU), JQ972685 (ITS), EF205007 (LSU) | 70,73,74 |
| *Margalefidinium polykrikoides* (Margalef) F.Gómez, Richlen & D.M.Anderson | CPPV1 | eastern North Pacific, Gulf of California, off Mexico: Baja California, Bahía de La Paz | 2000 | [L. Morquecho] s.n. | JQ616826 (SSU+ITS+LSU), JQ616831 (LSU) | 63,75 |
| *Moestrupia oblonga* (J.Larsen & D.J.Patt.) Gert Hansen & Daugbjerg | B74 | western North Pacific, off Japan: Okinawa, Kunigami, Motobu (26°43'N, 127°52'E) | Sep 1, 2009 | S. Suda s.n. | LC025879 (SSU), LC025898 (ITS), LC025917 (LSU) | 76 |
| *Moestrupia* sp. | AW22-17 | western North Pacific, off Japan: Okinawa, Awase (26°19'N, 127°50'E) | Apr 22, 2013 | S. Suda s.n. | LC025893 (SSU), LC025912 (ITS), LC025931 (LSU) | 76 |
| *Noctiluca scintillans* (Macartney) Kof. & Swezy | n.inf. | western North Pacific, South China Sea, off China, Clear Water Bay: Hong Kong (22°20'N, 114°16'E) | Mar 26, 2006 | J.-S. Ki s.n. | GQ380592 (SSU+ITS+LSU) | 77 |
| *Pseliodinium helix* (C.H.G.Pouchet) F.Gómez | LIMS-PS-2554 | n.inf. | n.inf. | n.inf. | MH465611 (SSU), MH465610 (LSU) | Shin & Li (unpubl. 2018) |

| | | | | | | |
|---|---|---|---|---|---|---|
| *Pseliodinium pirum* (F.Schütt) F.Gómez | PHJZB1 | western North Pacific, Yellow Sea, off China, Jiaozhou Bay: Shandong, Qingdao (36°06'N, 120°15'E) | Aug 11, 2015 | n.inf. | MH469533 (SSU), MH469535 (LSU) | 78 |
| *Togula britannica* (Herdman) M.F.Jørg., Sh.Murray & Daugbjerg | MFJ15 (≡ NORCCA K-0658) | North Sea, off Denmark, Ise Fjord | 2001 | M.F. Jørgensen s.n. | AY455679 (LSU) | 79 |
| *Togula jolla* M.F.Jørg., Sh.Murray & Daugbjerg | CAWD41 | western South Pacific, Tasman Sea, New Zealand: Awaroa (40°52'S, 172°59'E) | Feb, 1997 | [L. Rhodes] s.n. | MW720769 (SSU), MW722977 (ITS), MW722980 (LSU) | 58 |
| <u>Tovelliales</u> | | | | | | |
| *Esoptrodinium* sp. | CCP2 | USA–NC: Pond near Cashiers (35°08'N, 83°05'W) | Jul, 2009 | n.inf. | JQ439940 (rRNA) | 80 |
| *Jadwigia* aff. *applanata* Moestrup, K.Lindb. & Daugbjerg | GeoK*131 | Germany: Berlin, Köpenick, Teufelsseemoor | May 28, 2023 | M. Gottschling & B. Zierach [M. Gottschling] D234 | OR943603 (SSU+ITS+LSU), OR943598 (LSUd8d10) | this study |
| *Tovellia* cf. *aveirensis* Pandeir., Craveiro, Daugbjerg, Moestrup & Calado | TSJL01 | China: Fujian, Jiulong Jiang, Xipi reservoir (25°08'N, 117°31'E) | Apr 28, 2013 | n.inf. | KU359052 (SSU), KU359051 (ITS), KU359050 (LSU) | 81 |
| <u>Amphidiniales</u> | | | | | | |
| *Amphidinium carterae* Hulburt | CS740 (≡ SM01) | western South Pacific, Tasman Sea, off | n.inf. | n.inf. | JQ647424 (ITS), AY460578 (LSU) | 82 |

| | | | | | | |
|---|---|---|---|---|---|---|
| | | Australia, Botany Bay: NWS, Sydney | | | | |
| *Amphidinium massartii* Biecheler | AMJJ1 | western North Pacific, East China Sea, off South Korea: Jeju, Namwon (33°03'N, 126°07'E, −2m) | Oct, 2011 | n.inf. | HF674441 (SSU+ITS+LSU) | 83 |
| *Amphidinium operculatum* Clap. & J.Lachm. | SKLMP_W009 | western North Pacific, South China Sea, off China: Hong Kong | n.inf. | n.inf. | MK583709 (SSU), MK590185 (ITS), MK605122 (LSU) | Lam et al. (unpubl. 2019), Yiu et al. (unpubl. 2019) |
| *Amphidinium steinii* (Lemmerm.) Kof. & Swezy | TIO181 | western North Pacific, South China Sea, off China: Guangxi, Beihai, Weizhou (21°02'N, 109°08'E) | Jun 6, 2015 | n.inf. | MZ359142 (ITS), MZ351950 (LSU) | 84 |
| *Amphidinium stirisquamtum* Z.Luo, Na Wang & H.Gu | TIO955 | western North Pacific, East China Sea, off China: Fujian, Pingtan (25°27'N, 119°47'E) | Apr 16, 2019 | n.inf. | MZ663992 (SSU), MZ663990 (ITS), MZ668341 (LSU) | 85 |
| *Amphidinium thermaeum* Dolapsakis & Econ.-Amilli | SKLMP_W055 | South China Sea | n.inf. | n.inf. | MK583719 (SSU), MK605132 (LSU), MK544125 (LSUd8d10) | Yiu et al. (unpubl. 2019) |
| <u>Gymnodiniales</u> | | | | | | |
| *Barrufeta resplendens* (Hulburt) H.Gu, Z.Luo & K.N.Mert. | GM17 | western North Atlantic, Gulf of Mexico, off USA–LA (28°52'N, 90°29'W) | Jul 29, 2014 | n.inf. | KY688183 (SSU), KT203384 (ITS), KT203382 (LSU) | 86,87 |

| | | | | | | |
|---|---|---|---|---|---|---|
| *Bispinodinium angelaceum* N.Yam. & T.Horig. (holotype) | HG236 | western North Pacific, off Japan: Kyūshū, Kagoshima, off Mageshima (30°41'N, 130°50'E) | May 15, 2008 | R. Terada s.n. | AB762397 (SSU), AB762398 (LSU) | 88 |
| "*Dissodinium*" *pseudolunula* Swift ex Elbr. & Drebes | JHW0205-1 | eastern Indian Ocean, off South Korea | n.inf. | n.inf. | AY526523 (LSU) | 89 |
| *Gymnodinium aureolum* (Hulburt) Gert Hansen | GeoB 232 | Mediterranean Sea, Ionian Sea, off Italy: Gulf of Taranto (40°07'N, 17°19'E) | Oct 26, 2002 | D. Saracino [M. Kirsch] s.n. | KJ481834 (SSU+ITS+LSU) | 55 |
| "*Gymnodinium*" *catenatum* H.W.Graham | GnCt01 | eastern Indian Ocean, East China See, off South Korea: Nanpo, Jinhae Bay | n.inf. | n.inf. | DQ785882 (rRNA) | 90 |
| *Gymnodinium corollarium* Sundström, A.Kremp & Daugbjerg (holotype) | NORCCA K-0983 (≡ GCTV-B4) | Baltic Sea, off Finland: S of Åland Islands (58°53'N, 20°19'E) | Mar, 2005 | [A. Kremp] s.n. | MN416309 (SSU), MN422069 (ITS), FJ211386 (LSU) | 91,92 |
| *Gymnodinium fuscum* var. *rubrum* Baumeister ex Romeikat, Knechtel & Gottschling (epitype) | GeoM*864 (≡ CCAC9044B, CCAP1117/10) | Germany: Bavaria, Traunstein, Seeon, peat bog near Brunnen-See (47°59'N, 12°26'E, 536m) | Jun 28, 2017 | C. Romeikat, M. Gottschling & H. Reich [C. Romeikat] D099 | MK405489 (SSU+ITS+LSU) | 93 |
| *Gymnodinium plasticum* Na Wang, Z.Luo, K.N.Mert., F.M.G.McCarthy & H.Gu (holotype) | TIO826 | Canada: Ontario, Plastic Lake (45°18'N, 79°23'E) | n.inf. | n.inf. | KY688188 (SSU), KY688186 (ITS), KY688184 (LSU) | 87 |

| | | | | | | |
|---|---|---|---|---|---|---|
| “*Gymnodinium*” *smaydae* N.S.Kang, H.J.Jeong & Moestrup (holotype) | GSSW10 | western North Pacific, Yellow Sea, off South Korea, Shiwha Bay (37°18’N, 126°36’E) | May, 2010 | n.inf. | HG005135 (SSU+ITS+LSU) | 94 |
| *Gymnoxanthella* sp. (isolated from *Spongotrochus glacialis* Popofsky, 1908) | n.inf. | North Pacific | fall, 2010 | n.inf. | AB860180 (rRNA) | 95 |
| “*Gyrodinium*” *impudicum* S.Fraga & I.Bravo | GrIp02 | eastern Indian Ocean, off South Korea: Hase | n.inf. | n.inf. | DQ779993 (rRNA) | 53 |
| “*Katodinium*” *dorsalisulcum* E.M.Hulbert, J.A.McLaughlin & Zahl (isolated from coral) | TIO09 | western North Pacific, South China Sea, off China: Hainan, Sanya (18°14'N, 109°28'E) | Nov 16, 2014 | n.inf. | MH732689 (SSU), MH732674 (ITS), MH732682 (LSU) | 96 |
| *Lepidodinium chlorophorum* (Elbr. & Schnepf) Gert Hansen, Botes & de Salas | DIN3 | North Sea, off France: Lower Normandy, Calvados, Douvres-la-Délivrande, Luc-sur-Mer | 1995 | J. Fresnel | AY331681 (SSU+ITS+LSU) | Grzebyk et al. (unpubl. 2011) |
| *Nematodinium* sp. | UBC3 (chimeric) | eastern North Pacific, off Canada: British Columbia, Vancouver Island (48°50'N, 125°08'W) | April, 2006; April 28, 2007 | Anonymous BSL-2009a | FJ947038 (SSU), FJ947041 (LSU) | 97 |
| *Nusuttodinium amphidinoides* (Geitler) Y.Takano & T.Horig. (reference material) | Aamp-Japan #b | Japan: Hokkaidō, Sapporo (43°04'N, 141°21'W) | Apr 9, 2003 | Y. Takano s.n. | AB921307 (rRNA) | 98 |

| | | | | | | |
|---|---|---|---|---|---|---|
| *Paragymnodinium verecundum* K.Yokouchi & T.Horig. | KZ04 | western North Pacific, off Japan: Miyagi, Kitsunezaki (38°21'N, 141°25'E) | Mar 26, 2018 | K. Yokouchi s.n. | LC575960 (SSU), LC575961 (LSU) | 99 |
| *Polykrikos hartmannii* W.Zimm. | FR4 | USA–NY: Forge River (40°49'N, 72°50'W) | July 23, 2007 | Y. Tang s.n. | KC814183 (SSU), HQ834210 (LSU) | 100 |
| *Spiniferodinium limneticum* (Wołosz.) Kretschmann & Gottschling | GeoM 517 (≡ CCAC5092B, CCBA AA-276) | Poland: Lesser Poland, Tatra, Zakopane (49°17'N, 19°57'E) | Sep 11, 2012 | M. Gottschling, C. Zinßmeister, N.H. Filipowicz & P.M. Owsianny [J. Kretschmann P6] PL002 | KR362900 (SSU+ITS+LSU) | 101 |
| <u>Ptychodiscales</u> | | | | | | |
| “*Amphidinium*” *mootonorum* Sh.Murray & D.J.Patt. | AmDH2 | western North Pacific, Yellow Sea, off South Korea: N Jeolla, Gochang, Dongho | Dec 24, 2012 | n.inf. | KT371435 (LSU) | 102 |
| *Ankistrodinium armigerum* K.Watanabe, Miyoshi, F.Kubo, Sh.Murray & T.Horig. | n.inf. | western North Pacific, Sea of Japan, off Japan: Hokkaidō, Ishikari Beach (43°15'N, 141°21'E) | n.inf. | n.inf. | AB858349 (SSU), AB858350 (LSU) | 103 |
| *Ankistrodinium semilunatum* (Herdman) Hoppenrath, | clone 4 | eastern North Pacific, Boundary Bay, off USA–WA | summer, 2006 | n.inf. | JQ179860 (SSU), JQ179863 (LSU) | 104 |

| | | | | | | |
|---|---|---|---|---|---|---|
| Sh.Murray, Sparmann & B.S.Leander | | | | | | |
| *Ankistrodinium semilunatum* (Herdman) Hoppenrath, Sh.Murray, Sparmann & B.S.Leander | n.inf. | n.inf. | n.inf. | n.inf. | AY455678 (LSU) | 105 |
| *Apicoporus glaber* (Hoppenrath & Okolodkov) Sparmann, B.S.Leander & Hoppenrath | n.inf. (chimeric) | North Sea, off Germany: Schleswig-Holstein, Sylt | Mar, 2009 | n.inf. | EU293235 (SSU), JQ179867 (LSU) | 104,106 |
| *Asterodinium gracile* Sournia | NG512 | western North Pacific, East China Sea, off Japan: Kyūshū, Nagasaki, Mikuriyachō-satomen (33°22'N, 129°40'E) | Oct, 2017 | n.inf. | LC438754 (ITS+LSU) | 107 |
| *Balechina gracilis* (Bergh) F.Gómez, Artigas & Gast | FG3 | eastern North Atlantic, Celtic Sea, English Channel (48°34'N, 4°05'W) | Jul 27, 2018 | F. Gómez [Antea] s.n. | MW077541 (SSU+ITS+LSU) | 108 |
| Brachydiniaceae sp. | GrAr01 | western North Pacific, Sea of Japan, off South Korea: Chilchondo | n.inf. | n.inf. | DQ779991 (rRNA) | 53 |
| *Gertia stigmatica* K.Takahashi, G.Benico, Wai Mun Lum & M.Iwataki | mdd472-kt | western North Pacific, off Japan, Sagami Bay: Honshū, Kantō, Kanagawa, Manazuru (35°09'N, 139°10'E) | Oct 20, 2016 | K. Takahashi s.n. | LC490696 (rRNA) | 109 |

| | | | | | | |
|---|---|---|---|---|---|---|
| *Gyrodinium heterostriatum* (Kof. & Swezy) F.Gómez, Artigas & Gast | FG1 | North Sea, off the Netherlands: West Flanders, Knokke-Heist (51°40'N, 3°26'E) | May 9, 2019 | F. Gómez [Simon Stevin] 50 | MT677911 (SSU), MW000332 (ITS+LSU) | 110 |
| *Gyrodinium rubrum* (Kof. & Swezy) Y.Takano & T.Horig. | n.inf. (chimeric as used in Orr et al. 2012) | western North Pacific, Sea of Japan, off Japan: Hokkaidō, Shiribeshi, Otaru; North Sea, off Denmark: North Denmark, Hjørring, Hirtshals | Apr 24, 2002; May, 2000 | n.inf. | AB120003 (SSU), AY571369 (LSU) | 111,112 |
| *Gyrodinium spirale* (Bergh) Kof. & Swezy | n.inf. (chimeric as used in Orr et al. 2012) | western North Pacific, Sea of Japan, off Japan: Hokkaidō, Shiribeshi, Otaru; North Sea, off Denmark: North Denmark, Hjørring, Hirtshals | Apr 24, 2002; May, 2000 | n.inf. | AB120001 (SSU), AY571371 (LSU) | 111,112 |
| *Kapelodinium vestifici* (F.Schütt) Boutrup, Moestrup & Daugbjerg | n.inf. | North Atlantic, off Iceland | Aug, 2012 | U. Tillmann [Maria S. Merian] [U. Tillmann] s.n. | KU512737 (LSU) | 113 |
| *Kapelodinium vestifici* (F.Schütt) Boutrup, Moestrup & Daugbjerg | uncultivated clone KJ34-3-25 | western North Pacific, South China Sea (10°59'N, 113°04'E) | Sep 2, 2012 | Anonymous kj34 | KT389952 (ITS+LSU) | 114 |
| *Karenia brevis* (C.C.Davis) Gert Hansen & Moestrup | SP3 | western North Atlantic, Gulf of Mexico, off USA–TX: Brownsville | Oct, 1999 | T.A. Villareal s.n. | AF352820 (SSU), AF352825 (ITS), AY355456 (LSU) | 115,116 |

| | | | | | | |
|---|---|---|---|---|---|---|
| *Karlodinium veneficum* (D.Ballant.) J.Larsen | NCMA1975 (≡ GE2-1) | USA–MD: Princess Anne, Hyrock Farms (38°10'N, 75°44'W) | n.nf. | A. Li & D. Stoecker [A. Li] s.n. | EF036540 (SSU+ITS+LSU) | 117 |
| *Karlodinium zhouanum* Z.Luo & H.Gu | TIO397 | western North Pacific, South China Sea, off China, Daya Bay (22°34'N, 114°36'E) | Nov 11, 2016 | n.inf. | OP445669 (SSU), MG738204 (ITS), MG737358 (LSU) | 118 |
| *Ptychodiscus noctiluca* F.Stein | n.inf. | western South Atlantic, off Brazil, São Sebastião Channel (23°50'S, 45°24'W, –40m) | Apr 22, 2013 | F. Gómez s.n. | KU640194 (SSU) | 119 |
| *Shimiella gracilenta* Ok, H.J.Jeong, S.Y.Lee & Noh (holotype) | SGJH1904 | western North Pacific, East China See, off South Korea: Nanpo, Jinhae Bay (35°07'N, 128°41'E) | Apr, 2019 | n.inf. | MN965778 (SSU+ITS+LSU) | 120 |
| *Takayama acrotrocha* (J.Larsen) de Salas, Bolch & Hallegr. | MC728D5 | Mediterranean Sea, Tyrrhenian Sea, off Italy: Campania, Naples | Aug 22, 2006 | n.inf. | HM067010 (SSU), HM067011 (ITS), FJ024703 (LSU) | 121 |
| *Takayama* sp. | RCC5707 | western South Pacific, Polynesia, Campbell Plateau, off New Zealand | Mar 29, 2017 | [P. Gourvil] TAN1702 | MH764627 (SSU), MH781402 (ITS), MH734606 (LSU) | Gourvil & Vaulot (unpubl. 2018) |
| *Testudodinium* sp. | DF156 | n.inf. | n.inf. | n.inf. | OP070554 (SSU), OP104678 (ITS), OP104743 (LSU) | Guo (unpubl. 2022) |
| *Torodinium teredo* (C.H.G.Pouchet) Kof. & Swezy | FG21-2, AR102 (chimeric) | Mediterranean Sea, off France: Marseille, Endoume Pier (43°17'N, 5°21'E, –3 m); western | Dec, 2007; n.inf. | n.inf.; A. Reñé s.n. | KR139781 (SSU), KP790233 (LSU) | 8,122 |

| | | | | | | |
|---|---|---|---|---|---|---|
| | | Mediterranean Sea, off Spain: Catalonia | | | | |
| †Suessiales | | | | | | |
| *Ansanella granifera* H.J.Jeong, S.H.Jang, Moestrup & N.S.Kang (holotype) | AGSW10 | western North Pacific, Yellow Sea, off South Korea: (37°18'N, 126°36'E) | Sep, 2010 | n.inf. | HG529978 (SSU), HG529979 (ITS), HG529980 (LSU) | 123 |
| *Baldinia anauniensis* Gert Hansen & Daugbjerg (holotype) | greenGS | Italy, Trentino, Lake Tovel | Jul 29, 2003 | n.inf. | EF052682 (SSU), EF052683 (LSU) | 124 |
| *Biecheleria baltica* Moestrup, K.Lindb. & Daugbjerg | WHTV-C1 | Baltic Sea, off Finland: Uusimaa, Raseborg, Tvärminne | n.inf. | A. Kremp Wolo C1 6 | OR686940 (SSU), KR362901 (ITS), AY628430 (LSU), KR362902 (d8d10LSU) | 101, 125,126, Brink et al. (unpubl. 2019) |
| *Biecheleria brevisulcata* K.Takahashi & M.Iwataki (holotype) | trd276-kt | western North Pacific, Sea of Japan, off Japan: Yamagata, Tsuruoka (38°46'N, 139°44'E) | Jul, 2011 | K. Takahashi s.n. | LC068842 (SSU+ITS+LSU) | 127 |
| *Borghiella ovum* A.Müll.bis & Gottschling (holotype) | GeoK*077 | Germany: Bavaria, Lower Bavaria, Rottal-Inn, Reut (48°19'N, 12°56'E, 448m) | Feb 11, 2020 | M. Gottschling & S. Schottenhammel [S. Schottenhammel] D221 | OR693297 (SSU+ITS+LSU), OR693295 (LSUd8d10) | Müller et al. (in press) |
| *Borghiella* aff. *pascheri* (Suchl.) Moestrup | GeoM*868 | Germany: Bavaria, Traunstein, Seeon, peat bog near Brunnen-See (47°59'N, 12°26'E) | Dec 5, 2017 | M. Gottschling & J. Kretschmann [J. Knechtel] D138 | OR943604 (SSU+ITS+LSU) | this study |

| | | | | | | |
|---|---|---|---|---|---|---|
| *Breviolum minutum* (T.C.LaJeunesse, J.E. Parkinson & J.D.Reimer) J.E. Parkinson & T.C.LaJeunesse [isolated from *Orbicella faveolata* (J.Ellis & Sol., 1786)] | Mf1.05b.01 | western North Atlantic, Gulf of Mexico, off USA–FL | n.inf. | n.inf. | BASF01015284 (rRNA) | 128 |
| *Cystodinium phaseolus* Pascher | CCAC2439B (≡ ASW12002, NORCCA K-1112) | Austria: Lower Austria, Must Quarter, Lunzer See | 1984 | E. Kusel-Fetzmann s.n. | OR693298 (SSU+ITS+LSU), OR693296 (LSUd8d10) | 68, Müller et al. (in press) |
| *Dactylodinium pterobelotum* K.Takahashi, Moestrup & M.Iwataki (holotype) | vnd255-kt | western North Pacific, South China Sea, off Vietnam: Bạc Liêu, Nhà Mát (9°12'N, 105°45'E) | Mar 6, 2013 | K. Takahashi s.n. | LC272997 (SSU+ITS+LSU) | 129 |
| *Durusdinium* sp. (isolated from *Haliclona koremella* de Laub., 1954) | PSP1-05 | western North Pacific, Mirconesia, off Palau: Carp Island | Sep, 1997 | n.inf. | AB016578 (SSU), JN558081 (ITS+LSU), AJ308899 (LSU) | 130,131 |
| *Effrenium voratum* (H.J.Jeong, S.Y.Lee, N.S.Kang & LaJeunesse) LaJeunesse & H.J.Jeong | SMFL1, free-living isolate | western North Pacific, East China Sea, off South Korea: Jeju (33°17'N, 126°44'E) | May 1, 2008 | H.S. Kim s.n. | HE653238 (SSU+ITS+LSU) | 132 |
| *Leiocephalium pseudosanguineum* K.Takahashi, Moestrup & M.Iwataki (holotype) | it12-10kt (≡ NIES3777) | Japan: Yamagata, Kenmin-no-Mori, Itabashi (38°14'N, 140°12'E) | May, 2012 | K. Takahashi s.n. | LC068840 (rRNA) | 127 |

| | | | | | | |
|---|---|---|---|---|---|---|
| *Pyramidodinium atrofuscum* T.Horig. & Sukigara (holotype) | HG226 | western North Pacific, Mirconesia, off Palau: Rock Islands, Eil Malk, Jellyfish Lake | Mar 21, 2001 | n.inf. | LC209792 (SSU) | 133 |
| *Sphaerodinium polonicum* var. *tatricum* Wołosz. | Buçaco | Portugal: Centro, Buçaco (40°23'N, 8°22'W, 350–400m) | Jul 7, 2015 | n.inf. | MT584213 (SSU), MT584216 (ITS), MT584211 (LSU) | 134 |
| *Symbiodinium* sp. [isolated from *Plexaura homomalla* (Esper, 1792)] | NCMA2456 (≡ 379) | western North Atlantic, Sargasso Sea, off UK, the Bermudas (32°23'N, 64°41'W) | Jul 29, 2004 | R. Iglesias-P. [R. Iglesias-P.] s.n. | LK934674 (SSU+ITS+LSU) | Lee et al. (unpubl. 2014) |
| <u>Peridiniales</u> | | | | | | |
| *Alatosphaera hermosillae* (Carbonell-Moore) Nézan, Carbonell-Moore, K.N.Mert. & Chomérat | IFR16-223 | western Meditarranean Sea, Gulf of Bastia, off France: Haute-Corse, Furiani (42°40'N, 9°30'E) | Sep 22, 2016 | Y. Baldi s.n. | OP612908 (SSU), OP600083 (LSU) | 135 |
| *Apocalathium malmogiense* (G.Sjöstedt) Craveiro, Daugbjerg, Moestrup & Calado (reference material) | SHTV1 | Baltic Sea, off Finland: Uusimaa, Raseborg, Tvärminne (59°50'N, 23°15'E) | 2002 | A. Kremp s.n. | KF751923 (SSU+ITS+LSU) | 125,136-138 |
| *Archaeperidinium minutum* (Kof.) Jørg. | Mondego | Portugal: Coimbra, Figueira da Foz, Mondego (40°08'N, 8°51'W) | Dec, 2005 | n.inf. | GQ227501 (SSU), GQ227502 (LSU) | 139 |
| *Blastodinium contortum* Chatton [isolated from | n.inf. | eastern North Pacific, off USA–CA: Gulf of | Jun 11, 2008 | n.inf. | FJ228701 (SSU+ITS+LSU) | 140 |

| | | | | | | |
|---|---|---|---|---|---|---|
| *Paracalanus* cf. *parvus* (Claus, 1863)] | | California, station 3 (24°14'N, 110°20'W) | | | | |
| *Blastodinium crassum* Chatton [isolated from *Paracalanus* cf. *parvus* (Claus, 1863)] | n.inf. | eastern North Pacific, off USA–CA: Gulf of California, station 2 (24°13'N, 110°20'W) | Jun 10, 2008 | n.inf. | FJ228702 (SSU+ITS+LSU) | 140 |
| *Caladoa arcachonensis* *Z.*Luo, K.N.Mert. & H.Gu (holotype) | TIO278 | eastern North Atlantic, Bay of Biscay, Arcachon Bay, off France (44°38'N, 1°04'W) | Apr, 2016 | n.inf. | MK012071 (SSU), MK012081 (ITS), MK012076 (LSU) | 61 |
| †*Calciodinellum operosum* Deflandre (reference material) | SZN74 | Mediterranean Sea, Tyrrhenian Sea, off Italy: Campania, Naples (40°43'N, 14°10'W) | n.inf. | M. Montresor | KF751922 (SSU+ITS+LSU) | 125,137,141-144 |
| *Diplopsalis lenticula* Bergh | M2 | western North Atlantic, Gulf of Mexico, off USA–FL | n.inf. | n.inf. | DQ444226 (LSU) | 145 |
| *Duboscquodinium collinii* Grassé [isolated from *Eutintinnus fraknoii* (Daday, 1887)] | VSM11 | western Mediterranean Sea, off France: Alpes-Maritimes, Nice, Villefranche-sur-Mer (43°41'N, 7°19'E) | Sep 10, 2009 | n.inf. | HM483399 (SSU+ITS+LSU) | 36 |

| | | | | | | |
|---|---|---|---|---|---|---|
| *Durinskia oculata* (F.Stein) Gert Hansen & Flaim (epitype) | GeoM*662 (≡ CCAC6039B, CCCM6005) | Czech Republic: Prague, Hlavní město Praha, Vltava (50°08'N, 14°23'E) | Sep, 2015 | J. Kretschmann & M. Gottschling [J. Kretschmann] D043 | KY693722 (SSU+ITS+LSU), KY693725 (LSUd8d10) | 146 |
| *Ensiculifera tyrrhenica* (Balech) Zhun Li, K.N.Mert., Gottschling, H.Gu & H.H.Shin | GeoB*230 | Mediterranean Sea, Ionian Sea, off Italy: Gulf of Taranto (40°07'N, 17°19'E) | Oct 26, 2002 | D. Saracino [M. Kirsch] s.n. | HQ845329 (SSU+ITS+LSU), OR943599 (LSUd8d10) | 136, this study |
| *Gloeodinium montanum* Klebs | CCAC0066 | Germany: Hessen, Marburg, Nordeck | n.inf. | n.inf. | EF058238 (SSU), EF058258 (LSU) | 138 |
| *Heterocapsa arctica* T.Horig. (holotype) | NCMA445 (≡ NCMA35) | North Atlantic, Baffin Bay (76°15'N, 82°33'W) | Jun 3, 1986 [Jul 28, 1989] | R. Selvin s.n. | KF925338 (SSU), JQ972677 (ITS), AY571372 (LSU) | 74,111,147, Preston & Gilg (unpubl. 2014) |
| *Heterocapsa horiguchii* Iwataki, H.Takay. & Matsuoka | HH-2003PS-01 | western North Pacific, East China Sea, off South Korea: Jeju | Mar 11, 2020 | n.inf. | OP970968 (rRNA) | Kang et al. (unpubl. 2022) |
| *Heterocapsa pseudotriquetra* Iwataki, Gert Hansen & Fukuyo | HP-HD1804-01 | western North Pacific, East China Sea, off South Korea: Jeju | Apr 12, 2018 | n.inf. | OP968025 (rRNA) | Kang et al. (unpubl. 2022) |
| *Huia caspica* (Ostenf.) H.Gu, K.N.Mert. & T.Liu | HBI:SD201204a | China: Shandong, Qingdao, Jihongtan (36°21'N, 120°13'E) | Oct 21, 2012 | n.inf. | KJ995958 (SSU), KJ995959 (LSU) | 148 |
| *Islandinium minutum* subsp. *barbatum* É.Potvin, So- | UNID1 | Arctic Ocean (75°22'N, 176°19'E, –346m) | Aug 31, 2015 | Anonymous [Araon] ARA06C-5 | KY129817 (SSU+ITS+LSU) | 149 |

| | | | | | | |
|---|---|---|---|---|---|---|
| Young Kim, E.J.Yang & M.J.Head | | | | | | |
| *Kryptoperidinium triquetrum* (Ehrenb.) Tillmann, Gottschling, Elbr., Kusber & Hoppenrath | GeoB 459 (≡ CCAC4765B, CCCM327) | Mediterranean Sea, Aegean Sea, off Greece: Peloponnese, Argolis, Nafplio, Nea Kios (37°35'N, 22°45'E) | Mar, 2010 | C. Zinßmeister & S. Söhner [M. Kirsch] GRI00027 | KY693721 (SSU+ITS+LSU), KY693724 (LSUd8d10) | 146 |
| *Laciniporus arabicus* Saburova & Chomérat | Om-L-arabic-029 | Indian Ocean, Arabian Sea, off Oman: Dhofar, Salalah (16°50'N, 54°42'E) | Feb 24, 2014 | n.inf. | MH029284 (SSU), MH029283 (LSU) | 150 |
| *Lessardia elongata* Saldarr. & F.J.R.Taylor | SPMC104 | n.inf. | n.inf. | n.inf. | AF521100 (SSU) | 151,152 |
| *Mysticella* sp. | IFR16-399 | western Meditarranean Sea, Gulf of Bastia, off France: Haute-Corse, Furiani (42°40'N, 9°30'E) | Sep 22, 2016 | Y. Baldi s.n. | OP612925 (SSU), OP600100 (LSU) | 135 |
| *Nottbeckia ochracea* (Levander) Gert Hansen, Daugbjerg & Moestrup (reference material) | GH957 | Finland: Uusimaa, Raseborg, Tvärminne, Brännskär | Jun 19, 2011 | n.inf. | MG754078 (SSU), MG754079 (LSU) | 153 |
| *Pachena abriliae* A.Reñé, Satta & Hoppenrath | Castelldefels | westren Mediterranean Sea, off Spain: Barcelona, Castelldefels (41°16'N, 1°56'E) | 2017 | A. Reñé s.n. | MN707940 (SSU), MN703810 (LSU) | 154 |

| | | | | | | |
|---|---|---|---|---|---|---|
| *Palatinus apiculatus* (Ehrenb.) Craveiro, Calado, Daugbjerg & Moestrup (epitype) | GeoM*762 (≡ CCAC6788B) | Germany: Berlin, Mitte, Tiergarten (52°31'N, 13°21'E) | Mar 28, 2016 | M. Gottschling [J. Kretschmann] D047 | KY996787 (SSU+ITS+LSU), MG255412 (LSUd8d10) | 155 |
| *Parvodinium marciniakii* Kretschmann, Owsianny, Zerdoner & Gottschling (holotype) | GeoM*709 (≡ CCAC6785B) | Poland: Lesser Poland, Tatra, Zielony Staw Gąsienicowy (49°14'N, 20°00'E, 1672m) | Sep 22, 2015 | P.M. Owsianny, G. Marciniak & K. Trawiński [J. Kretschmann] PL021 | MG255422 (SSU+ITS+LSU), MG255414 (LSUd8d10) | 156 |
| *Parvodinium mixtum* Wołosz. ex Kretschmann, Owsianny, Zerdoner & Gottschling (holotype) | GeoM*720 (≡ †CCAC8952B) | Poland: Lesser Poland, Tatra, Litworowy Staw Gąsienicowy (49°14'N, 20°00'E, 1618m) | Sep 22, 2015 | P.M. Owsianny, K. Trawiński & G. Marciniak [J. Kretschmann] PL018 | MG255425 (SSU+ITS+LSU), MG255417 (LSUd8d10) | 156 |
| *Peridinium bipes* forma *globosum* Er.Lindem. | NIES495 (≡ LOND9) | Japan: Fukushima, Lake Onogawa | Jul 30, 1985 | [T. Sawaguchi s.n.] | GU046392 (SSU+ITS+LSU) | 157,158 |
| *Peridinium cinctum* (O.F.Müll.) Ehrenb. | CCAC0102 (≡ M1576/1) | Germany: Lower Saxony, Wittmund, Spiekeroog | 1998 | [D. Hille s.n.] | EF058244 (SSU), KF751925 (SSU+ITS+LSU), MF423370 (LSUd8d10) | 68,136,137, 144,159 |
| *Peridinium volzii* Lemmerm. | GeoK*024 (≡ CCAC9311B, CCAP1140/4, CCCM6006) | Poland: Lesser Poland, Tatra, Morskie Oko | Oct 1, 2018 | P.M. Owsianny [J. Knechtel] PL156 | MW784572 (SSU+ITS+LSU), MW784609 (LSUd8d10) | 160 |
| †*Pernambugia tuberosa* (Kamptner) Janofske & Karwath (reference material) | GeoB*61 (≡ CCAC4752B, CCAP1141/1) | western South Atlantic (11°32'S, 28°35'W, –100m) | Feb 27, 1997 | [Meteor 38/1] [M. Kirsch] 4321-9 | KR362907 (SSU), JN982372 (ITS+LSU), MF423371 (LSUd8d10) | 126,136,137, 144,159 |

| | | | | | | |
|---|---|---|---|---|---|---|
| *Pfiesteria piscicida* Steid. & J.M.Burkh. | n.inf. | USA–MD: Chicamacomico River | 1997 | K.A. Steidinger & J.M. Burkholder s.n. | AY112746 (rRNA) | 161 |
| *Podolampas palmipes* F.Stein | IFR16-313 | western Indian Ocean, off France: Réunion, Saint-Pierre, Petite-Île (21°22'S, 55°32'E) | Nov 8, 2016 | A. Tunin-Ley s.n. | OP612916 (SSU), OP600091 (LSU) | 135 |
| *Preperidinium meunieri* (Pavill.) Elbr. | YT9 | western North Pacific, Yellow Sea, off China | n.inf. | n.inf. | ON392304 (LSU) | 162 |
| "*Protoperidinium*" *claudicans* (Paulsen) Balech | #2 (cyst) | western North Pacific, Sea of Japan, off Japan: Hokkaidō, Ishikari (43°13'N, 141°18'E) | Jul 17, 2004 | n.inf. | AB255833 (SSU), AB255842 (LSU) | 163 |
| *Protoperidinium pellucidum* Bergh | #28 | western North Pacific, Sea of Japan, off Japan: Hokkaidō, Shiribeshi, Otaru (43°10'N, 141°01'E) | Oct 31, 2002 | n.inf. | AB181903 (SSU), AB255862 (LSU) | 163,164 |
| *Rhinodinium broomeense* Sh.Murray, Hoppenrath, Yoshimatsu, Toriumi & J.Larsen | n.inf. | eastern Indian Ocean, off Australia: Western Australia, Broome, Town Beach (17°58'S, 122°14'E) | Sep, 2003 | n.inf. | DQ078782 (LSU) | 165 |
| *Scrippsiella lachrymosa* Jane Lewis ex M.J.Head | n.inf. | n.inf. | n.inf. | n.inf. | LT993764 (SSU+ITS+LSU) | Jeong (unpubl. 2018) |

| | | | | | | |
|---|---|---|---|---|---|---|
| *Scrippsiella* cf. *sweeneyae* Balech | CCCM280 | n.inf. | n.inf. | A. Chan s.n. | HQ845331 (SSU+ITS+LSU) | 136 |
| *Thoracosphaera heimii* (Lohmann) Kamptner | CCCM670 (≡ NCMA1069) | western North Atlantic, Gulf of Mexico | Apr 23, 1980 | L. Brand s.n. | HQ845327 (SSU+ITS+LSU), OR943600 (LSUd8d10) | 60,67, this study |
| *Tintinnophagus acutus* Coats (isolated from *Tintinnopsis cylindrica* Daday, 1887) (holotype) | n.inf. | western North Atlantic, Chesapeake Bay, off USA–MD: Rhode River (38°53'N, 76°33'W) | n.inf. | n.inf. | HM483397 (SSU+ITS+LSU) | 36 |
| *Unruhdinium penardii* (Lemmerm.) Gottschling | Upen2_Uniss | Italy: Sardinia, Nuoro, Lake Cedrino (40°20'N, 9°33'E) | Mar, 2017 | n.inf. | MW194106 (SSU), MW195007 (ITS), MW217558 (LSU) | 166 |
| *Vulcanodinium rugosum* Nézan & Chomérat | G | France | n.inf. | n.inf. | MG826115 (SSU), MG826367 (ITS), MG826107 (LSU) | 59 |
| *Zooxanthella nutricula* K.Brandt (isolated from *Thalassicolla nucleata* Huxley, 1851) | BBSR323 | western North Atlantic, Sargasso Sea, off UK, the Bermudas: 3–5 miles SE of Bermuda | n.nf. | n.nf. | U52356 (SSU), KC511788 (ITS+LSU), OR943601 (LSUd8d10) | 52,167, this study |
| Gonyaulacales | | | | | | |
| *Ailadinium reticulatum* Saburova & Chomérat (reference material) | IFR13-227, IFR13-173 (chimeric) | Red Sea, off Jordan, Gulf of Aqaba (29°26'N, 34°58'E) | Oct, 2010; Oct, 2011 | M. Saburova s.n. | KJ187034 (SSU), KJ187036 (LSU) | 168 |

| | | | | | | |
|---|---|---|---|---|---|---|
| *Alexandrium catenella* (Whedon & Kof.) Balech | ACC01 | eastern South Pacific, off Chile: Aysen, Canal Costa (45°24'S, 72°40'W) | 1994 | n.inf. | JN098269 (SSU), KF646308 (ITS+LSU), HQ832891 (LSU) | 169,170 |
| *Alexandrium minutum* Halim | NCMA113 (≡ AL, AL1V) | eastern North Atlantic, off Spain: Galicia, Ría de Vigo (42°14'N, 8°48'W) | Sep 1, 1987 | I. Bravo [I. Bravo] s.n. | AY831408 (rRNA) | 25,171-173, Ferrell & Beaton (unpubl. 2008) |
| *Alexandrium pseudogoniaulax* (Biecheler) T.Horig. ex Yuki & Fukuyo | APBG03 | South China Sea, Beibu Gulf, off China: Guangxi, Weizhou (21°04'N, 109°06'E) | Jun 10, 2019 | n.inf. | MW362482 (SSU), MW443025 (ITS), MW362466 (LSU) | 174 |
| *Alexandrium tamarense* (M.Lebour) Balech (epitype) | ATSW01-1 (≡ RCC4087) | North Sea, off Sweden: Västernorrland, Sundsvall, Essvik (58°17'N, 11°35'E) | Oct 26, 1991 | O. Lindahl [D. Kulis] s.n. | JN626284 (SSU), KF646458 (LSU) | 170 |
| *Alexandrium taylorii* Balech | AY7T (≡ CoSMi1017) | Mediterranean Sea, Adriatic Sea, Lagoon of Marano, off Italy: Friuli-Venezia Giulia, Trieste | May, 2004 | A. Beran s.n. | MT644478 (ITS), MT643180 (LSU) | 175,176 |
| *Carinadinium ovatum* (Yoshimatsu, S.Toriumi & J.D.Dodge) Hoppenrath, Selina & Yamag. | Tov5 | western North Pacific, off Japan: Kōchi, Aki, Tōyō, Kannoura | Apr, 2014 | A. Yamaguchi | MW842493 (SSU), MW842483 (LSU) | 177 |
| *Ceratium furcoides* (Levander) Langhans | HBI:SC201002a | n.inf. | n.inf. | n.inf. | JQ639757 (SSU), JQ639769 (ITS), JQ639748 (LSU) | Zhang et al. (unpubl. 2012) |

| | | | | | | |
|---|---|---|---|---|---|---|
| *Coolia canariensis* S.Fraga | CMJJ1 | western North Pacific, East China Sea, off South Korea: Jeju, Seongsan (33°28'N, 126°56'E) | Oct, 2009 | n.inf. | FR846195 (SSU), FR846193 (ITS), FR846194 (LSU) | 178 |
| †*Dapsilidinium pastielsii* (R.J.Davey & G.L.Williams) J.P.Bujak, C.Downie, G.L.Eaton & G.L.Williams | n.inf. | western North Pacific, East China Sea, off Japan: Okinawa, Shioya Bay (26°40'N, 128°06'E) | Feb 10, 2011 | K.N. Mertens, K. Matsuoka & K. Hinode s.n. | AB919106 (SSU), AB919107 (LSU) | 179 |
| *Gonyaulax amoyensis* H.Gu. & K.N.Mert. | TIO708 | western North Pacific, East China Sea, off China: Fujian, Xiamen (24°36'N, 118°09'E) | Jan 30, 2018 | n.inf. | OM177648 (SSU), OM228733 (ITS), OM228714 (LSU) | 180 |
| *Grammatodinium tongyeonginum* Zhun Li & H.H.Shin (holotype) | LIM-PS2334 | western North Pacific, East China Sea, off South Korea: Tongyeong (34°45'N, 128°23'E) | Dec 18, 2013 | n.inf. | KX180048 (SSU), KS180049 (LSU) | 181 |
| *Halostylodinium arenarium* T.Horig. & Yoshizawa-Ebeta (reference material) | HG2 | western North Pacific, East China Sea, off Japan: Okinawa, Ishigaki | n.inf. | M. Higashi s.n. | LC054931 (SSU) | 182 |
| *Lingulodinium polyedra* (F.Stein) J.D.Dodge | n.inf. | Korea | n.inf. | n.inf. | AF377944 (rRNA) | Lee et al. (unpubl. 2001) |
| *Pentaplacodinium usupianum* Z.Luo, Leaw & H.Gu | DBS01 | Malaysia: Sarawak, Kuching, Semariang (1°36'N, 110°19'E) | Dec 21, 2016 | [S.T. Teng] s.n. | MN137905 (SSU), MN137897 (ITS) | 183 |
| *Pyrocystis noctiluca* J.Murray & Haeckel | NCMA732 (≡ NCMA4) | eastern North Pacific, off USA–CA: Santa Barbara | Nov 21, 1977 | B. Sweeney [B. Sweeney] s.n. | AF022156 (SSU), FJ939576 (LSU) | 184, Yu et al. (unpubl. 2009) |

| | | | | | | |
|---|---|---|---|---|---|---|
| | | Channel (34°15'N, 119°43'W) | | | | |
| *Pyrrhotriadinium polyedricum* (C.H.G.Pouchet) Nakada | G1 | western South Atlantic, off Brazil: São Paulo, Ubatuba (23°33'S, 45°07'W) | Feb 28, 2014 | F. Gómez s.n. | KM886380 (SSU+ITS+LSU) | 185 |
| †*Pyxidinopsis psilata* (D.Wall & B.Dale) M.J.Head | LH3 | Caspian Sea, off Iran (37°31N, 49°55'E, –25m) | Sep 3, 2011 | S. Bagheri s.n. | KY681700 (SSU), MT041629 (ITS), MT039439 (LSU) | 186,187 |
| †*Spiniferites scabratus* D.Wall | TIO706 | western Meditarranean Sea, off France: Haute-Corse, Corte, Diane Lagoon (42°08'N, 9°32'E) | Jan 18, 2016 | n.inf. | MW775719 (SSU), MW775687 (ITS), MW775711 (LSU) | 188 |
| *Thecadinium kofoidii* (Herdman) J.Schiller | Th.k-1 | western North Pacific, Sea of Japan (43°04'N, 137°57'E) | Feb 3, 2013 | n.inf. | KY575427 (SSU), KY575428 (ITS), KY575425 (LSU), KY575426 (LSUd8d10) | 189 |
| "*Thecadinium*" *yashimaense* Yoshimatsu, Toriumi & J.D.Dodge | NCMA1890 (≡ CCCM682) | eastern North Pacific, Boundary Bay, off Canada: British Columbia (49°00'N, 123°00'W) | Apr, 1988 | D. Jacobsen [E. Simons] s.n. | AY238477 (SSU), FJ823640 (ITS), GU295209 (LSU) | 74,190-193 |

| | | | | | | |
|---|---|---|---|---|---|---|
| *Tripos longipes* (Bailey) F.Gómez | NCMA1770 | western North Atlantic, off USA–ME: West Boothbay Harbor, Bigelow (43°51'N, 69°38'W) | Jan 9, 1997 | S.L. Morton [S.L. Morton] s.n. | DQ388462 (SSU), EU927566 (ITS), EU165305 (LSU) | 192,194, Ferrell & Beaton (unpubl. 2008) |
| <u>Dinophysales</u> | | | | | | |
| *Amphisolenia bidentata* Schröd. | LE392 (chimeric as used in Orr et al. 2012) | Indian Ocean, off Australia (16°02'S, 119°20'E) | fall 2006 | n.inf. | GU196149 (SSU), FJ808682 (LSU) | 195,196 |
| *Dinophysis caudata* Kent | CBC4L3 | western North Atlantic, Chesapeake Bay, off USA–VA (36°20'N, 74°44'W) | Oct 15, 2007 | n.inf. | EU780640 (SSU+ITS+LSU) | 197 |
| *Dinophysis caudata* Kent | FTL69 | western North Atlantic, off USA–FL: Ft. Lauderdale (26°05'N, 80°03'W) | Feb 26, 2008 | n.inf. | EU780644 (SSU+ITS+LSU) | 197 |
| *Dinophysis caudata* Kent | n.inf. | eastern North Atlantic, off Spain: Galicia, Pontevedra, Vigo | n.inf. | n.inf. | KF871416 (SSU), KF871409 (LSUd8d10) | Raho & Marin (unpubl. 2013) |
| *Histioneis* sp. | FTL62 | western North Atlantic, off USA–FL: Ft. Lauderdale (26°05'N, 80°03'W) | Feb 26, 2008 | n.inf. | EU780646 (SSU+ITS+LSU) | 197 |

| | | | | | | |
|---|---|---|---|---|---|---|
| *Ornithocercus magnificus* F.Stein | CBC4L7 | western North Atlantic, Chesapeake Bay, off USA–VA (36°20'N, 74°44'W) | Oct 15, 2007 | n.inf. | EU780649 (SSU+ITS+LSU) | 197 |
| *Prodinophysis rapa* (F.Stein) Balech | CBC4L5 | western North Atlantic, Chesapeake Bay, off USA–VA (36°20'N, 74°44'W) | Oct 15, 2007 | n.inf. | EU780655 (SSU+ITS+LSU) | 197 |
| <u>Prorocentrales</u> | | | | | | |
| *Adenoides sinensis* H.Gu, Xintian Li & Z.Luo (holotype) | TIO303 | western North Pacific, Yellow Sea, off China: Shandong, Qingdao (36°03'N, 120°22'E) | Oct 15,2015 | n.inf. | MF535295 (SSU), OQ091753 (ITS), MF535292 (LSU) | 198 |
| *Chrysodinium ballux* (N.Yamada, Dawut, R.Terada & T.Horig.) F.Gómez, Y.Nakam. & L.F.Artigas (holotype) | HG177 | western North Pacific, off Japan: Kyūshū, Kagoshima, Takeshima (30°49'N, 130°24′E) | May 10, 2011 | T. Horiguchi | LC054938 (SSU), LC375159 (LSU) | 199 |
| *Corythodinium cristatum* (Kof.) F.J.R.Taylor | FG28 | western South Atlantic, off Brazil (25°33'S, 44°58'W, −1000m) | Jun 23, 2015 | n.inf. | KY421383 (SSU) | 200 |
| *Corythodinium cristatum* (Kof.) F.J.R.Taylor | FG30 | western South Atlantic, off Brazil (25°33'S, 44°58'W, −1000m) | Jun 23, 2015 | n.inf. | KY421374 (SSU) | 200 |
| *Corythodinium* cf. *cristatum* (Kof.) F.J.R.Taylor | SC108 | n.inf. | n.inf. | n.inf. | ON660941 (SSU) | Sarno (unpubl. 2022) |

| | | | | | | |
|---|---|---|---|---|---|---|
| *Corythodinium frenguellii* (Rampi) F.J.R.Taylor | FG8 | western South Atlantic, off Brazil, São Sebastião Channel (23°50'S, 45°24'W, –40m) | Jun 13, 2013 | n.inf. | KY421382 (SSU) | 200 |
| *Corythodinium frenguellii* (Rampi) F.J.R.Taylor | FG7 | western South Atlantic, off Brazil, São Sebastião Channel (23°50'S, 45°24'W, –40m) | May 2, 2013 | n.inf. | KY421380 (SSU) | 200 |
| *Corythodinium frenguellii* (Rampi) F.J.R.Taylor | FG46 | western South Atlantic, off Brazil: Ubatuba (23°32'S, 45°06'W, –15m) | Jun 19, 2015 | n.inf. | KY421381 (SSU) | 200 |
| *Corythodinium tessellatum* (F.Stein) Loebl. & A.R.Loebl. | FG9 | western South Atlantic, off Brazil, São Sebastião Channel (23°50'S, 45°24'W, –40m) | May 15, 2013 | n.inf. | KY421378 (SSU) | 200 |
| *Corythodinium tessellatum* (F.Stein) Loebl. & A.R.Loebl. | FG40 | western South Atlantic, off Brazil, São Sebastião Channel (23°50'S, 45°24'W, –40m) | Aug 22, 2013 | n.inf. | KY421377 (SSU) | 200 |
| *Corythodinium tessellatum* (F.Stein) Loebl. & A.R.Loebl. | FG42 | western South Atlantic, off Brazil: Ubatuba (23°32'S, 45°06'W, –15m) | Nov 27, 2015 | n.inf. | KY421379 (SSU) | 200 |
| *Madanidinium loirii* Chomérat | IFR-MLO-01M | western North Atlantic,Caribbean Sea, off France: Martinique (14°32'N, 61°05'W) | Apr 6, 2013 | n.inf. | KF751601 (SSU), KF751602 (LSU) | 201 |

| | | | | | | |
|---|---|---|---|---|---|---|
| *Oxytoxum lohmannii* Tillmann & Gottschling | AR19 | western Mediterranean Sea, off Spain: Catalonia | n.inf. | A. Reñé s.n. | KP790175 (SSU) | 8 |
| *Oxytoxum lohmannii* Tillmann & Gottschling | AR67 | western Mediterranean Sea, off Spain: Catalonia | n.inf. | A. Reñé s.n. | KP790176 (SSU) | 8 |
| *Oxytoxum lohmannii* Tillmann & Gottschling | K-AC-E10 | Baltic Sea, off Germany: Schleswig-Holstein, Kiel (54°20'N, 10°09'E) | Sep 19, 2019 | U. Tillmann, M. Gottschling & H. Gu [U. Tillmann] s.n. | OR943602 (SSU+ITS+LSU) | this study |
| *Oxytoxum lohmannii* Tillmann & Gottschling | n.inf. | eastern North Atlantic, off USA–WA: Puget Sound | n.inf. | n.inf. | AF274254 (SSU) | 67 |
| *Oxytoxum scolopax* F.Stein | FG11 | western South Atlantic, off Brazil, São Sebastião Channel (23°50'S, 45°24'W, –40m) | Aug 7, 2013 | n.inf. | KY421376 (SSU) | 200 |
| *Oxytoxum scolopax* F.Stein | FG43 | western South Atlantic, off Brazil: Ubatuba (23°32'S, 45°06'W, –15m) | Nov 1, 2015 | n.inf. | KY421375 (SSU) | 200 |
| *Pileidinium ciceropse* Tamura & T.Horig. | n.inf. | western North Pacific, Mirconesia, off Palau: Mecherchar Island | May 22, 2002 | n.inf. | AB211357 (SSU) | 202 |
| *Plagiodinium* sp. | HG225 | western North Pacific, off Japan: Kyūshū, Kagoshima, Takeshima (30°49'N, 130°24’E) | May 30, 2011 | T. Horiguchi | LC054937 (SSU), LC375160 (LSU) | 199 |

| | | | | | | |
|---|---|---|---|---|---|---|
| *Planodinium striatum* R.D.Saunders & J.D.Dodge | FG36 | eastern North Atlantic, Celtic Sea, English Channel, off France: Hauts-de-France, Pas-de-Calais, Wimereux (50°46'N, 1°37'E) | Jun, 2012 | F. Gómez s.n. | LC507455 (SSU) | 203 |
| *Prorocentrum caipirignum* S.Fraga, M.Menezes & S.Nascimento | PMHV1 | Cuba | 2005 | [A. Reyes] s.n. | JQ638940 (SSU+ITS+LSU), JQ638945 (LSU) | 75,204 |
| *Prorocentrum clipeus* Hoppenrath | IFR470 | eastern North Atlantic, off France: Brittany, Morbihan, Groix Island | Jul 31, 2007 | N. Chomérat s.n. | JX912175 (LSU) | 205 |
| *Prorocentrum cordatum* (Ostenf.) J.D.Dodge | D127 | eastern Indian Ocean, off South Korea: Tongyeong | n.inf. | n.inf. | JX402086 (rRNA) | Cheon & Ki (unpubl. 2013) |
| *Prorocentrum* cf. *emarginatum* Fukuyo | X2P3 | western North Pacific, South China Sea, off China: Hainan, Sanya (18°14'N, 109°22'E) | Aug 19, 2014 | n.inf. | KY010245 (ITS), KY010260 (LSU) | 206 |
| *Prorocentrum fukuyoi* Sh.Murray & Y.Nagahama | W091 | western North Pacific, South China Sea | n.inf. | n.inf. | MK547117 (SSU), MK605054 (ITS), MK605081 (LSU), MK544031 (LSUd8d10) | Yiu et al. (unpubl. 2020) |

| | | | | | | |
|---|---|---|---|---|---|---|
| *Prorocentrum glenanicum* Chomérat & Nézan | IFR-818, IFR12-080 (chimeric) | eastern North Atlantic, Celtic Sea, off France: Brittany; western North Atlantic, Caribbean Sea, off France: Martinique | Jul 31, 2007; Apr 4, 2011 | M. Loir s.n.; | GU327678 (SSU), JX912179 (LSU) | 207,208 |
| *Prorocentrum koreanum* M.S.Han, S.Y.Cho & P.B.Wang | BGERL02 | western North Pacific, South China Sea, Beibu Gulf, off China | n.inf. | Y. Xu s.n. | MW979822 (SSU), MW999295 (ITS), MW979767(LSU) | Xu (unpubl. 2021) |
| *Prorocentrum leve* M.A.Faust, Kibler, Vandersea, P.A.Tester & Litaker (holotype) | NCMA2634 | western North Atlantic, Caribbean Sea, off Belize: Stann Creek, Twin Cays (16°50N, 88°06'W) | May 21, 2002 | S. Kibler [S. Kibler] s.n. | DQ238043 (SSU+ITS+LSU) | 209 |
| *Prorocentrum lima* (Ehrenb.) F.Stein | NCMA685 (≡ PL2V) | eastern North Atlantic, off Spain: Galicia, Ría de Vigo (42°14'N, 8°48'W) | n.inf. | I. Bravo s.n. | AB189765 (ITS), DQ336179 (LSU) | Ferrell & Beaton (unpubl. 2008), 210,211 |
| *Prorocentrum* cf. *lima* (Ehrenb.) F.Stein | SKLMP_W074 | western North Pacific, South China Sea | n.inf. | n.inf. | MK547120 (SSU), MK605056 (ITS), MK544035 (LSUd8d10) | Yiu et al. (unpubl. 2020) |
| *Prorocentrum micans* Ehrenb. (epitype) | A10 | Baltic Sea, off Germany: Schleswig-Holstein, Kiel (54°21'N, 10°09'E) | Oct 20, 2017 | K.J.S. Meier [U. Tillmann A10] s.n. | MK405477 (rRNA) | 212 |
| *Prorocentrum redfieldii* Bursa | S019 | western North Pacific, South China Sea | n.inf. | n.inf. | MK547148 (SSU), MG914046 (ITS+LSU), MK605103 (LSU), | Yiu et al. (unpubl. 2019) |

| | | | | | | |
|---|---|---|---|---|---|---|
| | | | | | MK544064 (LSUd8d10) | |
| *Prorocentrum rhathymum* A.R.Loebl., Sherley & R.J.Schmidt | PXPV1 | eastern North Pacific, Gulf of California, off Mexico: Baja California, Bahía de La Paz | 2003 | [D. Góngora] s.n. | JQ616822 (SSU+ITS+LSU), JQ616832 (LSU) | 75 |
| *Prorocentrum* cf. *sculptile* M.A.Faust | TIO968 | western Pacific, Celebes Sea, off Indonesia: Manado (1°46'N, 125°02'E) | May 27, 2019 | n.inf. | OP765278 (ITS), OP764420 (LSU) | 213 |
| *Prorocentrum* sp. | IRTA002 | Mediterranean Sea, Balearic Sea, off Spain: Catalonia | n.inf. | n.inf. | FJ160591 (SSU), FJ160593 (ITS), FJ160592 (LSU) | Caillaud et al. (unpubl. 2011) |
| *Pseudadenoides kofoidii* (Herdman) F.Gómez, R.Onuma, Artigas & T.Horig. | NCMA1891 (≡ NEPCC683a) | eastern North Pacific, Boundary Bay, off Canada: British Columbia (49°00'N, 123°00'W) | Apr 1, 1988 | D. Jacobsen [E. Simons] s.n. | KX000289 (SSU), JX262493 (ITS), KX000293 (LSU) | 25,214, Yu et al. (unpubl. 2009) |

## References


1 Lohmann, H. Untersuchungen zur Feststellung des vollständigen Gehaltes des Meeres an Plankton. *Wiss. Meeresuntersuch., Abt. Kiel* **10**, 129–370 (1908).

2 Hällfors, G. Checklist of Baltic Sea phytoplankton species (including some heterotrophic protistan groups). *Balt. Sea Environ. Proc.* **95**, 1–208 (2004).

3 Voltolina, D. The phytoplankton of Liverpool Bay (1977-1978). An annotated checklist. *Nova Hedwigia* **37**, 403–434 (1983).

4 Krakhmalny, A. F. *et al.* Revision of the dinoflagellate species composition of the Black Sea. *Algologia* **28**, 428—448 (2018).

5 Seuthe, L., Iversen, K. R. & Narcy, F. Microbial processes in a high-latitude fjord (Kongsfjorden, Svalbard): II. Ciliates and dinoflagellates. *Polar Biol.* **34**, 751–766 (2011).

6 Schröder, B. Adriatisches Phytoplankton. *Sitzungsber. Kaiserl. Akad. Wiss., Wien, Math.-Naturwiss. Cl., Abt. 1* **120**, 601–657 (1911).

7 Gómez, F. Checklist of Mediterranean free-living dinoflagellates. *Bot. Mar.* **46**, 215–242 (2003).

8 Reñé i Vicente, A., Camp, J. & Garces, E. Diversity and phylogeny of Gymnodiniales (Dinophyceae) from the NW Mediterranean Sea revealed by a morphological and molecular approach. *Protist* **166**, 234–263 (2015).

9 Lebour, M. V. *The dinoflagellates of the Northern seas*. (Marine Biological Association, 1925).

10 Hoppenrath, M., Elbrächter, M. & Drebes, G. *Marine phytoplankton. Selected microphytoplankton species from the North Sea around Helgoland and Sylt*. (Schweizerbart, 2009).

11 Hulburt, E. M. The taxonomy of unarmored Dinophyceae of shallow embayments on Cape Cod, Massachusetts. *Biol. Bull.* **112**, 196–219 (1957).

12 Campbell, P. H. *Studies on brackish water phytoplankton.* (University of North Carolina, 1973).

13 Bérard-Therriault, L., Poulin, M. & Bossé, L. Guide d´identification du phytoplancton marin de l´estuaire et du golfe de Saint-Laurent incluant également certaines protozoaires. *Publ. spéc. can. sci. hal. aquat.* **128**, 1–387 (1999).

14 Hsiao, S. I. C. A checklist of marine phytoplankton and sea ice microalgae recorded from Arctic Canada. *Nova Hedwigia* **37**, 225–313 (1983).

15 Wulff, A. Über das Kleinplankton der Barentssee. *Wiss. Meeresuntersuch., Abt. Helgoland* **13**, 95–125 (1916).

16 Throndsen, J., Hasle, G. R. & Tangen, K. *Phytoplankton of Norwegian coastal waters*. (Almater, 2007).

17 Hansen, P. J. Prey size selection, feeding rates and growth dynamics of heterotrophic dinoflagellates with special emphasis on *Gyrodinium spirale*. *Mar. Biol.* **114**, 327–334 (1992).

18 Hansen, P. J. & Calado, A. J. Phagotrophic mechanisms and prey selection in free-living dinoflagellates. *J. Eukaryot. Microbiol.* **46**, 382–389 (1999).

19 Tillmann, U., John, U. & Cembella, A. On the allelochemical potency of the marine dinoflagellate *Alexandrium ostenfeldii* against heterotrophic and autotrophic protists. *J. Plankton Res.* **29**, 527–543 (2007).

20 Tillmann, U., Alpermann, T., John, U. & Cembella, A. Allelochemical interactions and short-term effects of the dinoflagellate *Alexandrium* on selected photoautotrophic and heterotrophic protists. *Harmful Algae* **7**, 52–64 (2008).

21 Strom, S. *et al.* Chemical defense in the microplankton I: Feeding and growth rates of heterotrophic protists on the DMS-producing phytoplankter *Emiliania huxleyi*. *Limnol. Oceanogr.* **48**, 217–229 (2003).

22 Strom, S., Wolfe, G., Slajer, A., Lambert, S. & Clough, J. Chemical defense in the microplankton II: Inhibition of protist feeding by β-dimethylsulfoniopropionate (DMSP). *Limnol. Oceanogr.* **48**, 230–237 (2003).

23 Nielsen, L. T. & Kiorboe, T. Feeding currents facilitate a mixotrophic way of life. *Isme J.* **9**, 2117–2127 (2015).

24 Chu, F. L. E., Lund, E. D., Littreal, P. R., Ruck, K. E. & Harvey, E. Species-specific differences in long-chain n-3 essential fatty acid, sterol, and steroidal ketone production in six heterotrophic protist species. *Aquat. Biol.* **6**, 159–172 (2009).

25 Orr, R. J. S., Murray, S. A., Stüken, A., Rhodes, L. & Jakobsen, K. S. When naked became armored: An eight-gene phylogeny reveals monophyletic origin of theca in dinoflagellates. *PLoS One* **7**, e50004 (2012).

26 Gu, H. *et al.* Waking the dead: Morphological and molecular characterization of extant †*Posoniella tricarinelloides* (Thoracosphaeraceae, Dinophyceae). *Protist* **164**, 583–597 (2013).

27 Price, D. C. & Bhattacharya, D. Robust Dinoflagellata phylogeny inferred from public transcriptome databases. *J. Phycol.* **53**, 725–729 (2017).

28 Loeblich III., A. R. Dinoflagellate evolution: Speculation and evidence. *J. Protozool.* **23**, 13–28 (1976).

29 Taylor, F. J. R. On dinoflagellate evolution. *Biosystems* **13**, 65–108 (1980).

30 Janouškovec, J. *et al.* Major transitions in dinoflagellate evolution unveiled by phylotranscriptomics. *Proc. Natl. Acad. Sci. USA* **114**, E171–E180 (2017).

31 Balech, E. Sur la tabulation de *Podolampas* et *Oxytoxum*. *Rapp. Commun. Parvenus Avant Congr. Sect. 17, VIII Internatl. Bot. Congr.*, 114–116 (1954).

32 Balech, E. Microplancton de la campaña oceanográfica productividad III. *Revista Mus. Argent. Ci. Nat., Bernardino Rivadavia Inst. Nac. Invest. Ci. Nat. Hidrobiol.* **3**, 1–202 (1971).

33 Balech, E. Microplancton del Atlántico Ecuatorial Oeste (Equalant I). *Publ. Rep. Argent., Ser, Hidrogr, Naval* **H.654**, 1–103 (1971).

34 Dodge, J. D. & Saunders, R. D. A partial revision of the genus *Oxytoxum* (Dinophyceae) with the aid of scanning electron microscopy. *Bot. Mar.* **28**, 99–122 (1985).

35 Brummitt, R. K. & Powell, C. E. *Authors of plant names: A list of authors of scientific names of plants, with recommended standard forms of their names, including abbreviations*. (Royal Botanic Gardens, 1992).

36 Coats, D. W., Kim, S., Bachvaroff, T. R., Handy, S. M. & Delwiche, C. F. *Tintinnophagus acutus* n. g., n. sp. (Phylum Dinoflagellata), an ectoparasite of the ciliate *Tintinnopsis cylindrica* Daday 1887, and its relationship to *Duboscquodinium collini* Grassé 1952. *J. Eukaryot. Microbiol.* **57**, 468–482 (2010).

37 Janouškovec, J. *et al.* Colponemids represent multiple ancient alveolate lineages. *Curr. Biol.* **23**, 2546–2552 (2013).

38 Zhu, G., Keithly, J. S. & Philippe, H. What is the phylogenetic position of *Cryptosporidium*? *Int. J. Syst. Evol. Microbiol.* **50**, 1673–1681 (2000).

39 Bachvaroff, T. R., Kim, S., Guillou, L., Delwiche, C. F. & Coats, D. W. Molecular diversity of the syndinean genus *Euduboscquella* based on single-cell PCR analysis. *Appl. Environ. Microbiol.* **78**, 334–345 (2012).

40 Hanif, A. W. *et al.* Variation in spatial and temporal incidence of the crustacean pathogen *Hematodinium perezi* in environmental samples from Atlantic Coastal Bays. *Aquat. Biosyst.* **9**, 11 (2013).

41 Hewitt, E. A. *et al.* Phylogenetic relationships among 28 spirotrichous ciliates documented by rDNA. *Mol. Phylogenet. Evol.* **29**, 258–267 (2003).

42 Massana, R., Karniol, B., Pommier, T., Bodaker, I. & Beja, O. Metagenomic retrieval of a ribosomal DNA repeat array from an uncultured marine alveolate. *Environ. Microbiol.* **10**, 1335–1343 (2008).

43 Nowacki, M. *et al.* A functional role for transposases in a large eukaryotic genome. *Science* **324**, 935–938 (2009).

44 Pecher, W. T., Robledo, J. A. F. & Vasta, G. R. Identification of a second rRNA gene unit in the *Perkinsus andrewsi* genome. *J. Eukaryot. Microbiol.* **51**, 234–245 (2004).

45 Robledo, J. A. F., Nunes, P. A., Cancela, M. L. & Vasta, G. R. Development of an in vitro clonal culture and characterization of the rRNA gene cluster of *Perkinsus atlanticus*, a protistan parasite of the clam *Tapes decussatus*. *J. Eukaryot. Microbiol.* **49**, 414–422 (2002).

46 Okamoto, N., Horak, A. & Keeling, P. J. Description of two species of early branching dinoflagellates, *Psammosa pacifica* n. g., n. sp. and *P. atlantica* n. sp. *PLoS One* **7**, e34900 (2012).

47 Skovgaard, A., Massana, R., Balagué, V. & Saiz, E. Phylogenetic position of the copepod-infesting parasite *Syndinium turbo* (Dinoflagellata, Syndinea). *Protist* **156**, 413–423 (2005).

48 Gardner, M. J. *et al.* Genome sequence of *Theileria parva*, a bovine pathogen that transforms lymphocytes. *Science* **309**, 134–137 (2005).

49 Janouškovec, J., Horák, A., Oborník, M., Lukeš, J. & Keeling, P. J. A common red algal origin of the apicomplexan, dinoflagellate, and heterokont plastids. *Proc. Natl Acad. Sci. USA* **107**, 10949–10954 (2010).

50 Cooney, E. C. *et al.* Single-cell transcriptomics of *Abedinium* reveals a new early-branching dinoflagellate lineage. *Genome Biol. Evol.* **12**, 2417–2428 (2020).

51 Gómez, F., Kiriakoulakis, K. & Lara, E. *Achradina pulchra*, a unique dinoflagellate (Amphilothales, Dinophyceae) with a radiolarian-like endoskeleton of celestite (strontium sulfate). *Acta Protozool.* **56**, 71–76 (2017).

52 Gast, R. J. & Caron, D. A. Molecular phylogeny of symbiotic dinoflagellates from planktonic Foraminifera and Radiolaria. *Mol. Biol. Evol.* **13**, 1192–1197 (1996).

53 Ki, J.-S. & Han, M.-S. Cryptic long internal repeat sequences in the ribosomal DNA ITS1 gene of the dinoflagellate *Cochlodinium polykrikoides* (Dinophyceae): A 101 nucleotide six-repeat track with a palindrome-like structure. *Genes Genet. Syst.* **82**, 161–166 (2007).

54 Nézan, E. *et al.* Taxonomic revision of the dinoflagellate *Amphidoma caudata*: Transfer to the genus *Azadinium* (Dinophyceae) and proposal of two varieties, based on morphological and molecular phylogenetic analyses. *J. Phycol.* **48**, 925–939 (2012).

55 Tillmann, U., Gottschling, M., Nézan, E., Krock, B. & Bilien, G. Morphological and molecular characterization of three new *Azadinium* species (Amphidomataceae, Dinophyceae) from the Irminger Sea. *Protist* **165**, 417–444 (2014).

56 Luo, Z. H. *et al.* Sympatric occurrence of two *Azadinium poporum* ribotypes in the Eastern Mediterranean Sea. *Harmful Algae* **78**, 75–85 (2018).

57 Tillmann, U., Söhner, S., Nézan, E. & Krock, B. First record of the genus *Azadinium* (Dinophyceae) from the Shetland Islands, including the description of *Azadinium polongum* sp. nov. *Harmful Algae* **20**, 142–155 (2012).

58 Borchhardt, N. *et al.* Morphology and molecular phylogeny of *Bindiferia* gen. nov. (Dinophyceae), a new marine, sand-dwelling dinoflagellate genus formerly classified within *Amphidinium*. *Phycologia* **60**, 631–643 (2021).

59 Luo, Z. *et al.* Morpho-molecular diversity and phylogeny of *Bysmatrum* (Dinophyceae) from the South China Sea and France. *Eur. J. Phycol.* **53**, 318–335 (2018).

60 Gottschling, M. *et al.* Delimitation of the Thoracosphaeraceae (Dinophyceae), including the calcareous dinoflagellates, based on large amounts of ribosomal RNA sequence data. *Protist* **163**, 15–24 (2012).

61 Luo, Z. *et al.* Morphology, ultrastructure and molecular phylogeny of cyst-producing *Caladoa arcachonensis* gen. et sp. nov. (Peridiniales, Dinophyceae) from France and Indonesia. *Eur. J. Phycol.* **54**, 235–248 (2019).

62 Reñé i Vicente, A., de Salas, M., Camp, J., Balagué, V. & Garcés, E. A new clade, based on partial LSU rDNA sequences, of unarmoured dinoflagellates. *Protist* **164**, 673–68 (2013).

63 Iwataki, M. *et al.* Phylogenetic relationships in the harmful dinoflagellate *Cochlodinium polykrikoides* (Gymnodiniales, Dinophyceae) inferred from LSU rDNA sequences. *Harmful Algae* **7**, 271–277 (2008).

64 Howard, M. D. A. *et al.* Quantitative real-time PCR for *Cochlodinium fulvescens* (Dinophyceae), a potentially harmful dinoflagellate from California Coastal waters. *J. Phycol.* **48**, 384–393 (2012).

65 Gómez, F., Richlen, M. L. & Anderson, D. M. Molecular characterization and morphology of *Cochlodinium strangulatum*, the type species of *Cochlodinium*, and *Margalefidinium* gen. nov. for *C. polykrikoides* and allied species (Gymnodiniales, Dinophyceae). *Harmful Algae* **63**, 32–44 (2017).

66 Gottschling, M. *et al.* *Fensomea setacea*, gen. & sp. nov. (Cladopyxidaceae, Dinophyceae), is neither gonyaulacoid nor peridinioid as inferred from morphological and molecular data. *Sci. Rep.* **11**, 12824–12824 (2021).

67 Saldarriaga Echavarría, J. F., Taylor, F. J. R., Keeling, P. J. & Cavalier-Smith, T. Dinoflagellate nuclear SSU rRNA phylogeny suggests multiple plastid losses and replacements. *J. Mol. Evol.* **53**, 204–213 (2001).

68 Logares, R., Shalchian-Tabrizi, K., Boltovskoy, A. & Rengefors, K. Extensive dinoflagellate phylogenies indicate infrequent marine-freshwater transitions. *Mol. Phylogenet. Evol.* **45**, 887–903 (2007).

69 Wakeman, K. C., Yamaguchi, A. & Horiguchi, T. Molecular phylogeny and morphology of *Haplozoon ezoense* n. sp. (Dinophyceae): A parasitic dinoflagellate with ultrastructural evidence of remnant non-photosynthetic plastids. *Protist* **169**, 333–350 (2018).

70 Saldarriaga Echavarría, J. F., Taylor, F. J. R., Cavalier-Smith, T., Menden-Deuerd, S. & Keeling, P. J. Molecular data and the evolutionary history of dinoflagellates. *Eur. J. Protistol.* **40**, 85–111 (2004).

71 Boutrup, P. V., Moestrup, Ø., Tillmann, U. & Daugbjerg, N. Ultrastructure and phylogeny of *Kirithra asteri* gen. et sp. nov. (Ceratoperidiniaceae, Dinophyceae) — A free-living, thin-walled marine photosynthetic dinoflagellate from Argentina. *Protist* **168**, 586–611 (2017).

72 Hu, Z. X. *et al.* Morphology, ultrastructure, and molecular phylogeny of the unarmoured dinoflagellate *Kirithra sigma* sp. nov. (Ceratoperidiniaceae, Dinophyceae). *Phycologia* **59**, 385–396 (2020).

73 Moestrup, Ø. & Daugbjerg, N. in *Unravelling the algae, the past, present, and future of algal systematics Systematics Association Special Volume **75*** (eds J. Brodie & J. Lewis) 215–230 (CRC Press, 2007).

74 Stern, R. F. *et al.* Evaluating the ribosomal Internal Transcribed Spacer (ITS) as a candidate dinoflagellate barcode marker. *PLoS One* **7**, e42780 (2012).

75 Herrera Sepúlveda, A., Hernandez-Saavedra, N. Y., Medlin, L. K. & West, N. Capillary electrophoresis finger print technique (CE-SSCP): An alternative tool for the monitoring

activities of HAB species in Baja California Sur Costal. *Environ. Sci. Pollut. Res.* **20**, 6863–6871 (2013).

76 Prabowo, D. A., Shah, M. M. R., Horiguchi, T. & Suda, S. Genetic diversity of *Moestrupia oblonga* (Dinophyceae) from coastal areas of Okinawa Island, Japan. *Mar. Biodivers.* **46**, 197–209 (2016).

77 Ki, J.-S. Nuclear 28S rDNA phylogeny supports the basal placement of *Noctiluca scintillans* (Dinophyceae; Noctilucales) in dinoflagellates . *Eur. J. Protistol.* **46**, 111–120 (2010).

78 Hu, Z. *et al.* Characterization of the unarmored dinoflagellate *Pseliodinium pirum* (Ceratoperidiniaceae) from Jiaozhou Bay, China. *Phycol. Res.* **68**, 3–13 (2020).

79 Jørgensen, M. F., Murray, S. A. & Daugbjerg, N. A new genus of athecate interstitial dinoflagellates, *Togula* gen. nov., previously encompassed within *Amphidinium sensu lato*: Inferred from light and electron microscopy and phylogenetic analyses of partial large subunit ribosomal DNA sequences. *Phycol. Res.* **52**, 284–299 (2004).

80 Fawcett, R. C. & Parrow, M. W. Cytological and phylogenetic diversity in freshwater *Esoptrodinium*/*Bernardinium* species (Dinophyceae). *J. Phycol.* **48**, 793–807 (2012).

81 Luo, Z., You, X., Mertens, K. N. & Gu, H. Morphological and molecular characterization of *Tovellia* cf. *aveirensis* (Dinophyceae) from Jiulong River, China. *Nova Hedwigia* **103**, 79–94 (2016).

82 Murray, S. A., Garby, T., Hoppenrath, M. & Neilan, B. A. Genetic diversity, morphological uniformity and polyketide production in dinoflagellates (*Amphidinium*, Dinoflagellata). *PLoS One* **7**, e38253 (2012).

83 Lee, K. H. *et al.* Morphology and molecular characterization of the epiphytic dinoflagellate *Amphidinium massartii*, isolated from the temperate waters off Jeju Island, Korea. *Algae* **28**, 213–231 (2013).

84 Luo, Z. H. *et al.* Characterization of *Amphidinium* (Amphidiniales, Dinophyceae) species from the China Sea based on morphological, molecular, and pigment data. *J. Oceanol. Limnol.* **40**, 1191–1219 (2022).

85 Luo, Z. *et al.* *Amphidinium stirisquamtum* sp. nov. (Dinophyceae), a new marine sand-dwelling dinoflagellate with a novel type of body scale. *Algae* **36**, 241–26 (2021).

86 Gu, H. *et al.* Cyst-motile stage relationship, morphology, ultrastructure, and molecular phylogeny of the gymnodinioid dinoflagellate *Barrufeta resplendens* comb. nov., formerly known as *Gyrodinium resplendens*, isolated from the Gulf of Mexico. *J. Phycol.* **51**, 990–999 (2015).

87 Wang, N. *et al.* Cyst-motile stage relationship and molecular phylogeny of a new freshwater dinoflagellate *Gymnodinium plasticum* from Plastic Lake, Canada. *Phycol. Res.* **65**, 312–321 (2017).

88 Yamada, N., Terada, R., Tanaka, A. & Horiguchi, T. *Bispinodinium angelaceum* gen. et sp. nov. (Dinophyceae), a new sand-dwelling dinoflagellate from the seafloor off Mageshima Island, Japan. *J. Phycol.* **49**, 555–569 (2013).

89 Kim, K.-Y., Iwataki, M. & Kim, C.-H. Molecular phylogenetic affiliations of *Dissodinium pseudolunula*, *Pheopolykrikos hartmannii*, *Polykrikos* cf. *schwartzii* and *Polykrikos kofoidii* to *Gymnodinium sensu stricto* species (Dinophyceae). *Phycol. Res.* **56**, 89–92 (2008).

90 Ki, J.-S. & Han, M.-S. Informative characteristics of 12 divergent domains in complete large subunit rDNA sequences from the harmful dinoflagellate genus, *Alexandrium* (Dinophyceae). *J. Eukaryot. Microbiol.* **54**, 210–219 (2007).

91 Sundström, A. M. *et al.* *Gymnodinium corollarium* sp. nov. (Dinophyceae)—A new cold-water dinoflagellate responsible for cyst sedimentation events in the Baltic Sea. *J. Phycol.* **45**, 938–952 (2009).

92 Annenkova, N. V., Hansen, G. & Rengefors, K. Closely related dinoflagellate species in vastly different habitats – An example of amarine–freshwater transition. *Eur. J. Phycol.* **55**, 478–489 (2020).

93 Romeikat, C., Knechtel, J. & Gottschling, M. Clarifying the taxonomy of *Gymnodinium fuscum* var. *rubrum* from Bavaria (Germany) and placing it in a molecular phylogeny of the Gymnodiniaceae (Dinophyceae). *Syst. Biodivers.* **18**, 102–115 (2020).

94 Kang, N. S. *et al.* *Gymnodinium smaydae* n. sp., a new planktonic phototrophic dinoflagellate from the coastal waters of Western Korea: Morphology and molecular characterization. *J. Eukaryot. Microbiol.* **61**, 182–203 (2014).

95 Ishitani, Y., Ujiie, Y. & Takishita, K. Uncovering sibling species in Radiolaria: Evidence for ecological partitioning in a marine planktonic protist. *Mol. Phylogenet. Evol.* **78**, 215–222 (2014).

96 Luo, Z. *et al.* Morphology, ultrastructure, and molecular phylogeny of *Wangodinium sinense* gen. et sp. nov. (Gymnodiniales, Dinophyceae) and revisiting of *Gymnodinium dorsalisulcum* and *Gymnodinium impudicum*. *J. Phycol.* **54**, 744–761 (2018).

97 Hoppenrath, M., Bachvaroff, T. R., Handy, S. M., Delwiche, C. F. & Leander, B. S. Molecular phylogeny of ocelloid-bearing dinoflagellates (Warnowiaceae) as inferred from SSU and LSU rDNA sequences. *BMC Evol. Biol.* **9**, 116 (2009).

98 Takano, Y., Yamaguchi, H., Inouye, I., Moestrup, Ø. & Horiguchi, T. Phylogeny of five species of *Nusuttodinium* gen. nov. (Dinophyceae), a genus of unarmoured kleptoplastidic dinoflagellates. *Protist* **165**, 759–778 (2014).

99 Yokouchi, K. & Horiguchi, T. *Paragymnodinium verecundum* sp. nov. (Gymnodiniales, Dinophyceae), a new species of mixotrophic dinoflagellate from Japan. *Phycol. Res.* **69**, 124–136 (2021).

100 Tang, Y. Z., Harke, M. J. & Gobler, C. J. Morphology, phylogeny, dynamics, and ichthyotoxicity of *Pheopolykrikos hartmannii* (Dinophyceae) isolates and blooms from New York, USA. *J. Phycol.* **49**, 1084–1094 (2013).

101 Kretschmann, J., Filipowicz, N. H., Owsianny, P. M., Zinßmeister, C. & Gottschling, M. Taxonomic clarification of the unusual dinophyte *Gymnodinium limneticum* Wołosz. (Gymnodiniaceae) from the Tatra Mountains. *Protist* **166**, 621–637 (2015).

102 Kim, S., Yoon, J. & Park, M. G. 서해안 동호 사질 조간대에 서식하는 저서성 와편모류의 출현양상 및 분자계통학적 특성. *J. Kor. Soc. Oceanogr.* **20**, 141–150 (2015).

103 Watanabe, K. *et al.* *Ankistrodinium armigerum* sp. nov. (Dinophyceae), a new species of heterotrophic marine sand-dwelling dinoflagellate from Japan and Australia. *Phycol. Res.* **62**, 125–135 (2014).

104 Hoppenrath, M., Murray, S. A., Sparmann, S. F. & Leander, B. S. Morphology and molecular phylogeny of *Ankistrodinium* gen. nov. (Dinophyceae), a new genus of marine sand-dwelling dinoflagellates formerly classified within *Amphidinium*. *J. Phycol.* **48**, 1143–1152 (2012).

105 Jørgensen, M. F., Murray, S. A. & Daugbjerg, N. *Amphidinium* revisited. I. Redefinition of *Amphidinium* (Dinophyceae) based on cladistic and molecular phylogenetic analyses. *J. Phycol.* **40**, 351–365 (2004).

106 Sparmann, S. F., Leander, B. S. & Hoppenrath, M. Comparative morphology and molecular phylogeny of *Apicoporus* n. gen.: A new genus of marine benthic dinoflagellates formerly classified within *Amphidinium*. *Protist* **159**, 383–399 (2008).

107 Benico, G., Takahashi, K., Lum, W. M. & Iwataki, M. Morphological variation, ultrastructure, pigment composition and phylogeny of the star-shaped dinoflagellate *Asterodinium gracile* (Kareniaceae, Dinophyceae). *Phycologia* **58**, 405–418 (2019).

108 Gómez, F., Artigas, L. F. & Gast, R. J. Molecular phylogeny and synonymy of *Balechina gracilis* comb. nov. (=*Gymnodinium gracile*), a widespread polymorphic unarmored dinoflagellate (Dinophyceae). *J. Phycol.* (2021).

109 Takahashi, K., Benico, G., Lum, W. M. & Iwataki, M. *Gertia stigmatica* gen. et sp. nov. (Kareniaceae, Dinophyceae), a new marine unarmored dinoflagellate possessing the peridinin-type chloroplast with an eyespot. *Protist* **170**, 125680 (2019).

110 Gómez, F., Artigas, L. F. & Gast, R. J. Phylogeny and synonymy of *Gyrodinium heterostriatum* comb. nov. (Dinophyceae), a common unarmored dinoflagellate in the world cceans. *Acta Protozool.* **59**, 77–98 (2020).

111 Hansen, G. & Daugbjerg, N. Ultrastructure of *Gyrodinium spirale*, the type species of *Gyrodinium* (Dinophyceae), including a phylogeny of *G. dominans*, *G. rubrum* and *G. spirale* deduced from partial LSU rDNA sequences. *Protist* **155**, 271–294 (2004).

112 Takano, Y. & Horiguchi, T. Surface ultrastructure and molecular phylogenetics of four unarmored heterotrophic dinoflagellates, including the type species of the genus *Gyrodinium* (Dinophyceae). *Phycol. Res.* **52**, 107–116 (2004).

113 Boutrup, P. V., Moestrup, Ø., Tillmann, U. & Daugbjerg, N. *Katodinium glaucum* (Dinophyceae) revisited: Proposal of new genus, family and order based on ultrastructure and phylogeny. *Phycologia* **55**, 147–164 (2016).

114 Fan, Y. *et al.* Study of genetic diversity of micro-planktonic eukaryotes in South China Sea by ITS and 5.8s rRNA gene cloning and sequencing. *Int. J. Simul. Syst. Sci. Techn.* **16**, 20.21–20.10 (2015).

115 Loret, P. *et al.* No difference found in ribosomal DNA sequences from physiologically diverse clones of *Karenia brevis* (Dinophyceae) from the Gulf of Mexico. *J. Plankton Res.* **24**, 735–739 (2002).

116 Mikulski, C. M., Morton, S. L. & Doucette, G. J . Development and application of LSU rRNA probes for *Karenia brevis* in the Gulf of Mexico, USA. *Harmful Algae* **4**, 49–60 (2005).

117 Zhang, H., Bhattacharya, D. & Lin, S. A three-gene dinoflagellate phylogeny suggests monophyly of Prorocentrales and a basal position for *Amphidinium* and *Heterocapsa*. *J. Mol. Evol.* **65**, 463–474 (2007).

118 Luo, Z. H., Wang, L., Chan, L., Lu, S. H. & Gu, H. *Karlodinium zhouanum*, a new dinoflagellate species from China, and molecular phylogeny of *Karenia digitata* and *Karenia longicanalis* (Gymnodiniales, Dinophyceae). *Phycologia* **57** (2018).

119 Gómez, F., Qiu, D., Dodge, J. D., Lopes, R. M. & Lin, S. Morphological and molecular characterization of *Ptychodiscus noctiluca* revealed the polyphyletic nature of the order Ptychodiscales (Dinophyceae). *J. Phycol.* (2016).

120 Ok, J. H., Jeong, H. J., Lee, S. Y., Park, S. A. & Noh, J. H. *Shimiella* gen. nov. and *Shimiella gracilenta* sp. nov. (Dinophyceae, Kareniaceae), a kleptoplastidic dinoflagellate from Korean waters and its survival under starvation. *J. Phycol.* **57**, 70–91 (2021).

121 Henrichs, D. W., Sosik, H. M., Olson, R. J. & Campbell, L. Phylogenetic analysis of *Brachidinium capitatum* (Dinophyceae) from the Gulf of Mexico indicates membership in the Kareniaceae. *J. Phycol.* **47**, 366–374 (2011).

122 Gómez, F., Takayama, H., Moreira, D. & López-García, P. Unarmoured dinoflagellates with a small hyposome: *Torodinium* and *Lebouridinium* gen. nov for *Katodinium glaucum* (Gymnodiniales, Dinophyceae). *Eur. J. Phycol.* **51**, 226–241 (2016).

123 Jeong, H. J. *et al.* *Ansanella granifera* gen. et sp. nov (Dinophyceae), a new dinoflagellate from the coastal waters of Korea. *Algae* **29**, 75–99 (2014).

124 Hansen, G., Daugbjerg, N. & Henriksen, P. *Baldinia anauniensis* gen. et sp. nov.: A 'new' dinoflagellate from Lake Tovel, N. Italy. *Phycologia* **46**, 86–108 (2007).

125 Kremp, A., Elbrächter, M., Schweikert, M., Wolny, J. L. & Gottschling, M. *Woloszynskia halophila* (Biecheler) comb. nov.: A bloom-forming cold-water dinoflagellate co-occurring with *Scrippsiella hangoei* (Dinophyceae) in the Baltic Sea. *J. Phycol.* **41**, 629–642 (2005).

126 Gottschling, M., Renner, S. S., Meier, K. J. S., Willems, H. & Keupp, H. Timing deep divergence events in calcareous dinoflagellates. *J. Phycol.* **44**, 429–438 (2008).

127 Takahashi, K., Moestrup, Ø., Jordan, R. W. & Iwataki, M. Two new freshwater woloszynskioids *Asulcocephalium miricentonis* gen. et sp. nov. and *Leiocephalium pseudosanguineum* gen. et sp. nov. (Suessiaceae, Dinophyceae) lacking an apical furrow apparatus. *Protist* **166**, 638–658 (2015).

128 Shoguchi, E. *et al.* Draft assembly of the *Symbiodinium minutum* nuclear genome reveals dinoflagellate gene structure. *Curr. Biol.* **23**, 1399–1408 (2013).

129 Takahashi, K. *et al.* *Dactylodinium pterobelotum* gen. et sp. nov., a new marine woloszynskioid dinoflagellate positioned between the two families Borghiellaceae and Suessiaceae. *J. Phycol.* **53**, 1223–1240 (2017).

130 Carlos, A. A., Baillie, B. K., Kawachi, M. & Maruyama, T. Phylogenetic position of *Symbiodinium* (Dinophyceae) isolates from tridacnids (Bivalvia), cardiids (Bivalvia), a sponge (Porifera), a soft coral (Anthozoa), and a free-living strain. *J. Phycol.* **35**, 1054–1062 (1999).

131 Pochon, X., Pawlowski, J., Zaninetti, L. & Rowan, R. High genetic diversity and relative specificity among *Symbiodinium*-like endosymbiotic dinoflagellates in soritid foraminiferans. *Mar. Biol.* **139**, 1069–1078 (2001).

132 Jeong, H. J. *et al.* Heterotrophic feeding as a newly identified survival strategy of the dinoflagellate *Symbiodinium*. *Proc. Natl Acad. Sci. USA* **109**, 12604–12609 (2012).

133 Horiguchi, T., Moriya, R., Pinto, S. K. & Terada, R. *Pyramidodinium spinulosum* sp. nov. (Dinophyceae), a sand-dwelling non-motile dinoflagellate from the seafloor (36 m deep) off Mageshima Island, Kagoshima, Japan. *Phycol. Res.* **65**, 272–279 (2017).

134 Pandeirada, M. S., Craveiro, S. C. F., Daugbjerg, N., Moestrup, Ø. & Calado, A. J. Fine-structural characterization and phylogeny of *Sphaerodinium* (Suessiales, Dinophyceae), with the description of an unusual type of freshwater dinoflagellate cyst. *Eur. J. Protistol.* **78** 125770 (2021).

135 Mertens, K. N. *et al.* Morpho-molecular analysis of podolampadacean dinoflagellates (Dinophyceae), with the description of two new genera. *Phycologia* **62**, 117–135 (2023).

136 Gottschling, M. *et al.* Phylogeny of calcareous dinoflagellates as inferred from ITS and ribosomal sequence data. *Mol. Phylogenet. Evol.* **36**, 444–455 (2005).

137 Gottschling, M. & Söhner, S. An updated list of generic names in the Thoracosphaeraceae. *Microorganisms* **1**, 122–136 (2013).

138 Logares, R. *et al.* Phenotypically different microalgal morphospecies with identical ribosomal DNA: A case of rapid adaptive evolution? *Microb. Ecol.* **53**, 549–561 (2007).

139 Ribeiro, S., Lundholm, N., Amorim, A. & Ellegaard, M. *Protoperidinium minutum* (Dinophyceae) from Portugal: Cyst-theca relationship and phylogenetic position on the basis of single-cell SSU and LSU rDNA sequencing. *Phycologia* **49**, 48–63 (2010).

140 Coats, D. W. *et al.* Prevalence and phylogeny of parasitic dinoflagellates (genus *Blastodinium*) infecting copepods in the Gulf of California. *CICIMAR Oceánides* **23**, 67–77 (2008).

141 D´Onofrio, G., Marino, D., Bianco, L., Busico, E. & Montresor, M. Toward an assessment on the taxonomy of dinoflagellates that produce calcareous cysts (Calciodinelloideae, Dinophyceae): A morphological and molecular approach. *J. Phycol.* **35**, 1063–1078 (1999).

142 Montresor, M., Sgrosso, S., Procaccini, G. & Kooistra, W. H. C. F. Intraspecific diversity in *Scrippsiella trochoidea* (Dinophyceae): Evidence for cryptic species. *Phycologia* **42**, 56–70 (2003).

143 Gottschling, M. & Plötner, J. Secondary structure models of the nuclear Internal Transcribed Spacer regions and 5.8S rRNA in Calciodinelloideae (Peridiniaceae) and other dinoflagellates. *Nucleic Acids Res.* **32**, 307–315 (2004).

144 Zinßmeister, C. *et al.* Same but different: Two novel bicarinate species of extant calcareous dinophytes (Thoracosphaeraceae, Peridiniales) from the Mediterranean Sea. *J. Phycol.* **48**, 1107–1118 (2012).

145 Gribble, K. E. & Anderson, D. M. Molecular phylogeny of the heterotrophic dinoflagellates, *Protoperidinium*, *Diplopsalis* and *Preperidinium* (Dinophyceae), inferred from large subunit rDNA. *J. Phycol.* **42**, 1081–1095 (2006).

146 Kretschmann, J., Žerdoner Čalasan, A. & Gottschling, M. Molecular phylogenetics of dinophytes harbouring diatoms as endosymbionts (Kryptoperidiniaceae, Peridiniales), with evolutionary interpretations and a focus on the identity of *Durinskia oculata* from Prague. *Mol. Phylogenet. Evol.* **118**, 392–402 (2018).

147 Yoshida, T. *et al.* Sequence analysis of 5.8S rDNA and the Internal Transcribed Spacer region in dinoflagellate *Heterocapsa* species (Dinophyceae) and development of selective PCR primers for the bivalve killer *Heterocapsa circularisquama*. *Microbes Environ.* **18**, 216–222 (2003).

148 Zhang, Q., Song, H. Y., Hu, Z. Y. & Liu, G. X. Morphological examination and phylogenetic position of the newly recorded heterotrophic brackish dinoflagellate *Diplopsalis caspica* (Dinophyceae) in freshwater habitat from China. *J. Syst. Evol.* **53**, 512–519 (2015).

149 Potvin, É. *et al.* *Islandinium minutum* subsp. *barbatum* subsp. nov. (Dinoflagellata), a new organic-walled dinoflagellate cyst from the Western Arctic: Morphology, phylogenetic position based on SSU rDNA and LSU rDNA, and distribution. *J. Eukaryot. Microbiol.* **65**, 750–772 (2018).

150 Saburova, M. & Chomérat, N. *Laciniporus arabicus* gen. et sp. nov. (Dinophyceae, Peridiniales), a new thecate, marine, sand-dwelling dinoflagellate from the northern Indian Ocean (Arabian Sea). *J. Phycol.* (2018).

151 Saldarriaga Echavarría, J. F., Leander, B. S., Taylor, F. J. R. & Keeling, P. J. *Lessardia elongata* gen. et. sp. nov. (Dinoflagellata, Peridiniales, Podolampaceae) and the taxonomic position of the genus *Roscoffia*. *J. Phycol.* **39**, 368–378 (2003).

152 Leander, B. S. & Keeling, P. J. Early evolutionary history of dinoflagellates and apicomplexans (Alveolata) as inferred from hsp90 and actin phylogenies. *J. Phycol.* **40**, 341–350 (2004).

153 Hansen, G., Daugbjerg, N. & Moestrup, Ø. The rainwater rock-pool dinoflagellate *Nottbeckia ochracea* gen. et comb. nov (syn.: *Hemidinium ochraceum*) - A fine-structural and molecular study with emphasis on the motile stage. *Protist* **169**, 280–306 (2018).

154 Hoppenrath, M., Reñé i Vicente, A., Satta, C. T., Yamaguchi, A. & Leander, B. S. Morphology and molecular phylogeny of a new marine, sand-dwelling dinoflagellate genus, *Pachena* (Dinophyceae), with descriptions of three new species. *J. Phycol.* **56**, 798–817 (2020).

155 Kretschmann, J., Žerdoner Čalasan, A., Kusber, W.-H. & Gottschling, M. Still curling after all these years: *Glenodinium apiculatum* Ehrenb. (Peridiniales, Dinophyceae) repeatedly found at its type locality in Berlin (Germany). *Syst. Biodivers.* **16**, 200–209 (2018).

156 Kretschmann, J., Owsianny, P. M., Žerdoner Čalasan, A. & Gottschling, M. The hot spot in a cold environment: Puzzling *Parvodinium* (Peridiniopsidaceae, Peridiniales) from the Polish Tatra Mountains. *Protist* **169**, 206–230 (2018).

157 Ki, J.-S. & Han, M.-S. Rapid molecular identification of the harmful freshwater dinoflagellate *Peridinium* in various life stages using genus-specific single-cell PCR. *J. Appl. Phycol.* **19**, 467–470 (2007).

158 Ki, J.-S., Park, M.-H. & Han, M.-S. Discriminative power of nuclear rDNA sequences for the DNA taxonomy of the dinoflagellate genus *Peridinium* (Dinophyceae). *J . Phycol.* **47**, 426–435 (2011).

159 Gottschling, M., Kretschmann, J. & Žerdoner Čalasan, A. Description of Peridiniopsidaceae, fam. nov. (Peridiniales, Dinophyceae). *Phytotaxa* **299**, 293–296 (2017).

160 Holzer, V. J. C., Kretschmann, J., Knechtel, J., Owsianny, P. M. & Gottschling, M. Morphological and molecular variability of *Peridinium volzii* Lemmerm. (Peridiniaceae, Dinophyceae) and its relevance for infraspecific taxonomy. *Org. Divers. Evol.* **22**, 1–15 (2022).

161 Saito, K., Drgon, T., Robledo, J. A. F., Krupatkina, D. N. & Vasta, G. R. Characterization of the rRNA locus of *Pfiesteria piscicida* and development of standard and quantitative PCR-based detection assays targeted to the nontranscribed spacer. *Appl. Environ. Microbiol.* **68**, 5394–5407 (2002).

162 Liu, X. H., Liu, Y. Y., Chai, Z. Y., Hu, Z. X. & Tang, Y. Z. A combined approach detected novel species diversity and distribution of dinoflagellate cysts in the Yellow Sea, China. *Mar. Pollut. Bull.* **187**, 114567 (2023).

163 Yamaguchi, A., Kawamura, H. & Horiguchi, T. A further phylogenetic study of the heterotrophic dinoflagellate genus, *Protoperidinium* (Dinophyceae) based on small and large subunit ribosomal RNA gene sequences. *Phycol. Res.* **54**, 317–329 (2006).

164 Yamaguchi, A. & Horiguchi, T. Molecular phylogenetic study of the heterotrophic dinoflagellate genus *Protoperidinium* (Dinophyceae) inferred from small subunit rRNA gene sequences. *Phycol. Res.* **53**, 30-42 (2005).

165 Murray, S. A. *et al.* Phylogenetics of *Rhinodinium broomeense* gen. et sp. nov., a peridinioid, sand-dwelling dinoflagellate (Dinophyceae). *J. Phycol.* **42**, 934–942 (2006).

166 Satta, C. T. *et al.* First detection of the bloom forming Unruhdinium penardii (Dinophyceae) in a Mediterranean reservoir: Insights on its ecology, morphology and genetics. *Adv. Oceanogr. Limnol.* **11**, 71–83 (2020).

167 Gottschling, M. & McLean, T. I. New home for tiny symbionts: Dinophytes determined as *Zooxanthella* are Peridiniales and distantly related to *Symbiodinium*. *Mol. Phylogenet. Evol.* **67**, 217–222 (2013).

168 Saburova, M. & Chomérat, N. *Ailadinium reticulatum* gen. et sp. nov. (Dinophyceae), a new thecate, marine, sand-dwelling dinoflagellate from the northern Red Sea. *J. Phycol.* **50**, 1120–1136 (2014).

169 Jedlicki, A. *et al.* Molecular detection and species identification of *Alexandrium* (Dinophyceae) causing harmful algal blooms along the Chilean coastline. *AoB Plants* **2012**, pls033 (2012).

170 Wang, L., Zhuang, Y., Zhang, H., Lin, X. & Lin, S. DNA barcoding species in *Alexandrium tamarense* complex using ITS and proposing designation of five species. *Harmful Algae* **31**, 100–113 (2014).

171 Ki, J.-S. & Han, M.-S. Efficient 5'ETS walking from conserved 18S rDNA sequences of the dinoflagellates *Alexandrium* and *Akashiwo sanguinea* (Dinophyceae). *J. Appl. Phycol.* **17**, 475–481 (2005).

172 Rogers, J. E., Leblond, J. D. & Moncreiff, C. A. Phylogenetic relationship of *Alexandrium monilatum* (Dinophyceae) to other *Alexandrium* species based on 18S ribosomal RNA gene sequences. *Harmful Algae* **5**, 275–280 (2006).

173 McCauley, L. A. R., Erdner, D. L., Nagai, S., Richlen, M. L. & Anderson, D. M. Biogeographic analysis of the globally distributed algal bloom species *Alexandrium minutum* (Dinophyceae) based on rRNA gene sequences and microsatellite markers. *J. Phycol.* **45**, 454–463 (2009).

174 Xu, Y. X. *et al.* Molecular identification and toxin analysis of *Alexandrium* spp. in the Beibu Gulf: First report of toxic *A. tamiyavanichii* in Chinese coastal waters. *Toxins* **13**, 161 (2021).

175 Penna, A. *et al.* Phylogenetic relationships among the Mediterranean *Alexandrium* (Dinophyceae) species based on sequences of 5.8S gene and Internal Transcript Spacers of the rRNA operon. *Eur. J. Phycol.* **43**, 163–178 (2008).

176 Tillmann, U., Krock, B., Wietkamp, S. & Beran, A. A Mediterranean *Alexandrium taylorii* (Dinophyceae) strain produces doniodomin A and lytic compounds but not paralytic shellfish toxins. *Toxins* **12**, 564 (2020).

177 Hoppenrath, M., Reñé i Vicente, A., Satta, C. T., Yamaguchi, A. & Selina, M. S. Molecular phylogeny and morphology of *Carinadinium* gen. nov. (Dinophyceae, Gonyaulacales), including marine sand-dwelling dinoflagellate species formerly classified within *Thecadinium*. *Eur J Protistol* **81**, 125835 (2021).

178 Jeong, H. J. *et al.* First report of the epiphytic benthic dinoflagellates *Coolia canariensis* and *Coolia malayensis* in the waters off Jeju Island, Korea: Morphology and rDNA sequences. *J. Eukaryot. Microbiol.* **59**, 114–133 (2012).

179 Mertens, K. N., Takano, Y., Head, M. J. & Matsuoka, K. L iving fossils in the Indo-Pacific warm pool: A refuge for thermophilic dinoflagellates during glaciations. *Geology* **42**, 531–534 (2014).

180 Gu, H. *et al.* Unraveling the *Gonyaulax baltica* species complex: Cyst-theca relationship of *Impagidinium variaseptum*, *Spiniferites pseudodelicatus* sp. nov. and *S. ristingensis* (Gonyaulacaceae, Dinophyceae), with descriptions of *Gonyaulax bohaiensis* sp. nov, *G. amoyensis* sp. nov. and *G. portimonensis* sp. nov. *J. Phycol.* **58**, 465–486 (2022).

181 Li, Z., Oh, S. J., Park, J.-W., Lim, W.-A. & Shin, H. H. Cyst-motile stage relationship, morphology and phylogeny of a new chain-forming, marine dinoflagellate *Grammatodinium tongyeonginum* gen. & sp. nov. from Korea. *Phycologia* **56**, 430–443 (2017).

182 Yamada, N., Tanaka, A. & Horiguchi, T. Pigment compositions are linked to the habitat types in dinoflagellates. *J. Plant Res.* **128**, 923–932 (2015).

183 Luo, Z. *et al.* Attributing *Ceratocorys*, *Pentaplacodinium* and *Protoceratium* to Protoceratiaceae (Dinophyceae), with descriptions of *Ceratocorys malayensis sp. nov.* and *Pentaplacodinium usupianum sp. nov.* *Phycologia* **59**, 6–23 (2020).

184 Saunders, G. W., Hill, D. R. A., Sexton, J. P. & Andersen, R. A. Small-subunit ribosomal RNA sequences from selected dinoflagellates: Testing classic evolutionary hypotheses with molecular systematic methods. *Pl. Syst. Evol. (Suppl.)* **11**, 237–259 (1997).

185 Gómez, F., Qiu, D., Lopes, R. M. & Lin, S. *Fukuyoa paulensis* gen. et sp. nov., a new genus for the globular species of the dinoflagellate *Gambierdiscus* (Dinophyceae). *PLoS One* **10**, e0119676 (2015).

186 Mertens, K. N. *et al.* Cyst-theca relationship and phylogenetic position of *Impagidinium caspienense* incubated from Caspian Sea surface sediments: Relation to *Gonyaulax baltica* and evidence for heterospory within gonyaulacoid dinoflagellates. *J. Eukaryot. Microbiol.* **64**, 829–842 (2017).

187 Zhang, W. *et al.* Reclassification of *Gonyaulax verior* (Gonyaulacales, Dinophyceae) as *Sourniaea diacantha gen. et comb. nov.* *Phycologia* **59**, 246–260 (2020).

188 Gu, H. *et al.* Cyst-theca relationships of *Spiniferites bentorii*, *S. hyperacanthus*, *S. ramosus*, *S. scabratus* and molecular phylogenetics of *Spiniferites* and *Tectatodinium* (Gonyaulacales, Dinophyceae). *Phycologia* **60**, 332–353 (2021).

189 Selina, M. S., Efimova, K. V. & Hoppenrath, M. Redefinition of the genus *Thecadinium* (Dinophyceae) using morphological and molecular data, and description of *Thecadinium pseudokofoidii* sp. nov. *Phycologia* **58**, 36–50 (2019).

190 Hoppenrath, M., Saldarriaga Echavarría, J. F., Schweikert, M., Elbrächter, M. & Taylor, F. J. R. Description of *Thecadinium mucosum* sp. nov. (Dinophyceae), a new sand-dwelling marine dinoflagellate, and an emended description of *Thecadinium inclinatum* Balech. *J. Phycol.* **40**, 946–961 (2004).

191 Hoppenrath, M. & Leander, B. S. Dinoflagellate phylogeny as inferred from Heat Shock Protein 90 and ribosomal gene sequences. *PLoS One* **5**, e13220 (2010).

192 Lin, S., Zhang, H., Hou, Y., Miranda, L. & Bhattacharya, D. Development of a dinoflagellate-oriented PCR primer set leads to detection of picoplanktonic dinoflagellates from Long Island Sound. *Appl. Environ. Microbiol.* **72**, 5626–5630 (2006).

193 Leblond, J. D. *et al.* A data mining approach to dinoflagellate clustering according to sterol composition: Correlations with evolutionary history. *Int. J. Data Min. Bioinform.* **4**, 431–451 (2010).

194 Scorzetti, G. *et al.* Multiple simultaneous detection of Harmful Algal Blooms (HABs) through a high throughput bead array technology, with potential use in phytoplankton community analysis. *Harmful Algae* **8**, 196–211 (2009).

195 Jensen, M. H. & Daugbjerg, N. Molecular phylogeny of selected species of the order Dinophysiales (Dinophyceae)—Testing the hypothesis of a dinophysioid radiation. *J. Phycol.* **45**, 1136–1152 (2009).

196 Daugbjerg, N., Jensen, M. H. & Hansen, P. J. Using nuclear-encoded LSU and SSU rDNA sequences to identify the eukaryotic endosymbiont in *Amphisolenia bidentata* (Dinophyceae). *Protist* **164**, 411–422 (2013).

197 Handy, S. M. *et al.* Phylogeny of four Dinophysiacean genera (Dinophyceae, Dinophysiales) based on rDNA sequences from single cells and environmental samples. *J. Phycol.* **45**, 1163–1174 (2009).

198 Gu, H. *et al.* *Adenoides sinensis*, a new sand-dwelling dinoflagellate species from China and reexamination of *Adenoides eludens* from an Atlantic strain. *Phycologia* **57**, 179–190 (2018).

199 Yamada, N., Dawut, M., Terada, R. & Horiguchi, T. *Plagiodinium ballux* sp. nov. (Dinophyceae), a deep (36 m) sand dwelling dinoflagellate from subtropical Japan. *Phycol. Res.* **67**, 12–20 (2019).

200 Gómez, F., Wakeman, K. C., Yamaguchi, A. & Nozaki, H. Molecular phylogeny of the marine planktonic dinoflagellate *Oxytoxum* and *Corythodinium* (Peridiniales, Dinophyceae). *Acta Protozool.* **55**, 239–248 (2016).

201 Chomérat, N. & Bilien, G. *Madanidinium loirii* gen. et sp nov. (Dinophyceae), a new marine benthic dinoflagellate from Martinique Island, Eastern Caribbean. *Eur. J. Phycol.* **49**, 165–178 (2014).

202 Tamura, M. & Horiguchi, T. *Pileidinium ciceropse* gen. et sp. nov. (Dinophyceae), a sand-dwelling dinoflagellate from Palau. *Eur. J. Phycol.* **40**, 281–291 (2005).

203 Gómez, F., Nakamura, Y. & Artigas, L. F. Molecular phylogeny of the sand-dwelling dinoflagellate *Planodinium* striatum and *Chrysodinium* gen. nov. for *Plagiodinium ballux* (Dinophyceae). *Acta Protozool.* **58**, 115–124 (2019).

204 Herrera Sepúlveda, A. *et al.* Are *Prorocentrum hoffmannianum* and *Prorocentrum belizeanum* (Dinophyceae, Prorocentrales), the same species? An integration of morphological and molecular data. *J. Phycol.* **51**, 173–188 (2015).

205 Hoppenrath, M. *et al.* Taxonomy and phylogeny of the benthic *Prorocentrum* species (Dinophyceae)—A proposal and review. *Harmful Algae* **27**, 1–28 (2013).

206 Luo, Z. *et al.* Morphology, molecular phylogeny and okadaic acid production of epibenthic *Prorocentrum* (Dinophyceae) species from the northern South China Sea. *Algal Res.* **22**, 14–30 (2017).

207 Chomérat, N. *et al.* *Prorocentrum glenanicum* sp. nov. and *Prorocentrum pseudopanamense* sp. no.v (Prorocentrales, Dinophyceae), two new benthic dinoflagellate species from South Brittany (northwestern France). *Phycologia* **50**, 202–214 (2011).

208 Hoppenrath, M. *et al.* Taxonomy and phylogeny of the benthic Prorocentrum species (Dinophyceae)—A proposal and review. *Harmful Algae* **27**, 1–28 (2013).

209 Faust, M. A., Vandersea, M. W., Kibler, S. R., Tester, P. A. & Litaker, R. W. *Prorocentrum levis*, a new benthic species (dinophyceae) from a mangrove island, Twin Cays, Belize. *J. Phycol.* **44**, 232–240 (2008).

210 Murray, S. A., Ip, C. L. C., Moore, R., Nagahama, Y. & Fukuyo, Y. Are prorocentroid dinoflagellates monophyletic? A study of 25 species based on nuclear and mitochondrial genes. *Protist* **160**, 245–264 (2009).

211 Nagahama, Y., Murray, S. A., Tomaru, A. & Fukuyo, Y. Species boundaries in the toxic dinoflagellate *Prorocentrum lima* (Dinophyceae, Prorocentrales), based on morphological and phylogenetic characters. *J. Phycol.* **47**, 178–189 (2011).

212 Tillmann, U., Hoppenrath, M. & Gottschling, M. Reliable determination of *Prorocentrum micans* Ehrenb. (Prorocentrales, Dinophyceae) based on newly collected material from the type locality. *Eur. J. Phycol.* **54**, 417–431 (2019).

213 Wu, Y. X. *et al.* Cryptic speciation of benthic *Prorocentrum* (Dinophyceae) species and their potential as ecological indicators. *J. Sea Res.* **190**, 102304 (2022).

214 Hoppenrath, M., Yubuki, N., Stern, R. & Leander, B. S. Ultrastructure and molecular phylogenetic position of a new marine sand-dwelling dinoflagellate from British Columbia, Canada: *Pseudadenoides polypyrenoides* sp. nov. (Dinophyceae). *Eur. J. Phycol.* **52**, 208–224 (2017).
